# Understanding the Mechanisms of Human Liver Regeneration via Characterization of Circulating Extracellular Vesicles

**DOI:** 10.1155/bmri/3824534

**Published:** 2026-03-08

**Authors:** Yilin Sun, Elisa Pasini, Anh Thu Nguyen-Lefebvre, Mamatha Bhat

**Affiliations:** ^1^ Ajmera Transplant Centre, University Health Network, Toronto, Canada, uhn.ca; ^2^ Institute of Medical Sciences, University of Toronto, Toronto, Canada, utoronto.ca; ^3^ Toronto General Hospital Research Institute, Toronto, Canada, uhn.ca; ^4^ Division of Gastroenterology & Hepatology, University of Toronto, Toronto, Canada, utoronto.ca

**Keywords:** extracellular vesicles, liver regeneration, liver transplantation, microRNA

## Abstract

**Aim:**

Our study is aimed at identifying circulating extracellular vesicles associated with liver regeneration in humans and mice, exploring their roles, and evaluating their content as potential biomarkers of regeneration.

**Methods:**

Plasma samples were collected from 12 human liver transplant recipients and 22 mice at distinct time points postsurgery, corresponding to regenerating and nonregenerating phases. Small extracellular vesicles were isolated from plasma, and miRNA was extracted for analysis using NanoString technology. To understand the role of miRNAs in liver regeneration, we utilized bioinformatics tools, including mirDIP and STRING, to identify target genes involved in regenerative signaling pathways, focusing on the Hippo and cell cycle pathways.

**Results:**

Twenty‐five differentially expressed miRNAs were identified from human patient plasma samples and 30 from mouse plasma samples. Bioinformatic analysis revealed a significant overlap between the predicted target genes of these miRNAs and genes involved in critical regenerative pathways, such as the cell cycle and Hippo signaling, suggesting that these miRNAs play important regulatory roles during liver regeneration.

**Conclusions:**

The miRNA profiles associated with small extracellular vesicles have the potential to serve as noninvasive biomarkers for liver regeneration. Further validation of these miRNA‐based biomarkers could advance diagnostic and therapeutic approaches for liver injury and posttransplant recovery.

**Lay Summary**

We investigated how tiny particles called small extracellular vesicles (sEVs), found in the blood, help the liver to heal and regenerate after surgery. By studying both humans and mice, we identified specific molecules, called microRNAs (miRNAs), carried by these vesicles that are linked to key repair processes in the liver. These findings suggest that sEVs and their miRNAs could have the potential to be used as simple blood tests to monitor liver recovery.

## 1. Introduction

The liver is a vital organ responsible for digestion, metabolism, and detoxification, with a remarkable ability to regenerate after injury. Under normal conditions, liver cells rarely divide; however, following partial hepatectomy (PH) or living donor liver transplantation (LDLT), where up to 70% of the liver is removed, the liver regenerates to near‐normal volume within 3 months [1, 2].

Murine models, particularly 2/3 PH, provide insights into this process, with regeneration peaking within 24–48 h and concluding around days 7–10 [3]. Liver regeneration progresses through initiation, progression, and termination phases, regulated by pathways such as Hippo, Hedgehog (Hh), WNT‐*β*‐catenin, and IL‐6/Jak/STAT3 [4].

LDLT expands the organ donor pool and offers a unique context to study human liver regeneration, as both donor and recipient livers regenerate to near‐original size. This study is aimed at identifying circulating biomarkers of regeneration, focusing on miRNAs in sEVs, and validating findings using transcriptomic data from murine models.

## 2. Methods

### 2.1. Study Design

Twenty‐four plasma samples were obtained from the University Health Network (UHN) Multi‐Organ Transplant (MOT) Biobank, collected from LDLT recipients at two time points postsurgery. The first time point was approximately 1 month postsurgery, a period associated with active liver regeneration, and the second time point was up to 3 months postsurgery, when the regenerative process was terminating. All the research was conducted in accordance with both the Declarations of Helsinki and Istanbul. Our study complied with all ethical regulations. Patients provided written consent. The Research Ethics Board at the UHN approved this study (REB# 17‐5311). Inclusion criteria for this study required adult recipients of LDLT with plasma samples available in the MOT Biobank and corresponding liver biopsies demonstrating normal regeneration within the first 3 months posttransplant. Required primary diagnosis was alcoholic cirrhosis and being under standard immunosuppressive therapy, comprising a calcineurin inhibitor (either tacrolimus or cyclosporine), mycophenolate mofetil, and prednisone at 1 month posttransplant. Exclusion criteria included elevated liver enzyme levels, specifically alanine aminotransferase (ALT) greater than 63 U/L and aspartate aminotransferase (AST) greater than 37 U/L, as well as total bilirubin levels below 31 *μ*mol/L. Samples were also excluded if the interval between paired blood collections exceeded 71 days. The mean age at the time of transplant was 61 years, and the other clinical characteristics are reported in Table S1.

### 2.2. Animal Care

All C57BL/6J mice were obtained from Jackson Laboratories (Bar Harbor, Maine, United States) and housed in a specific pathogen‐free environment at the Animal Resources Center, Toronto, Ontario. All animal procedures were approved by the Canadian Council on Animal Care and conducted under Protocol AUP 6045. Mice were maintained in standard cages at 22°C on a 12:12‐h light–dark cycle with ad libitum access to chow and water.

### 2.3. Animal Surgery and Tissue Collection

The 2/3 PH procedure, anesthesia, and postoperative care were performed as previously described in Mitchell and Willenbring [5] and are detailed in Nguyen et al. [6]. Briefly, mice underwent resection of the median and left lateral liver lobes, whereas sham‐operated controls received identical anesthesia and surgical exposure without resection. Postoperative care included ad libitum access to chow and water, with no antibiotics.

For tissue and blood collection, blood was drawn from live mice via the saphenous vein into EDTA‐coated tubes. At endpoint, mice were euthanized under isoflurane, and livers were collected using a sterile technique. Tissues were processed for downstream analysis by snap freezing, RNAlater preservation, or formalin fixation.

All experimental procedures adhered to the ARRIVE 2.0 guidelines for reporting animal research, and all relevant details required by these guidelines are provided in full in Nguyen et al. [6] publication.

### 2.4. Mouse Plasma Samples

Liver regeneration in mice post‐PH peaks at 24–36 h, and after the majority of liver mass has been restored, the termination phase of liver regeneration takes place from Days 7–10 [3]. Mouse plasma samples were collected from our in *vivo* mouse liver regeneration model at 2 and 7 days post‐PH to represent the acute and termination phases of regeneration, respectively, as previously described [6]. A total of 22 samples were collected, with 12 obtained at 2 days and 10 at 7 days post‐PH. Among these, 13 samples were derived from female mice, whereas nine were from male mice. For sEV isolation, RNA extraction, and NanoString protocols, the same procedures were followed as with human plasma samples.

### 2.5. Extracellular Vesicle Isolation

The Exosome Isolation Kit (from plasma) from Invitrogen (Thermo Fisher Scientific, Cat. No. 4484450) was used to isolate sEVs from patient plasma samples following the manufacturer′s protocol. This kit was selected based on prior reports demonstrating its superior yield compared with other commercial kits, such as Wako and iZON [7]. It employs a polymer precipitation method, in which an exosome isolation reagent induces aggregation of sEVs, allowing for their collection via low‐speed centrifugation while contaminants remain in the supernatant. The kit also includes proteinase K to degrade potential protein contaminants in the plasma samples. In brief, plasma was clarified by sequential centrifugation and treated with proteinase K. The exosome precipitation reagent was then added, followed by incubation on ice and low‐speed centrifugation to pellet the sEVs. The final pellet was resuspended in PBS for downstream applications. Vesicles were characterized using three methods: nanoparticle tracking analysis (NTA), cryogenic electron microscopy (Cryo‐EM), and immunoblotting.

### 2.6. NTA

Exosome quantification was performed using NTA on the NanoSight NS300 (Malvern Panalytical, NTA software Version 3.4) at the SickKids SPARC facility. Five isolated sEV samples were resuspended in 200 *μ*L of PBS, diluted 1:100, and analyzed at a constant temperature of 25°C. To validate instrument performance, 100‐nm polystyrene latex beads were used as standards prior to each run.

NTA combines laser light scattering microscopy with particle tracking to determine both size distribution and concentration of vesicles. The system tracks the Brownian motion of each particle and using the Stokes–Einstein equation, calculates the hydrodynamic diameter. NTA typically detects particles in the 30‐nm to 2‐*μ*m range. By factoring in the dilution and volume of the sample analyzed, the software also estimates the concentration of vesicles in particles/milliliter.

This method provides a robust quantitative and size distribution profile of sEVs in plasma samples, supporting downstream analysis and validation.

### 2.7. Cryo‐EM

Cryo‐EM was performed at the Princess Margaret Cancer Research Institute (Toronto, Ontario) using sEV samples isolated with the Exosome Isolation Kit (from plasma) from Invitrogen and resuspended in 50 *μ*L of PBS. Three types of grids were tested: holey carbon, holey gold (both subjected to 15 mA, 30‐s glow discharge), and holey gold grids with amylamine added during glow discharge. For each condition, a 3‐*μ*L aliquot of sEV sample was applied to each grid, blotted for 3.5 s at 90% humidity with a 10‐s wait time, and a + 8 blot force, then plunged into liquid ethane using a Vitrobot Mark IV (Thermo Scientific). Cryo‐grids were imaged on a Talos L120C transmission electron microscope (Thermo Scientific) equipped with a Ceta‐M camera and Gatan 626 cryo‐holder (Gatan).

This approach allowed high‐fidelity visualization of vesicle structure while enabling evaluation of grid performance for optimal imaging conditions.

### 2.8. Small RNA Extraction From LDLT Patient Plasma Samples

Small RNAs were extracted from sEVs using Invitrogen′s Total Exosome RNA and Protein Isolation Kit (Thermo Scientific Cat. No. 4478545), following the manufacturer′s small RNA enrichment protocol. This kit was chosen for its compatibility with the previously used Exosome Isolation Kit (from plasma) and its ability to facilitate downstream analysis of RNA from sEVs. Due to the limited input volume (1 mL of plasma per sample), only RNA extractions were performed.

To begin, sEV pellets were resuspended in 200 *μ*L of ice‐cold PBS, incubated at room temperature for 5 min, and gently pipetted to dissolve the pellet. Then, 200 *μ*L of 2X denaturing solution was added, followed by another 5 min incubation. Under a fume hood, 400 *μ*L of Acid–Phenol:Chloroform was added. The mixture was vortexed for 30 s and centrifuged at 21,000 × *g* for 5 min. The upper aqueous phase containing RNA was carefully transferred to a new tube, mixed with 1.25 volumes of 100% ethanol, and loaded onto a filter cartridge. The cartridge was centrifuged at 10,000 × *g* for 30 s to bind the RNA. Wash steps are as follows: first with 700 *μ*L of Wash Solution 1, then twice with 500 *μ*L of Wash Solution 2/3, discarding flow‐through after each wash. A final spin at 21,000 × *g* for 1 min ensured the removal of residual liquid. RNA was then eluted using 50 *μ*L of 95°C elution solution, centrifuged for 30 s, and immediately placed on ice. RNA concentration was measured using the Nanodrop ND‐1000 spectrophotometer.

### 2.9. Cell Culture

HepG2 cells (human hepatocellular carcinoma) were cultured in Dulbecco′s modified Eagle medium (DMEM) (Thermo Scientific, Cat. No. 11885084) supplemented with 10% fetal bovine serum (FBS) (Sigma‐Aldrich, Cat. No. F1051‐500ML). MDA‐MB‐231 cells stably expressing Wnt‐cluster of differentiation 81 (CD81) were maintained in Roswell Park Memorial Institute (RPMI) medium (Gibco, Cat. No. 11875093) with 5% FBS (Sigma‐Aldrich, Cat. No. F1051‐500ML). All cell lines were incubated at 37°C in a humidified atmosphere containing 5% CO_2_. HepG2 whole cell lysate was used as a positive control for general protein markers, whereas Wnt‐CD81 MDA‐MB‐231 lysate served as a control for CD81‐specific detection.

### 2.10. Immunoblotting

Four sEV samples were pooled and resuspended in 200 *μ*L of 5X RIPA buffer (composition: 50 mM Tris‐Cl pH 7.4, 500 mM NaCl, 0.5% sodium deoxycholate, 0.5% SDS, and 1% Triton X‐100), following the protocol adapted from Brennan et al. [8] for extraction to enhance the final protein concentration. Trichloroacetic acid (TCA) precipitation was performed to concentrate the proteins, and the pellets were resuspended in 100 *μ*L of 5X RIPA buffer. Protein concentrations were quantified using the Bradford assay with BSA standards (Bio‐Rad, Cat. No. 5000006). Based on optimization, 30 *μ*g of sEV protein, 100 *μ*g of HepG2 whole cell lysate, and 10 *μ*L of MDA‐MB‐231 Wnt‐CD81 lysate were loaded per lane on 12% SDS‐polyacrylamide gels for protein separation. HepG2 cells (human hepatocellular carcinoma) served as a positive control for general cellular protein markers (e.g., glyceraldehyde 3 phosphate dehydrogenase (GAPDH) and tumor susceptibility gene 101 protein (TSG101)), whereas MDA‐MB‐231 cells stably expressing Wnt‐CD81 were used as a control for CD81 antibody validation.

Proteins were separated by SDS‐PAGE and transferred onto PVDF membranes using the Bio‐Rad PowerPac HC system (Cat. No. 1645052). Membrane transfer was confirmed using Ponceau S staining (Fluka Analytical, Cat. No. 81460). Membranes were blocked for 1 h at room temperature with 5% BSA in PBS‐Tween 20 (0.1%), followed by an overnight incubation at 4°C with primary antibodies. After washing with TBS‐Tween 20 (0.1%), membranes were incubated with HRP‐conjugated secondary antibodies, washed again, and developed using the Bio‐Rad ChemiDoc Imaging System (Cat. No. 12003153).

**Primary antibodies** (Table 1):

**Table 1:** Antibodies employed in western blot analysis.

**Primary antibodies**					
**Antigen**	**Manufacturer**	**Cat. no.**	**Host**	**Specificity**	**Dilution used**
CD81	Santa Cruz	SC‐166029	Mouse	Mouse, rat, and human	1:200
TSG101	Novus Biologicals	NB200‐112	Mouse	Human, mouse, rat, porcine, canine, hamster, and primate	1:1000
Beta‐actin–HRP	Sigma‐Aldrich	A3854	Mouse	Sheep, carp, feline, chicken, rat, mouse, *Hirudo medicinalis*, rabbit, canine, pig, human, bovine, and guinea pig	1:20,000
GAPDH	Sigma‐Aldrich	G9545	Rabbit	Mouse, rat, and human	1:10,000
**Secondary antibodies**					
**Conjugate**	**Manufacturer**		**Host**	**Specificity**	**Dilution used**

HRP	GE healthcare		Sheep and mouse	Mouse	1:10,000
HRP	Santa Cruz		Mouse	Rabbit	1:10,000

• Mouse anti‐CD81 (Santa Cruz, sc‐166029; 1:200)

• Mouse anti‐TSG101 (Novus Biologicals, NB200‐112; 1:1000)

• Mouse anti‐*β*‐actin‐peroxidase (Sigma‐Aldrich, A3854; 1:20,000)

• Rabbit anti‐GAPDH (Sigma‐Aldrich, G9545; 1:10,000)

**Secondary antibodies** (Table 1):

• HRP‐conjugated anti‐mouse (GE Healthcare; 1:10,000)

• HRP‐conjugated anti‐rabbit (Santa Cruz; 1:10,000).

### 2.11. miRNA Expression Assay

miRNA analysis was conducted using the NanoString Technologies′ nCounter Human v3 miRNA Expression Assay Kit (https://nanostring.com/, Seattle, Washington) at the Princess Margaret Cancer Genomics Center (https://www.pmgenomics.ca/pmgenomics/, Toronto, Ontario). This panel evaluates 827 human miRNAs, five mRNAs, and 25 internal reference controls. Each cartridge accommodates up to 12 samples; therefore, 24 samples (12 from each time point) with the highest RNA concentration and optimal curve profiles were selected for analysis.

For human plasma, 24 samples were selected, consisting of 12 samples from each time point. Similarly, a total of 12 murine samples, 6 per time point, were selected based on the RNA yield and curve quality. Murine miRNA profiling was conducted using NanoString′s nCounter Mouse v1.5 miRNA Assay, which includes 577 mouse miRNAs, 33 murine‐associated viral miRNAs, four mRNA probes, and 23 internal reference controls.

Following hybridization and cartridge scanning, raw data were analyzed using nSolver Analysis Software 4.0 (NanoString Technologies). Target miRNAs were normalized against internal positive controls, and normalized miRNA expression levels were calculated as fold changes (FCs) between regenerating and nonregenerating groups. The software applies a two‐tailed *t*‐test to log‐transformed normalization data, generating *p* values to assess the statistical significance of FCs. Only miRNAs with a nonadjusted *p* value < 0.05 and FC < −1.5 or > 1.5 were selected for further analysis.

### 2.12. Murine Transcriptomic Data

To validate human NanoString data, prior transcriptomic data from regenerating mouse liver samples were utilized. Whole mouse liver samples were snap‐frozen in liquid nitrogen at Days 2 and 7 post‐PH. Total RNA was extracted from control, resected, and regenerating liver tissues using Qiagen′s RNeasy kit (Cat. No. 74104), following the manufacturer′s protocols. RNA quality and quantity were assessed with a NanoDrop ND‐1000 UV‐Vis Spectrophotometer.

Gene expression microarray was analyzed using the Affymetrix Mouse Gene 2.0 ST Array (Thermo Scientific), processed by The Centre for Applied Genomics (TCAG) facility at SickKids (Toronto, Ontario). Transcriptome Analysis Console (TAC) software (Thermo Scientific) was employed for initial data preprocessing, including background subtraction and quantile normalization, which also facilitated gene annotation, statistical analysis, and differential gene expression identification. Genes with a false discovery rate (FDR)‐adjusted *p* value < 0.05 and FC < −1.5 or > 1.5 were considered differentially expressed and significant.

After eliminating duplicate genes between the groups, our master list comprised 3019 significant genes. Specifically, this included 1336 genes from male mice at 2 days post‐PH, 2389 genes from female mice at 2 days post‐PH, 100 genes from male mice at 7 days post‐PH, and 14 genes from female mice at 7 days post‐PH. Given that human plasma samples were not sex‐differentiated, data from male and female mouse samples were pooled at both time points and filtered based on their direction of FC (up or downregulated). At 2 days post‐PH, 1828 genes were found to be upregulated, whereas at 7 days post‐PH, 119 genes were downregulated.

### 2.13. miRNA Data Integration Portal (mirDIP)

To identify potential gene targets of differentially expressed miRNAs, we used the mirDIP, which aggregates over 152 million human miRNA–target interaction predictions [9]. To ensure high reliability, mirDIP includes only predictions supported by at least four independent algorithms with high confidence scores. Additionally, mirDIP provides a tissue search function, enabling users to identify the tissue origin of specific miRNAs. Version 5.2 of mirDIP integrates data from 20 sources covering 330 different tissue and disease contexts [10].

For this study, mirDIP Version 5.3.0.1 (https://ophid.utoronto.ca/mirDIP/) was used to predict putative gene targets of significant miRNAs (*p* value < 0.05 and FC < −1.5 or > 1.5). Only genes within the Top 1% score class were considered for further analysis. Additionally, the tissue search function was used to identify miRNAs associated with cirrhotic liver tissues. This enabled further contextualization of circulating miRNA signals within the biological framework of liver disease.

### 2.14. STRING (Search Tool for the Retrieval of Interacting Genes/Protein)

Pathway analysis of our predicted gene targets was performed using STRING Version 12 (https://string-db.org/), a comprehensive database of known and predicted protein–protein interactions [11]. STRING consolidates interactions from computational predictions, experimental data, and knowledge transfer across organisms, covering both physical (direct) and functional (indirect) associations. To assess the statistical enrichment of functional categories among protein sets, STRING applies Fisher′s exact test to determine whether the observed overlap is significant. To control for false positives arising from multiple comparisons, correction methods such as the Benjamini–Hochberg procedure are employed. As of Version 12, the STRING database currently includes 59,309,604 proteins from 12,535 organisms [11], making it one of the most extensive resources for functional interaction networks.

Genes from mirDIP were input into STRING v12 to determine their interactions and pathways′ involvements. Only pathways with the highest confidence in interactions were included, and disconnected nodes were excluded from the analysis. This approach enabled visualization of key molecular pathways potentially regulated by the miRNAs identified in our dataset.

## 3. Results

### 3.1. Techniques Used for Exosome Characterization

#### 3.1.1. NTA Categorizes Vesicles as sEVs

We employed NTA to determine the average size and concentration of our sEVs. NTA results (Figure 1a) showed that vesicles that were isolated from 5 mL of patient plasma samples had a mean diameter of 178.6 nm (179.6, 199, 150.8, 182.1, and 181.6), indicating that they are greater than the 30–150‐nm range of exosomes but under the 200‐nm limit of sEVs [12]. With this information, we proceeded to classify our EVs as sEVs.

Figure 1Exosome characterization by NTA, Cryo‐EM, and immunoblotting analysis of exosome‐enriched protein markers. (a) NTA plot for sEVs in a plasma sample, samples were diluted 1:100 for NTA. Mean diameter is greater than 150 nm and less than 200 nm, suggesting that vesicles should be classified as small extracellular vesicles (sEVs). (b) Cryo‐EM image of sEVs (arrows) from human plasma. Scale bar is 200 nm. (c) CD81 exosome‐enriched protein marker. (d) TSG101 exosome‐enriched protein marker. (e) Beta‐actin: negative protein marker. (f) GAPDH: negative protein marker.

**Figure:**
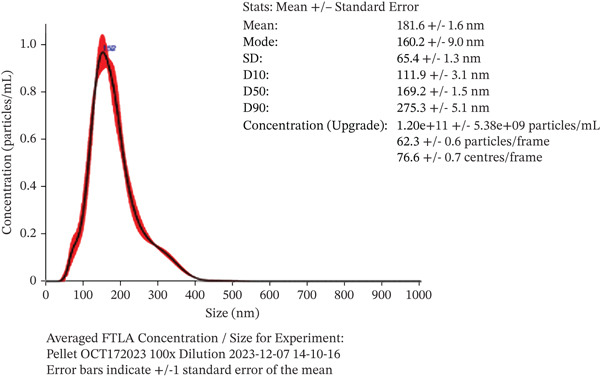
(a)

**Figure:**
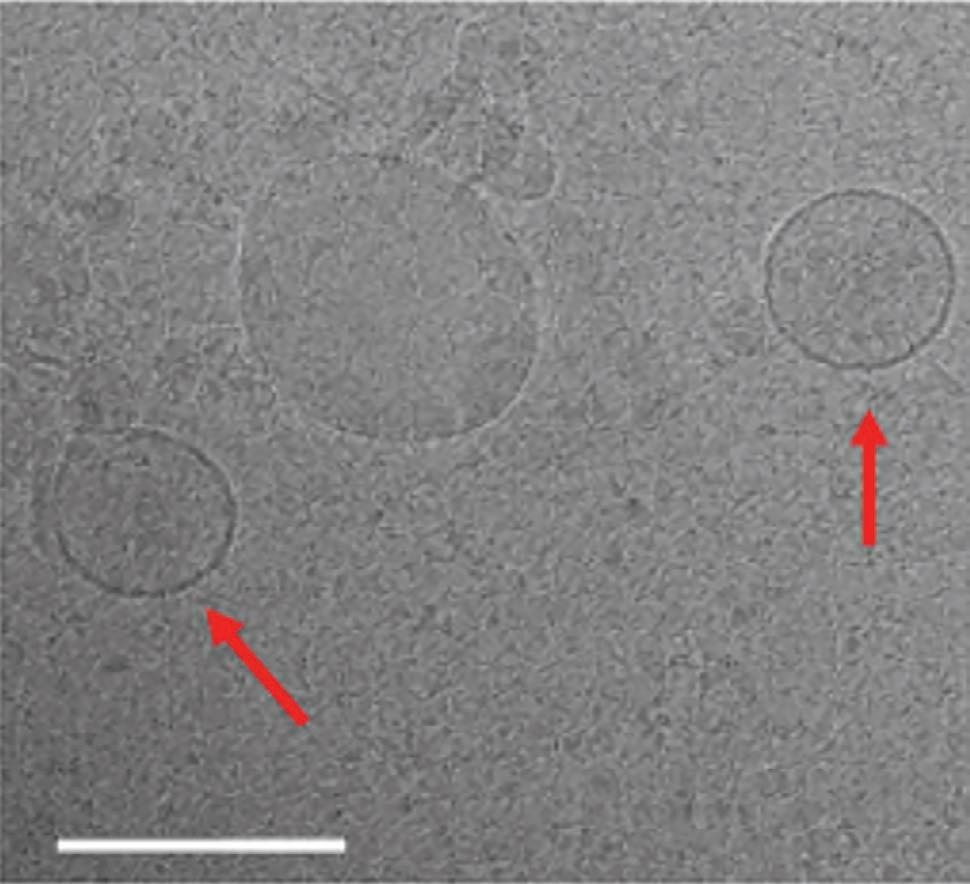
(b)

**Figure:**
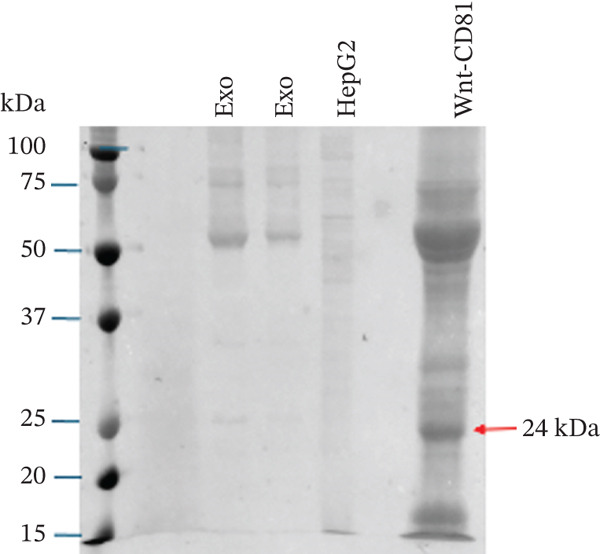
(c)

**Figure:**
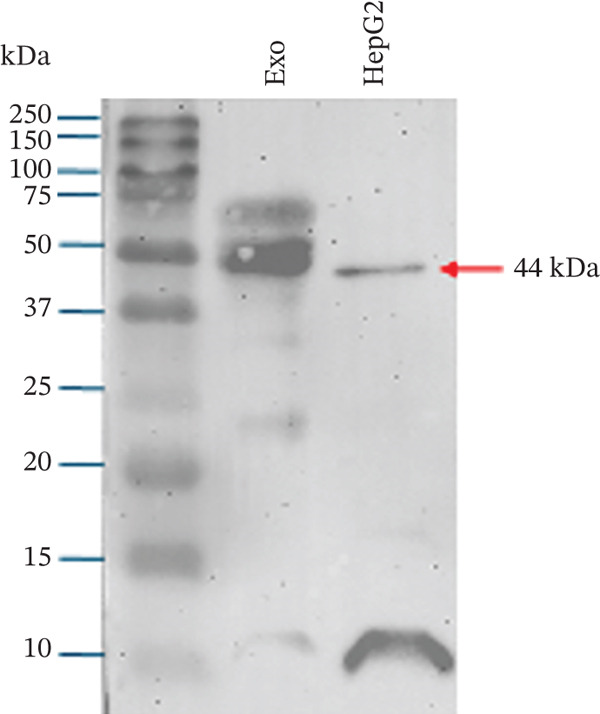
(d)

**Figure:**
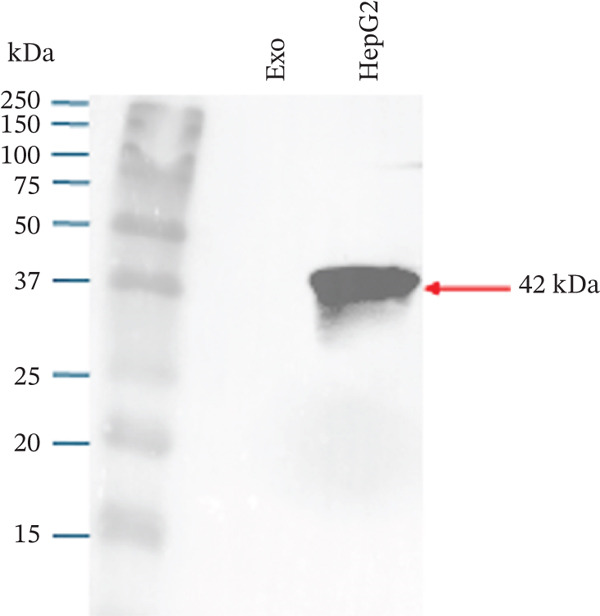
(e)

**Figure:**
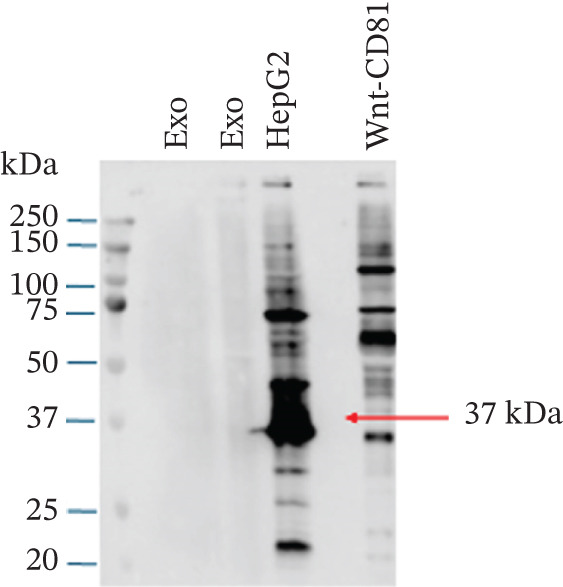
(f)

#### 3.1.2. Cryo‐EM of sEVs

To further characterize our sEVs, we used Cryo‐EM. This allowed us to obtain detailed images of vesicle morphology and structural features. The most commonly observed vesicles were enclosed by a single lipid bilayer (Figure 1b). These vesicles appeared as spherical structures with diameters ranging from 30 to 150 nm, consistent with the size range of typical sEVs.

#### 3.1.3. Western Blotting Shows Exosome Enriched Proteins CD81 and TSG101

The last method used to characterize our sEVs was immunoblotting to detect exosome‐enriched proteins, following the “Minimal Information for Studies of Extracellular Vesicles” (MISEV) guidelines. Although EVs are heterogeneous, certain proteins, such as the tetraspanins CD9, CD63, and CD81, and cytosolic proteins like TSG101, are enriched in exosomes and commonly used as markers [13, 14] (Figures 1c, 1d, 1e, and 1f).

In our study, we analyzed two positive markers: CD81, a tetraspanin involved in membrane fusion and cell signaling, and TSG101, a cytosolic protein essential for exosome biogenesis through its role in the ESCRT pathway (Figure 1c,d). To ensure specificity, we included beta‐actin and GAPDH as negative controls (Figure 1e,f). These cellular proteins are not typically enriched in exosomes and have been shown by Jeppesen et al. [15] to associate with nonvesicular fractions.

Immunoblotting successfully detected CD81 and TSG101 in our sEV preparations, confirming the enrichment of exosome‐specific markers. As positive controls, we used a Wnt‐CD81 cell lysate for CD81 and a HepG2 cell lysate for TSG101 [16]. Neither beta‐actin, nor GAPDH were detected in the exosome lanes, further supporting the purity of our exosome preparations.

#### 3.1.4. Human miRNA Analysis

Analysis of raw data from NanoString′s nSolver software found 798 miRNAs in the sEVs isolated from patient plasma (Table S2). Among these, 25 miRNAs met the criteria for statistical significance, with a nonadjusted *p* value < 0.05 and FC < −1.5 or > 1.5 (Table 2). Significant miRNAs were input into mirDIP, which predicted 3347 putative target genes in the Top 1%, confidence class, indicating a strong likelihood of interaction with the identified miRNAs (Table S3).

**Table 2:** Key microRNAs identified in human NanoString analysis.

MicroRNA	Fold change	*p*
*hsa-miR-1275*	−1.78	0.00850167
*hsa-miR-660-5p*	−1.55	0.01298199
*hsa-miR-450b-5p*	−1.53	0.01290884
*hsa-miR-942-3p*	−1.48	0.01962815
*hsa-miR-620*	−1.47	0.00802995
*hsa-miR-1303*	−1.46	0.00922744
*hsa-miR-523-3p*	−1.45	0.02389728
*hsa-miR-548b-3p*	−1.45	0.04436299
*hsa-miR-219a-1-3p*	−1.41	0.01893805
*hsa-miR-125a-5p*	−1.37	0.01269488
*hsa-miR-502-3p*	−1.37	0.01377925
*hsa-miR-22-3p*	−1.35	0.02581726
*hsa-miR-875-3p*	−1.33	0.00848867
*hsa-miR-1258*	−1.33	0.01460284
*hsa-miR-892a*	−1.28	0.04646643
*hsa-miR-28-3p*	−1.26	0.0340091
*hsa-miR-1255a*	−1.26	0.04767832
*hsa-miR-4286*	−1.25	0.02546456
*hsa-miR-433-5p*	−1.25	0.03436048
*hsa-miR-99b-5p*	−1.24	0.03279238
*hsa-miR-125a-3p*	−1.24	0.04262123
*hsa-miR-1278*	−1.23	0.02754533
*hsa-miR-197-3p*	−1.22	0.03541298
*hsa-miR-615-3p*	−1.2	0.04922752
*hsa-miR-328-5p*	−1.19	0.03790019

#### 3.1.5. Human miRNA Target Genes Overlap With Hippo and Cell Cycle Genes

Leveraging gene lists from the Reactome database, we identified substantial overlaps between our predicted miRNA target genes and those involved in these pathways. Notably, approximately half of the genes in the Hippo pathway and over 20% in the cell cycle pathway were identified as miRNA targets (Figure 2a,b; Table S4).

Figure 2Putative human target genes and the different regenerative pathways that are impacted. (a) Venn diagram shows 10 genes in the Hippo pathway putatively targeted by human miRNAs during liver regeneration. (b) Venn diagram shows 143 genes in the cell cycle pathway putatively targeted by human miRNAs during liver regeneration. (c) STRING pathway analysis of cell cycle genes targeted by human miRNAs in regenerating liver. Notable pathways include cell cycle (red), DNA replication (purple), Hedgehog signaling (green), p53 signaling (dark pink), Hippo (light blue), TGF‐*β* signaling (gray), hepatocellular carcinoma (dark green), and PI3K‐Akt signaling (light brown); (d) Number of putative target genes in the cell cycle (orange) and Hippo (blue) pathways impacted by significant human sEV miRNAs.

**Figure:**
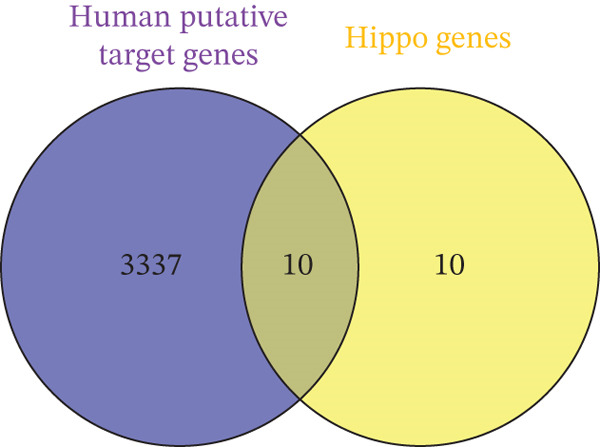
(a)

**Figure:**
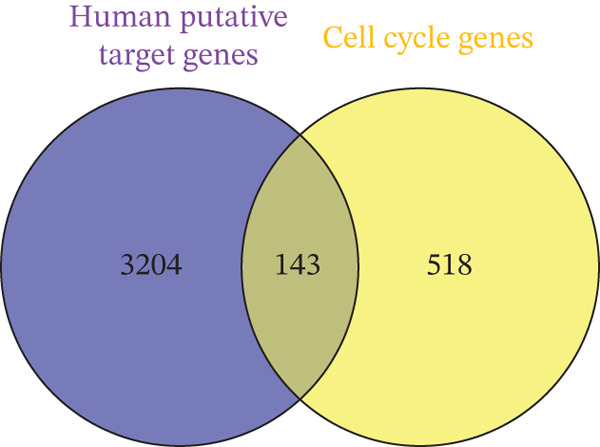
(b)

**Figure:**
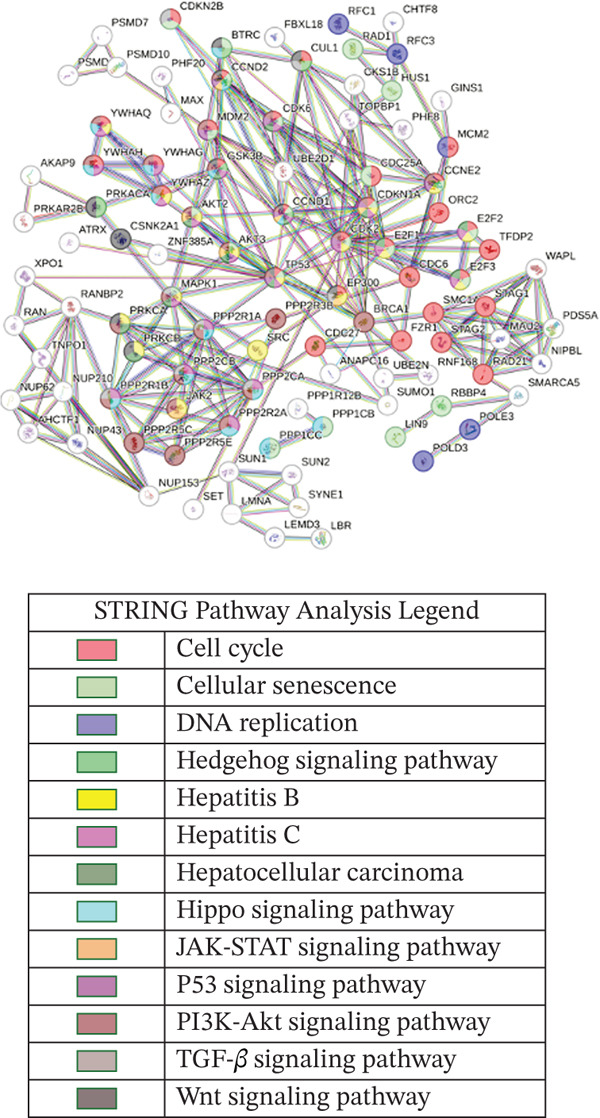
(c)

**Figure:**
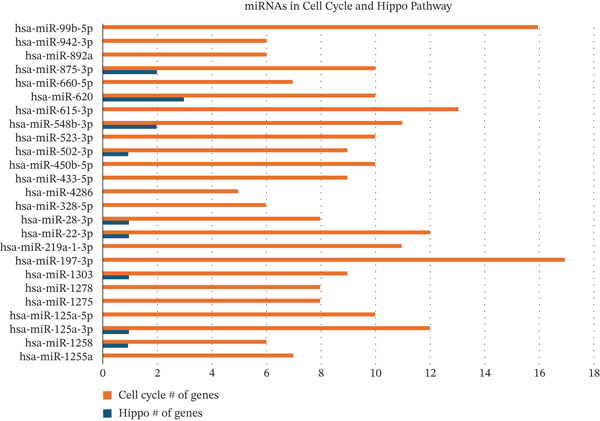
(d)

STRING database analysis further confirmed that these overlapping genes participate in critical processes, including DNA replication, cellular senescence, and Hippo signaling (Figure 2c and Table 3). Figure 2d illustrates the number of genes that each significant miRNA impacts within Hippo and cell cycle. For instance, *miR-620* impacts 10 genes in the cell cycle and three in Hippo, whereas *miR-197-3p* impacts 17 genes in the cell cycle pathway (Figure 2d).

**Table 3:** STRING predicted pathways of human putative target genes overlapping with cell cycle genes.

Pathway	FDR
Cell cycle	3.24e − 34
Cellular senescence	1.46e − 20
Hepatitis C	3.87e − 19
PI3K‐Akt signaling pathway	9.38e − 19
Hepatitis B	3.51e − 13
Hippo signaling pathway	2.36e − 12
Hepatocellular carcinoma	4.61e − 09
Wnt signaling pathway	2.94e − 07
P53 signaling pathway	9.64e − 07
TGF‐beta signaling pathway	4.12e − 06
Hedgehog signaling pathway	1.22e − 05
DNA replication	5.97e − 05
JAK‐STAT signaling pathway	8.5e − 04

#### 3.1.6. Human miRNA Putative Target Genes That Overlap With Upregulated Murine Genes at 2 Days Post‐PH Are Involved in Cell Cycle Transitioning

We identified several human miRNA putative target genes, which overlap with murine genes upregulated at 2 days post‐PH, play critical roles in cell cycle transitioning (Figure 3a and Table S5). Key genes identified in this overlap include Cyclin D1 (*CCND1*), breast cancer Type 1 susceptibility protein (*BRCA1*), E2F transcription Factor 1 (*E2F1*), and Cyclin E2 (*CCNE2*).

Figure 3Integration of miRNA target genes with cell cycle pathways and differentially expressed genes in mouse liver regeneration post‐PH. (a) miRNA target genes in common with cell cycle genes and upregulated mouse genes at 2 days post‐PH. Genes in common between the groups are involved in processes such as cell cycle transitions and cell proliferation such as *CCND1*, *CCNE2*, and *BRCA1*. (b) miRNA target genes in common with cell cycle genes and mice genes downregulated at 7 days post‐PH. Notable genes and their interactions include those involved in cell death processes such as apoptosis and necroptosis.

**Figure:**
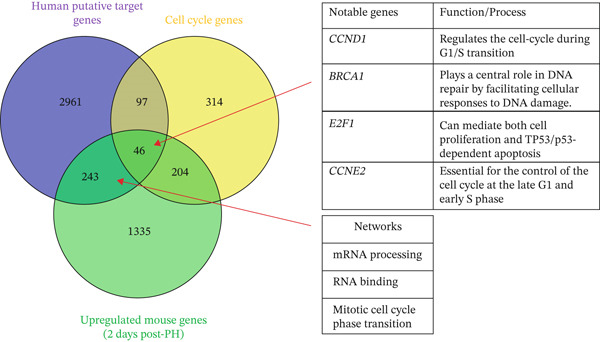
(a)

**Figure:**
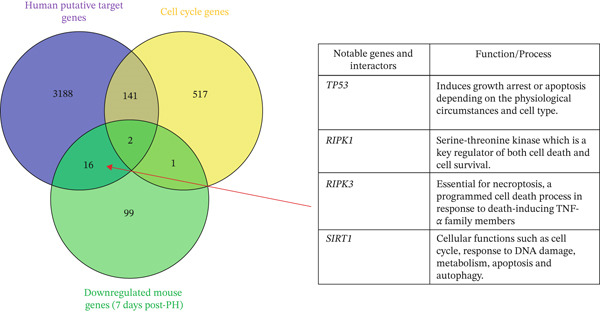
(b)

The convergence of these genes, both as human miRNA targets and as murine genes upregulated post‐PH, highlights their important roles in cell cycle regulation and their potential as therapeutic targets for enhancing liver regeneration. Our findings suggest that these miRNA target genes are crucial for the effective transition through cell cycle phases, ensuring successful liver tissue repair and regeneration following hepatic injury.

#### 3.1.7. Human miRNA Target Gene and Interactors That Overlap With Downregulated Murine Genes at 7 Days Post‐PH Are Involved in Cell Death

Comparative analysis revealed that human miRNA target genes overlapping with murine genes downregulated at 7 days post‐PH are associated with genes involved in apoptosis and necroptosis such as tumor Protein p53 (*TP53*), Sirtuin 1 (*SIRT1*), receptor‐interacting serine/threonine‐protein Kinase 1 (*RIPK1*), and receptor‐interacting serine/threonine‐protein Kinase 3 (*RIPK3*) (Figure 3b and Table S6).

This may be indicative of tight control of cell death signaling during the termination phase of liver regeneration. Remodeling at this stage likely involves balanced regulation of cell survival and clearance pathways to ensure proper tissue architecture and restoration of liver function. These findings reflect the importance of the modulation of apoptosis and necroptosis pathways and suggest potential therapeutic targets to optimize liver recovery.

#### 3.1.8. Tissue Source of Human miRNAs

Using the tissue search function in mirDIP, we identified that 64% of significant miRNAs in our plasma samples are present in cirrhotic liver tissue (Table S7). Liver cirrhosis was used as a model in this analysis, as it is the closest tissue or disease context available in mirDIP, with the source of this prediction being Panwar et al. [17]. Liver cirrhosis is a pathological condition characterized by the replacement of healthy liver tissue with scar tissue/fibrosis due to improper cell regeneration after damage.

Of the 25 significant miRNAs, 16 were associated with liver cirrhosis, including *hsa-miR-1258*, *hsa-miR-125a-3p*, and *hsa-miR-125a-5p*, among others. These findings emphasize their potential roles in liver regeneration and warrant further investigation.

#### 3.1.9. Tissue Source of Murine miRNAs

Similarly, mirDIP predicted that 67% of murine miRNAs are linked to liver cirrhosis (Table S8). Significant miRNAs included *hsa-miR-125a-3p*, *hsa-miR-133b*, and *hsa-miR-146b-5p*, among others. *hsa-miR-101-2-5p* was not identified. These results suggest potential roles for these miRNAs in liver function, highlighting the need for further study.

### 3.2. Murine miRNA Analysis

#### 3.2.1. Microarray Analysis

After removing duplicate genes across groups, the final dataset included 3019 significant genes: 1336 genes from male mice at 2 days post‐PH, 2389 from female mice at 2 days post‐PH, 100 from male mice at 7 days post‐PH, and 14 from female mice at 7 days post‐PH. Since human plasma samples were not sex‐differentiated, male and female mouse data were pooled for both time points. Genes were further filtered based on FC direction (upregulated or downregulated). At 2 days post‐PH, 1,828 genes were upregulated, whereas 119 were downregulated at 7 days post‐PH.

#### 3.2.2. Nano String Analysis

Analysis of murine plasma sEVs at 2 and 7 days post‐PH identified 596 miRNAs, of which 30 (or 49 when considering both 3p and 5p arms) were significant (*p* value ≤ 0.05, FC < −1.5 or > 1.5; Tables S9 and S10). Since mirDIP supports only human miRNAs, murine miRNAs were converted to human homologs using miRBase and NCBI BLASTn. This process yielded 30 human homologs, 24 of which were verified by mirDIP.

#### 3.2.3. Murine Putative Target Genes Overlap With Cell Cycle and Hippo Pathway

mirDIP predicted 2893 target genes for the 24 verified murine miRNAs (Table S11). Comparison with cell cycle genes revealed key players involved in regeneration (Figure 4a). In the Hippo pathway, notable genes included MOB kinase activator 1A (*Mob1A*) and a regulator of (large tumor Suppressors 1 and 2) *Lats1/2* which are key kinases in the Hippo pathway [18]. Other genes impacted in the Hippo pathway include angiomotin (*Amot*) and Tyrosine 3‐Monooxygenase (*Ywhab*), both of which regulate Yes‐associated protein 1 (*Yap1*), another critical component of the Hippo pathway [19, 20].

Figure 4Comparative analysis of miRNA‐associated genes and pathways in liver regeneration. (a) Venn diagrams comparing genes from human and mouse miRNA. (b) Human putative target genes and human homolog putative target genes. (c) Putative human homolog genes overlap with cell cycle and Hippo genes. (d) STRING network analysis of human, murine, and cell cycle protein interactions. Notable pathways include cell cycle (red), Hippo (pink), Hedgehog (dark green), p53 (light blue), TGF‐*β* (mauve), PI3K‐Akt (brown), and Wnt (gray).

**Figure:**
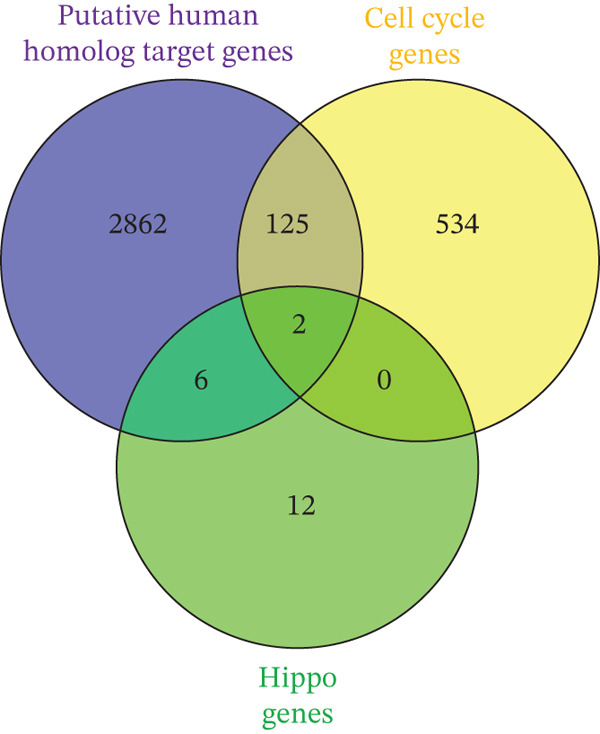
(a)

**Figure:**
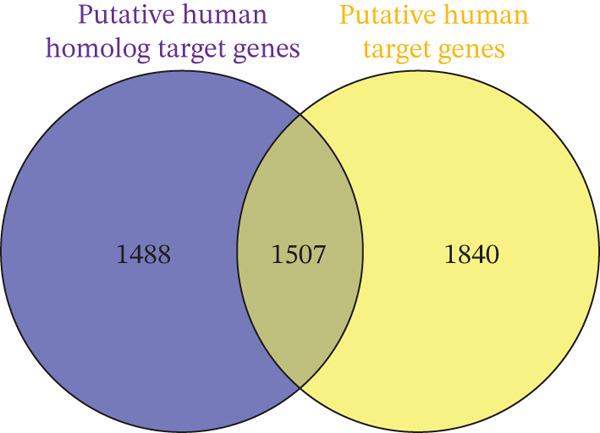
(b)

**Figure:**
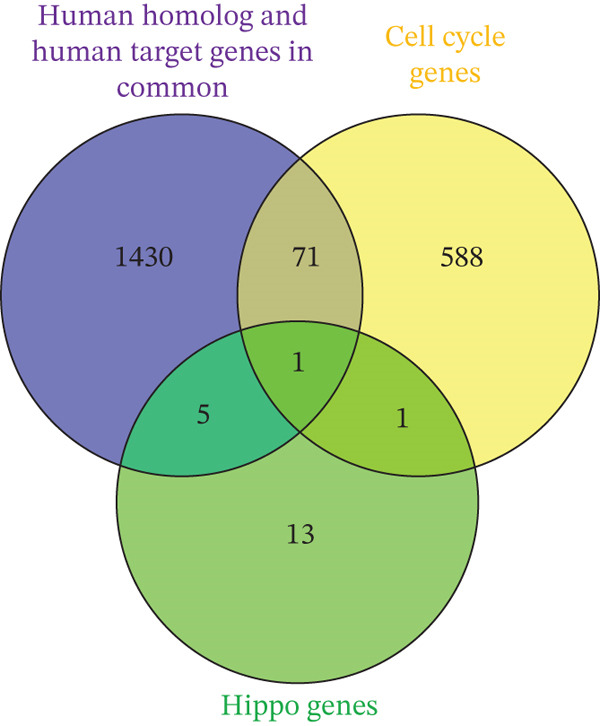
(c)

**Figure:**
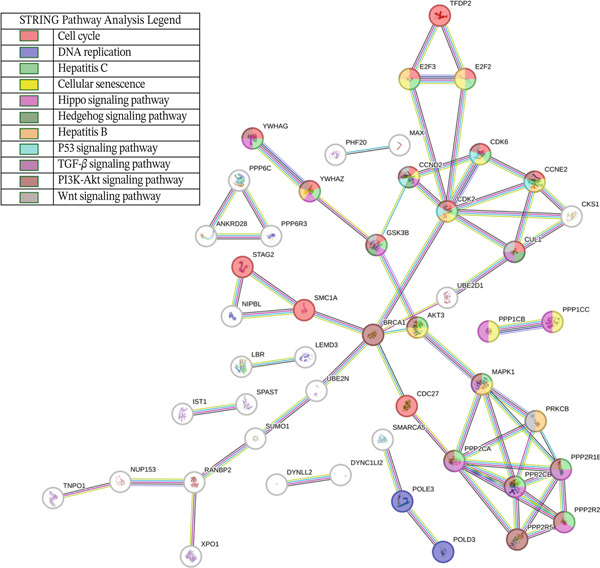
(d)

#### 3.2.4. Putative Genes in Common Between Human and Murine

Comparing significant human miRNAs with murine miRNA homologs revealed two that overlap: *hsa-miR-450b-5p* and *hsa-miR-125a-3p*. Using mirDIP, we found that there are 1507 putative target genes in common between the two miRNAs (Figure 4b). This substantial overlap indicates that over half of the putative murine genes align with putative human genes, suggesting a conserved regulatory role across species.

Next, cross‐referencing with cell cycle and Hippo pathways revealed 71 overlapping genes, mapped to pathways such as Hippo, PI3K‐Akt, Wnt, Hh, and TGF‐*β* signaling pathways (Tables S12 and 4 and Figure 4c,d). STRING analysis suggests that these genes play critical roles in liver regeneration, offering insights into conserved regulatory mechanisms across species (Figure 4d).

**Table 4:** STRING predicted pathways of human putative target genes in common with human homolog putative target genes overlapping with cell cycle genes.

Pathway	FDR
Cell cycle	3.28e − 16
Hepatitis C	3.73e − 12
PI3K‐Akt signaling pathway	1.40e − 11
Cellular senescence	8.36e − 10
Hippo signaling pathway	1.68e − 08
Hepatitis B	2.35e − 07
DNA replication	1.1e − 04
TGF‐beta signaling pathway	2.1e − 04
P53 signaling pathway	1.2e − 03
Wnt signaling pathway	1.8e − 03
Hedgehog signaling pathway	4.3e − 03

## 4. Discussion

The liver′s regenerative ability is well‐documented, but the role of sEVs in human liver regeneration remains unclear.

Our findings emphasize the importance of cell cycle regulation during regeneration as established by prior studies [1, 21, 22]. Genes such as *CCND1*, *BRCA1*, *E2F1*, and *CCNE2*, which facilitate the G1/S transition and DNA replication, were upregulated early post‐PH, supporting efficient tissue repair. Conversely, the downregulation of *TP53* at 7 days post‐PH may reflect resolution of the regenerative response and restoration of homeostatic control, with reduced proliferative signaling. Remodeling at this stage likely involves tightly regulated pathways that balance cell survival and controlled clearance of damaged cells, potentially through SIRT1 mediated regulation of stress responses and context dependent activation of RIPK1/RIPK3 signaling under inflammatory conditions.

Our miRNA profiling identified conserved roles for *hsa-miR-450b-5p* and *hsa-miR-125a-3p* in human and murine liver regeneration. *hsa-miR-450b-5p*, a suppressor of acute liver failure and ischemia‐reperfusion (I/R) injury, regulates *MDM2*, influencing cell cycle and inflammation, whereas *hsa-miR-125a-3p* aids in post‐PH recovery by modulating proliferation and energy production pathways [23–27]. Other miRNAs, such as *miR-192-5p*, linked to I/R protection, and *miR-196* and *miR-31*, involved in tissue repair, also merit further investigation [25, 26, 28–30].

In summary, our findings suggest that sEV‐miRNAs, particularly *hsa-miR-450b-5p* and *hsa-miR-125a-3p*, have potential as biomarkers for liver regeneration. Future studies should validate these biomarkers in larger cohorts and examine their therapeutic potential.

Limitations of our study include the lack of functional validation of identified miRNAs and a small sample size, which may affect statistical power. Employing techniques such as RNA interference or CRISPR‐Cas9 will be crucial for confirming their roles and clinical relevance.

In conclusion, this study enhances our understanding of the molecular mechanisms driving liver regeneration, highlighting sEV‐miRNAs as promising diagnostic and therapeutic tools. These findings pave the way for less invasive diagnostics and improved treatment strategies for post‐liver transplant patients.

**Nomenclature**
ALT: alanine aminotransferase
mirDIP: microRNA Data Integration Portal
AST: aspartate aminotransferase
miRNA: microRNA
AMOT: angiomiotin
NTA: nanoparticle tracking analysis
BRCA1: breast cancer gene 1
PH: partial hepatectomy
CCND1: cyclin D1
PI3‐K: phosphoinositide 3‐kinase
CCNE2: cyclin E2
Akt: protein kinase B
Cryo‐EM: cryogenic electron microscopy
RIPKs: receptor‐interacting protein kinases
DMEM: Dulbecco′s modified Eagle′s medium
RPMI: Roswell Park Memorial Institute
E2F1: E2F transcription factor 1
sEV: small extracellular vesicle
FBS: fetal bovine serum
SIRT1: sirtuin1
FC: fold change
STAT3: signal transducer and activator of transcription 3
FDR: false discovery rate
STRING: Search Tool for the Retrieval of Interacting Genes/Protein
Hh: Hedgehog
TAC: Transcriptome Analysis Console
IL‐6: Interleukin‐6
TCAG: The Centre for Applied Genomics
TGF‐*β*: transforming growth factor beta
I/R: ischemia/reperfusion
TNF: tumor necrosis factor
LATS1/2: large tumor suppressor kinase ½
TP53: tumor protein p53
LDLT: living donor liver transplant
UHN: University Health Network
MOT: Multi‐Organ Transplant
TCA: Trichloroacetic acid
MISEV: Minimal Information for Studies of Extracellular Vesicles
GAPDH: Glyceraldehyde 3 phosphate dehydrogenase
TSG101: Tumor susceptibility gene 101
CD81: Cluster of differentiation 81
I/R: Ischemia reperfusion
MOB1A: MOB kinase activator 1A
YAP1: yes‐associated protein
MOT: multiorgan transplant
YWHAB: tyrosine 3‐monooxygenase

## Author Contributions

Yilin Sun: methodology, investigation (experiments and data collection), visualization (data presentation), and writing—original draft. Elisa Pasini: methodology, investigation (experiments and data collection), visualization (data presentation), project administration, and writing—review and editing. Anh Thu Nguyen‐Lefebvre: methodology and investigation (experiments and data collection). Mamatha Bhat: conceptualization (overall research goals and aims), funding acquisition, supervision, and writing—review and editing.

## Funding

This study was supported by the Ontario Graduate Scholarship, the Natural Sciences and Engineering Research Council of Canada, and the University Health Network Foundation.

## Disclosure

This manuscript is based on research previously presented as part of a thesis available at: https://www.proquest.com/docview/3128000036.

## Ethics Statement

All the research was conducted in accordance with both the Declarations of Helsinki and Istanbul. Our study complied with all ethical regulations. Patients provided written consent. The Research Ethics Board at the University Health Network (UHN) approved this study (REB# 17‐5311).

## Conflicts of Interest

The authors declare no conflicts of interest.

## Supporting information


**Supporting Information** Additional supporting information can be found online in the Supporting Information section. Table S1: Patient demographics for sample collection. Table S2: List of all human miRNA found in circulating small EVs from NanoString. Table S3: List of putative human target genes obtained from mirDIP. Table S4: List of human genes input into STRING. Table S5: List of genes in common between human putative target genes, upregulated mouse genes at 2 days post‐PH, and cell cycle genes. Table S6: List of genes in common between human putative target genes, downregulated mouse genes at 7 days post‐PH, and cell cycle genes. Table S7: Putative tissue source from human plasma microRNA. Table S8: Putative tissue source of murine microRNAs. Table S9: Key microRNAs identified in murine NanoString analysis. Table S10: List of all murine miRNA found in circulating small EVs from NanoString. Table S11: List of putative murine target genes obtained from mirDIP. Table S12: Genes input into STRING database.

## Data Availability

The data that supports the findings of this study are available in the supporting information of this article.
