# Supplementary material for: Understanding the Mechanisms of Human Liver Regeneration via Characterization of Circulating Extracellular Vesicles
Source: Biomed Res Int. 2026 Mar 8;2026:3824534. doi: 10.1155/bmri/3824534 (PMC12968334; doi:10.1155/bmri/3824534)
Supplement: Supplementary file 1 — Supporting Information Additional supporting information can be found online in the Supporting Information section. Table S1: Patient demographics for sample collection. Table S2: List of all human miRNA found in circulating small EVs from NanoString. Table S3: List of putative human target genes obtained from mirDIP. Table S4: List of human genes input into STRING. Table S5: List of genes in common between human putative target genes, upregulated mouse genes at 2 days post‐PH, and cell cycle genes. Table S6: List of genes in common between human putative target genes, downregulated mouse genes at 7 days post‐PH, and cell cycle genes. Table S7: Putative tissue source from human plasma microRNA. Table S8: Putative tissue source of murine microRNAs. Table S9: Key microRNAs identified in murine NanoString analysis. Table S10: List of all murine miRNA found in circulating small EVs from NanoString. Table S11: List of putative murine target genes obtained from mirDIP. Table S12: Genes input into STRING database. [file BMRI-2026-3824534-s001.docx]

Supporting Information Tables (STables)

STable 1: Patient Demographics for Sample Collection

| Patient Demographics | |
| --- | --- |
| Mean age at time of transplant (range) | 61 (24-68) |
| Gender, No. of men (%) | 6 (50%) |
| Etiology   - Hepatitis C - Alcoholic cirrhosis - MASH - Primary sclerosing cholangitis - Primary biliary cholangitis | 2 (15%)  5 (38%)  3 (23%)  2 (15%)  1 (8%) |
| Diabetes | 4 (33%) |
| Immunosuppressive regimen   - Prednisone (5-60 mg) - Tacrolimus (0.5-10 mg) - Mycophenolate mofetil (1-1.5 g) - Cyclosporine | 13  12  13  1 |

STable 2: List of All Human MiRNA Found in Circulating Small EVs from NanoString

| 798 Significant MiRNAs Found in Human Small EVs | | | | |
| --- | --- | --- | --- | --- |
| *hsa-let-7a-5p* | *hsa-miR-1537-3p* | *hsa-miR-3180-5p* | *hsa-miR-563* | *hsa-miR-4741* |
| *hsa-let-7b-5p* | *hsa-miR-154-5p* | *hsa-miR-3182* | *hsa-miR-564* | *hsa-miR-4755-5p* |
| *hsa-let-7c-5p* | *hsa-miR-155-5p* | *hsa-miR-3185* | *hsa-miR-566* | *hsa-miR-4787-3p* |
| *hsa-let-7d-5p* | *hsa-miR-15a-5p* | *hsa-miR-3190-3p* | *hsa-miR-567* | *hsa-miR-4787-5p* |
| *hsa-let-7e-5p* | *hsa-miR-15b-5p* | *hsa-miR-3192-5p* | *hsa-miR-568* | *hsa-miR-4792* |
| *hsa-let-7f-5p* | *hsa-miR-16-5p* | *hsa-miR-3195* | *hsa-miR-570-3p* | *hsa-miR-483-3p* |
| *hsa-let-7g-5p* | *hsa-miR-181a-2-3p* | *hsa-miR-3196* | *hsa-miR-571* | *hsa-miR-483-5p* |
| *hsa-let-7i-5p* | *hsa-miR-181a-3p* | *hsa-miR-32-5p* | *hsa-miR-572* | *hsa-miR-484* |
| *hsa-miR-1-3p* | *hsa-miR-181a-5p* | *hsa-miR-3202* | *hsa-miR-573* | *hsa-miR-485-3p* |
| *hsa-miR-1-5p* | *hsa-miR-181b-2-3p* | *hsa-miR-320a* | *hsa-miR-574-3p* | *hsa-miR-485-5p* |
| *hsa-miR-100-5p* | *hsa-miR-181b-5p+hsa-miR-181d-5p* | *hsa-miR-320b* | *hsa-miR-574-5p* | *hsa-miR-486-3p* |
| *hsa-miR-101-3p* | *hsa-miR-181c-5p* | *hsa-miR-320c* | *hsa-miR-575* | *hsa-miR-487a-3p* |
| *hsa-miR-103a-3p* | *hsa-miR-181d-3p* | *hsa-miR-320d* | *hsa-miR-576-3p* | *hsa-miR-487b-3p* |
| *hsa-miR-105-5p* | *hsa-miR-182-3p* | *hsa-miR-320e* | *hsa-miR-576-5p* | *hsa-miR-487b-5p* |
| *hsa-miR-106a-5p+hsa-miR-17-5p* | *hsa-miR-182-5p* | *hsa-miR-323a-3p* | *hsa-miR-577* | *hsa-miR-488-3p* |
| *hsa-miR-106b-5p* | *hsa-miR-1827* | *hsa-miR-323a-5p* | *hsa-miR-578* | *hsa-miR-489-3p* |
| *hsa-miR-107* | *hsa-miR-183-5p* | *hsa-miR-323b-3p* | *hsa-miR-579-3p* | *hsa-miR-490-3p* |
| *hsa-miR-10a-5p* | *hsa-miR-184* | *hsa-miR-323b-5p* | *hsa-miR-579-5p* | *hsa-miR-490-5p* |
| *hsa-miR-10b-5p* | *hsa-miR-185-5p* | *hsa-miR-324-3p* | *hsa-miR-580-3p* | *hsa-miR-491-3p* |
| *hsa-miR-1178-3p* | *hsa-miR-186-5p* | *hsa-miR-324-5p* | *hsa-miR-582-3p* | *hsa-miR-491-5p* |
| *hsa-miR-1180-3p* | *hsa-miR-187-3p* | *hsa-miR-325* | *hsa-miR-582-5p* | *hsa-miR-492* |
| *hsa-miR-1183* | *hsa-miR-188-3p* | *hsa-miR-326* | *hsa-miR-584-3p* | *hsa-miR-493-3p* |
| *hsa-miR-1185-1-3p* | *hsa-miR-188-5p* | *hsa-miR-328-3p* | *hsa-miR-584-5p* | *hsa-miR-494-3p* |
| *hsa-miR-1185-2-3p* | *hsa-miR-18a-5p* | *hsa-miR-328-5p* | *hsa-miR-585-3p* | *hsa-miR-494-5p* |
| *hsa-miR-1185-5p* | *hsa-miR-18b-5p* | *hsa-miR-329-3p* | *hsa-miR-587* | *hsa-miR-495-3p* |
| *hsa-miR-1193* | *hsa-miR-1908-3p* | *hsa-miR-329-5p* | *hsa-miR-589-5p* | *hsa-miR-495-5p* |
| *hsa-miR-1197* | *hsa-miR-1908-5p* | *hsa-miR-330-3p* | *hsa-miR-590-3p* | *hsa-miR-496* |
| *hsa-miR-1200* | *hsa-miR-1909-3p* | *hsa-miR-330-5p* | *hsa-miR-590-5p* | *hsa-miR-497-5p* |
| *hsa-miR-1202* | *hsa-miR-190a-3p* | *hsa-miR-331-3p* | *hsa-miR-591* | *hsa-miR-498* |
| *hsa-miR-1203* | *hsa-miR-190a-5p* | *hsa-miR-331-5p* | *hsa-miR-592* | *hsa-miR-499a-3p* |
| *hsa-miR-1204* | *hsa-miR-190b* | *hsa-miR-335-5p* | *hsa-miR-593-3p* | *hsa-miR-499a-5p* |
| *hsa-miR-1205* | *hsa-miR-191-5p* | *hsa-miR-337-3p* | *hsa-miR-595* | *hsa-miR-499b-3p* |
| *hsa-miR-1206* | *hsa-miR-1910-3p* | *hsa-miR-337-5p* | *hsa-miR-596* | *hsa-miR-499b-5p* |
| *hsa-miR-122-5p* | *hsa-miR-1910-5p* | *hsa-miR-338-5p* | *hsa-miR-597-5p* | *hsa-miR-5001-3p* |
| *hsa-miR-1224-3p* | *hsa-miR-1915-3p* | *hsa-miR-339-3p* | *hsa-miR-598-3p* | *hsa-miR-5001-5p* |
| *hsa-miR-1224-5p* | *hsa-miR-192-5p* | *hsa-miR-339-5p* | *hsa-miR-599* | *hsa-miR-500a-5p+hsa-miR-501-5p* |
| *hsa-miR-1226-3p* | *hsa-miR-193a-3p* | *hsa-miR-33a-5p* | *hsa-miR-600* | *hsa-miR-501-3p* |
| *hsa-miR-1228-3p* | *hsa-miR-193a-5p+hsa-miR-193b-5p* | *hsa-miR-33b-5p* | *hsa-miR-601* | *hsa-miR-5010-3p* |
| *hsa-miR-1233-3p* | *hsa-miR-193b-3p* | *hsa-miR-340-5p* | *hsa-miR-603* | *hsa-miR-5010-5p* |
| *hsa-miR-1234-3p* | *hsa-miR-194-5p* | *hsa-miR-342-3p* | *hsa-miR-604* | *hsa-miR-502-3p* |
| *hsa-miR-1236-3p* | *hsa-miR-195-5p* | *hsa-miR-342-5p* | *hsa-miR-605-5p* | *hsa-miR-502-5p* |
| *hsa-miR-124-3p* | *hsa-miR-196a-3p* | *hsa-miR-345-3p* | *hsa-miR-606* | *hsa-miR-503-3p* |
| *hsa-miR-1244* | *hsa-miR-196a-5p* | *hsa-miR-345-5p* | *hsa-miR-607* | *hsa-miR-503-5p* |
| *hsa-miR-1245a* | *hsa-miR-196b-5p* | *hsa-miR-346* | *hsa-miR-608* | *hsa-miR-504-3p* |
| *hsa-miR-1245b-3p* | *hsa-miR-197-3p* | *hsa-miR-34a-5p* | *hsa-miR-610* | *hsa-miR-504-5p* |
| *hsa-miR-1245b-5p* | *hsa-miR-197-5p* | *hsa-miR-34b-3p* | *hsa-miR-612* | *hsa-miR-505-3p* |
| *hsa-miR-1246* | *hsa-miR-1972* | *hsa-miR-34c-3p* | *hsa-miR-613* | *hsa-miR-506-3p* |
| *hsa-miR-1247-5p* | *hsa-miR-1973* | *hsa-miR-34c-5p* | *hsa-miR-614* | *hsa-miR-506-5p* |
| *hsa-miR-1248* | *hsa-miR-1976* | *hsa-miR-3605-3p* | *hsa-miR-615-3p* | *hsa-miR-507* |
| *hsa-miR-1249-3p* | *hsa-miR-198* | *hsa-miR-3605-5p* | *hsa-miR-615-5p* | *hsa-miR-508-3p* |
| *hsa-miR-1249-5p* | *hsa-miR-199a-3p+hsa-miR-199b-3p* | *hsa-miR-361-3p* | *hsa-miR-616-3p* | *hsa-miR-508-5p* |
| *hsa-miR-1250-5p* | *hsa-miR-199a-5p* | *hsa-miR-361-5p* | *hsa-miR-617* | *hsa-miR-509-3-5p* |
| *hsa-miR-1252-5p* | *hsa-miR-199b-5p* | *hsa-miR-3613-3p* | *hsa-miR-619-3p* | *hsa-miR-509-3p* |
| *hsa-miR-1253* | *hsa-miR-19a-3p* | *hsa-miR-3613-5p* | *hsa-miR-620* | *hsa-miR-509-5p* |
| *hsa-miR-1254* | *hsa-miR-19b-3p* | *hsa-miR-3614-3p* | *hsa-miR-624-3p* | *hsa-miR-510-3p* |
| *hsa-miR-1255a* | *hsa-miR-200a-3p* | *hsa-miR-3614-5p* | *hsa-miR-625-5p* | *hsa-miR-510-5p* |
| *hsa-miR-1255b-5p* | *hsa-miR-200b-3p* | *hsa-miR-3615* | *hsa-miR-626* | *hsa-miR-511-5p* |
| *hsa-miR-1257* | *hsa-miR-200c-3p* | *hsa-miR-362-3p* | *hsa-miR-627-3p* | *hsa-miR-512-3p* |
| *hsa-miR-1258* | *hsa-miR-202-3p* | *hsa-miR-362-5p* | *hsa-miR-627-5p* | *hsa-miR-512-5p* |
| *hsa-miR-125a-3p* | *hsa-miR-203a-3p* | *hsa-miR-363-3p* | *hsa-miR-628-3p* | *hsa-miR-513a-3p* |
| *hsa-miR-125a-5p* | *hsa-miR-203a-5p* | *hsa-miR-363-5p* | *hsa-miR-628-5p* | *hsa-miR-513a-5p* |
| *hsa-miR-125b-5p* | *hsa-miR-204-5p* | *hsa-miR-365a-3p+hsa-miR-365b-3p* | *hsa-miR-629-5p* | *hsa-miR-513b-5p* |
| *hsa-miR-126-3p* | *hsa-miR-205-5p* | *hsa-miR-365b-5p* | *hsa-miR-630* | *hsa-miR-513c-3p* |
| *hsa-miR-1260a* | *hsa-miR-2053* | *hsa-miR-367-3p* | *hsa-miR-631* | *hsa-miR-513c-5p* |
| *hsa-miR-1260b* | *hsa-miR-206* | *hsa-miR-369-3p* | *hsa-miR-637* | *hsa-miR-514a-3p* |
| *hsa-miR-1261* | *hsa-miR-208a-3p* | *hsa-miR-369-5p* | *hsa-miR-638* | *hsa-miR-514a-5p* |
| *hsa-miR-1262* | *hsa-miR-208b-3p* | *hsa-miR-3690* | *hsa-miR-639* | *hsa-miR-514b-3p* |
| *hsa-miR-1264* | *hsa-miR-208b-5p* | *hsa-miR-370-3p* | *hsa-miR-640* | *hsa-miR-514b-5p* |
| *hsa-miR-1266-5p* | *hsa-miR-20a-5p+hsa-miR-20b-5p* | *hsa-miR-370-5p* | *hsa-miR-641* | *hsa-miR-515-3p* |
| *hsa-miR-1268a* | *hsa-miR-21-5p* | *hsa-miR-371a-5p* | *hsa-miR-642a-3p* | *hsa-miR-515-5p* |
| *hsa-miR-1268b* | *hsa-miR-210-3p* | *hsa-miR-371b-5p* | *hsa-miR-642a-5p* | *hsa-miR-516a-3p+hsa-miR-516b-3p* |
| *hsa-miR-1269a* | *hsa-miR-210-5p* | *hsa-miR-372-3p* | *hsa-miR-643* | *hsa-miR-516a-5p* |
| *hsa-miR-1269b* | *hsa-miR-211-3p* | *hsa-miR-373-3p* | *hsa-miR-644a* | *hsa-miR-516b-5p* |
| *hsa-miR-127-3p* | *hsa-miR-211-5p* | *hsa-miR-374a-3p* | *hsa-miR-648* | *hsa-miR-517a-3p* |
| *hsa-miR-127-5p* | *hsa-miR-2110* | *hsa-miR-374a-5p* | *hsa-miR-649* | *hsa-miR-517b-3p* |
| *hsa-miR-1270* | *hsa-miR-2113* | *hsa-miR-374b-5p* | *hsa-miR-650* | *hsa-miR-517c-3p+hsa-miR-519a-3p* |
| *hsa-miR-1271-3p* | *hsa-miR-2116-5p* | *hsa-miR-374c-5p* | *hsa-miR-6503-3p* | *hsa-miR-518b* |
| *hsa-miR-1271-5p* | *hsa-miR-2117* | *hsa-miR-375* | *hsa-miR-6503-5p* | *hsa-miR-518c-3p* |
| *hsa-miR-1272* | *hsa-miR-212-3p* | *hsa-miR-376a-2-5p* | *hsa-miR-651-3p* | *hsa-miR-518d-3p* |
| *hsa-miR-1273c* | *hsa-miR-214-3p* | *hsa-miR-376a-3p* | *hsa-miR-651-5p* | *hsa-miR-518e-3p* |
| *hsa-miR-1275* | *hsa-miR-215-5p* | *hsa-miR-376b-3p* | *hsa-miR-6511a-3p* | *hsa-miR-518f-3p* |
| *hsa-miR-1276* | *hsa-miR-216a-5p* | *hsa-miR-376c-3p* | *hsa-miR-6511a-5p* | *hsa-miR-5196-3p+hsa-miR-6732-3p* |
| *hsa-miR-1277-3p* | *hsa-miR-216b-5p* | *hsa-miR-376c-5p* | *hsa-miR-652-3p* | *hsa-miR-5196-5p* |
| *hsa-miR-1278* | *hsa-miR-217* | *hsa-miR-377-3p* | *hsa-miR-652-5p* | *hsa-miR-519b-3p* |
| *hsa-miR-1279* | *hsa-miR-218-5p* | *hsa-miR-378b* | *hsa-miR-654-3p* | *hsa-miR-519b-5p+hsa-miR-519c-5p+hsa-miR-523-5p+hsa-miR-518e-5p+hsa-miR-522-5p+hsa-miR-519a-5p* |
| *hsa-miR-128-1-5p* | *hsa-miR-219a-1-3p* | *hsa-miR-378c* | *hsa-miR-654-5p* | *hsa-miR-519c-3p* |
| *hsa-miR-128-2-5p* | *hsa-miR-219a-2-3p* | *hsa-miR-378d* | *hsa-miR-655-3p* | *hsa-miR-519d-3p* |
| *hsa-miR-128-3p* | *hsa-miR-219a-5p* | *hsa-miR-378e* | *hsa-miR-656-3p* | *hsa-miR-519e-3p* |
| *hsa-miR-1281* | *hsa-miR-219b-3p* | *hsa-miR-378f* | *hsa-miR-660-3p* | *hsa-miR-520a-3p* |
| *hsa-miR-1283* | *hsa-miR-22-3p* | *hsa-miR-378g* | *hsa-miR-660-5p* | *hsa-miR-520a-5p* |
| *hsa-miR-1285-3p* | *hsa-miR-221-3p* | *hsa-miR-378h* | *hsa-miR-661* | *hsa-miR-520b* |
| *hsa-miR-1285-5p* | *hsa-miR-221-5p* | *hsa-miR-378i* | *hsa-miR-663a* | *hsa-miR-520c-3p* |
| *hsa-miR-1286* | *hsa-miR-222-3p* | *hsa-miR-379-5p* | *hsa-miR-664a-3p* | *hsa-miR-520d-3p* |
| *hsa-miR-1287-3p* | *hsa-miR-223-3p* | *hsa-miR-380-3p* | *hsa-miR-664b-3p* | *hsa-miR-520d-5p+hsa-miR-527+hsa-miR-518a-5p* |
| *hsa-miR-1287-5p* | *hsa-miR-224-5p* | *hsa-miR-381-3p* | *hsa-miR-664b-5p* | *hsa-miR-520e* |
| *hsa-miR-1288-3p* | *hsa-miR-2278* | *hsa-miR-381-5p* | *hsa-miR-665* | *hsa-miR-520f-3p* |
| *hsa-miR-1289* | *hsa-miR-23a-3p* | *hsa-miR-382-3p* | *hsa-miR-671-3p* | *hsa-miR-520g-3p* |
| *hsa-miR-129-2-3p* | *hsa-miR-23b-3p* | *hsa-miR-382-5p* | *hsa-miR-671-5p* | *hsa-miR-520h* |
| *hsa-miR-129-5p* | *hsa-miR-23c* | *hsa-miR-383-5p* | *hsa-miR-6720-3p* | *hsa-miR-521* |
| *hsa-miR-1290* | *hsa-miR-24-3p* | *hsa-miR-384* | *hsa-miR-6721-5p* | *hsa-miR-522-3p* |
| *hsa-miR-1291* | *hsa-miR-25-3p* | *hsa-miR-3916* | *hsa-miR-6724-5p* | *hsa-miR-523-3p* |
| *hsa-miR-1293* | *hsa-miR-25-5p* | *hsa-miR-3918* | *hsa-miR-675-5p* | *hsa-miR-524-3p* |
| *hsa-miR-1295a* | *hsa-miR-2682-5p* | *hsa-miR-3928-3p* | *hsa-miR-7-5p* | *hsa-miR-525-3p* |
| *hsa-miR-1296-3p* | *hsa-miR-26a-5p* | *hsa-miR-3934-5p* | *hsa-miR-708-5p* | *hsa-miR-525-5p* |
| *hsa-miR-1296-5p* | *hsa-miR-26b-5p* | *hsa-miR-409-3p* | *hsa-miR-744-5p* | *hsa-miR-526a+hsa-miR-518c-5p+hsa-miR-518d-5p* |
| *hsa-miR-1297* | *hsa-miR-27a-3p* | *hsa-miR-409-5p* | *hsa-miR-758-3p+hsa-miR-411-3p* | *hsa-miR-526b-5p* |
| *hsa-miR-1298-5p* | *hsa-miR-27b-3p* | *hsa-miR-410-3p* | *hsa-miR-758-5p* | *hsa-miR-532-3p* |
| *hsa-miR-1299* | *hsa-miR-28-3p* | *hsa-miR-411-5p* | *hsa-miR-760* | *hsa-miR-532-5p* |
| *hsa-miR-1301-3p* | *hsa-miR-28-5p* | *hsa-miR-412-3p* | *hsa-miR-761* | *hsa-miR-539-3p* |
| *hsa-miR-1302* | *hsa-miR-296-3p* | *hsa-miR-421* | *hsa-miR-764* | *hsa-miR-539-5p* |
| *hsa-miR-1303* | *hsa-miR-296-5p* | *hsa-miR-422a* | *hsa-miR-765* | *hsa-miR-541-3p* |
| *hsa-miR-1304-3p* | *hsa-miR-297* | *hsa-miR-423-3p* | *hsa-miR-766-3p* | *hsa-miR-542-3p* |
| *hsa-miR-1304-5p* | *hsa-miR-298* | *hsa-miR-423-5p* | *hsa-miR-766-5p* | *hsa-miR-542-5p* |
| *hsa-miR-1305* | *hsa-miR-299-3p* | *hsa-miR-424-5p* | *hsa-miR-767-3p* | *hsa-miR-543* |
| *hsa-miR-1306-3p* | *hsa-miR-299-5p* | *hsa-miR-425-5p* | *hsa-miR-767-5p* | *hsa-miR-544a* |
| *hsa-miR-1306-5p* | *hsa-miR-29a-3p* | *hsa-miR-4284* | *hsa-miR-769-3p* | *hsa-miR-545-3p* |
| *hsa-miR-1307-3p* | *hsa-miR-29b-3p* | *hsa-miR-4286* | *hsa-miR-769-5p* | *hsa-miR-548a-3p* |
| *hsa-miR-1307-5p* | *hsa-miR-29c-3p* | *hsa-miR-429* | *hsa-miR-770-5p* | *hsa-miR-548a-5p* |
| *hsa-miR-130a-3p* | *hsa-miR-300* | *hsa-miR-431-5p* | *hsa-miR-802* | *hsa-miR-548aa+hsa-miR-548t-3p* |
| *hsa-miR-130b-3p* | *hsa-miR-301a-3p* | *hsa-miR-432-5p* | *hsa-miR-873-3p* | *hsa-miR-548ad-3p* |
| *hsa-miR-132-3p* | *hsa-miR-301a-5p* | *hsa-miR-433-3p* | *hsa-miR-873-5p* | *hsa-miR-548ah-5p* |
| *hsa-miR-1322* | *hsa-miR-301b-3p* | *hsa-miR-433-5p* | *hsa-miR-874-3p* | *hsa-miR-548ai+hsa-miR-570-5p* |
| *hsa-miR-1323* | *hsa-miR-301b-5p* | *hsa-miR-4421* | *hsa-miR-874-5p* | *hsa-miR-548ak* |
| *hsa-miR-133a-3p* | *hsa-miR-302a-3p* | *hsa-miR-4425* | *hsa-miR-875-3p* | *hsa-miR-548al* |
| *hsa-miR-133a-5p* | *hsa-miR-302a-5p* | *hsa-miR-4431* | *hsa-miR-876-3p* | *hsa-miR-548ar-3p* |
| *hsa-miR-133b* | *hsa-miR-302b-3p* | *hsa-miR-4435* | *hsa-miR-876-5p* | *hsa-miR-548ar-5p* |
| *hsa-miR-134-3p* | *hsa-miR-302c-3p* | *hsa-miR-4443* | *hsa-miR-877-5p* | *hsa-miR-548b-3p* |
| *hsa-miR-134-5p+hsa-miR-6728-5p* | *hsa-miR-302d-3p* | *hsa-miR-4448* | *hsa-miR-885-3p* | *hsa-miR-548c-5p+hsa-miR-548o-5p+hsa-miR-548am-5p* |
| *hsa-miR-135a-5p* | *hsa-miR-302e* | *hsa-miR-4451* | *hsa-miR-885-5p* | *hsa-miR-548d-3p* |
| *hsa-miR-135b-5p* | *hsa-miR-302f* | *hsa-miR-4454+hsa-miR-7975* | *hsa-miR-887-3p* | *hsa-miR-548d-5p* |
| *hsa-miR-136-5p* | *hsa-miR-3065-3p* | *hsa-miR-4455* | *hsa-miR-887-5p* | *hsa-miR-548e-3p* |
| *hsa-miR-137* | *hsa-miR-3065-5p* | *hsa-miR-4458* | *hsa-miR-888-5p* | *hsa-miR-548e-5p* |
| *hsa-miR-138-5p* | *hsa-miR-3074-3p* | *hsa-miR-4461* | *hsa-miR-889-3p* | *hsa-miR-548g-3p* |
| *hsa-miR-139-3p* | *hsa-miR-30a-3p* | *hsa-miR-448* | *hsa-miR-890* | *hsa-miR-548h-5p* |
| *hsa-miR-139-5p* | *hsa-miR-30a-5p* | *hsa-miR-4485-3p* | *hsa-miR-891a-5p* | *hsa-miR-548i* |
| *hsa-miR-140-3p* | *hsa-miR-30b-5p* | *hsa-miR-4488* | *hsa-miR-891b* | *hsa-miR-548j-3p* |
| *hsa-miR-140-5p* | *hsa-miR-30c-5p* | *hsa-miR-449a* | *hsa-miR-892a* | *hsa-miR-548j-5p* |
| *hsa-miR-141-3p* | *hsa-miR-30d-5p* | *hsa-miR-449b-5p* | *hsa-miR-892b* | *hsa-miR-548k* |
| *hsa-miR-142-3p* | *hsa-miR-30e-3p* | *hsa-miR-449c-5p* | *hsa-miR-9-5p* | *hsa-miR-548l* |
| *hsa-miR-142-5p* | *hsa-miR-30e-5p* | *hsa-miR-450a-1-3p* | *hsa-miR-922* | *hsa-miR-548m* |
| *hsa-miR-143-3p* | *hsa-miR-31-5p* | *hsa-miR-450a-2-3p* | *hsa-miR-924* | *hsa-miR-548n* |
| *hsa-miR-144-3p* | *hsa-miR-3127-5p* | *hsa-miR-450a-5p* | *hsa-miR-92a-1-5p* | *hsa-miR-548o-3p+hsa-miR-548ah-3p+hsa-miR-548av-3p* |
| *hsa-miR-145-5p* | *hsa-miR-3130-3p* | *hsa-miR-450b-3p* | *hsa-miR-92a-3p* | *hsa-miR-548q* |
| *hsa-miR-1469* | *hsa-miR-3131* | *hsa-miR-450b-5p* | *hsa-miR-92b-3p* | *hsa-miR-548v* |
| *hsa-miR-146a-5p* | *hsa-miR-3136-5p* | *hsa-miR-4516* | *hsa-miR-93-5p* | *hsa-miR-548y* |
| *hsa-miR-146b-3p* | *hsa-miR-3140-3p* | *hsa-miR-451a* | *hsa-miR-933* | *hsa-miR-548z+hsa-miR-548h-3p* |
| *hsa-miR-146b-5p* | *hsa-miR-3140-5p* | *hsa-miR-452-5p* | *hsa-miR-934* | *hsa-miR-549a* |
| *hsa-miR-147a* | *hsa-miR-3144-3p* | *hsa-miR-4521* | *hsa-miR-935* | *hsa-miR-550a-5p* |
| *hsa-miR-147b* | *hsa-miR-3144-5p* | *hsa-miR-4524a-5p* | *hsa-miR-936* | *hsa-miR-551a* |
| *hsa-miR-148a-3p* | *hsa-miR-3147* | *hsa-miR-4531* | *hsa-miR-937-3p* | *hsa-miR-551b-3p* |
| *hsa-miR-148b-3p* | *hsa-miR-3150b-3p* | *hsa-miR-4532* | *hsa-miR-939-5p* | *hsa-miR-552-3p* |
| *hsa-miR-149-5p* | *hsa-miR-3151-5p* | *hsa-miR-4536-3p* | *hsa-miR-940* | *hsa-miR-553* |
| *hsa-miR-150-5p* | *hsa-miR-3158-3p* | *hsa-miR-4536-5p* | *hsa-miR-941* | *hsa-miR-554* |
| *hsa-miR-151a-3p* | *hsa-miR-3161* | *hsa-miR-454-3p* | *hsa-miR-942-3p* | *hsa-miR-555* |
| *hsa-miR-151a-5p* | *hsa-miR-3164* | *hsa-miR-455-3p* | *hsa-miR-942-5p* | *hsa-miR-556-3p* |
| *hsa-miR-151b* | *hsa-miR-3168* | *hsa-miR-455-5p* | *hsa-miR-944* | *hsa-miR-556-5p* |
| *hsa-miR-152-3p* | *hsa-miR-3179* | *hsa-miR-4647* | *hsa-miR-95-3p* | *hsa-miR-561-3p* |
| *hsa-miR-152-5p* | *hsa-miR-3180* | *hsa-miR-4707-3p* | *hsa-miR-96-5p* | *hsa-miR-561-5p* |
| *hsa-miR-153-3p* | *hsa-miR-3180-3p* | *hsa-miR-4707-5p* | *hsa-miR-98-3p* | *hsa-miR-562* |

STable 3: List of Putative Human Target Genes Obtained from MirDIP

| 3347 Putative Human Target Genes | | | | |
| --- | --- | --- | --- | --- |
| *RSBN1* | *CPEB2* | *NUFIP2* | *TMEM243* | *CDS2* |
| *DDIT4* | *ZNF654* | *SSR1* | *NAP1L4* | *SNRPC* |
| *SIRT1* | *XIAP* | *USP10* | *STX12* | *ALDH5A1* |
| *IRF4* | *TFAP2B* | *PRKCB* | *TGIF1* | *NWD2* |
| *PTEN* | *HNRNPA3* | *MAP4K5* | *PICALM* | *AOAH* |
| *H3-3B* | *PRDM16* | *RGS5* | *CALD1* | *BASP1* |
| *LAMC1* | *RNPS1* | *DVL3* | *IGSF3* | *CKS1B* |
| *LIN7C* | *MBNL2* | *EPAS1* | *ADCY9* | *STIM1* |
| *ARID3B* | *LBR* | *IGF2BP3* | *ATG12* | *TRANK1* |
| *PCTP* | *PSD3* | *NUDT11* | *RNF111* | *CASD1* |
| *ZNF740* | *ACTL6A* | *DENND2C* | *PLS3* | *GRIA1* |
| *FMNL2* | *KBTBD2* | *TBC1D19* | *SEPSECS* | *GTF3C2* |
| *MSL2* | *ONECUT2* | *CANX* | *VPS26A* | *GNAO1* |
| *RBM15* | *TMEM106B* | *UNC5D* | *SDK1* | *ICAM5* |
| *GATM* | *SPRY1* | *TRIB1* | *SMARCE1* | *TRIM13* |
| *GRB10* | *C11orf87* | *KIF1B* | *PLPPR1* | *BRSK2* |
| *SEPHS1* | *HMGB1* | *YWHAG* | *PCDHGA6* | *PCSK1* |
| *CCN1* | *TARDBP* | *DDX17* | *ARHGEF6* | *CRACR2B* |
| *IL17RD* | *NAA50* | *PHC2* | *VEGFD* | *ERI1* |
| *GCNT1* | *SESN3* | *PCDHGA9* | *IRX5* | *RAB9B* |
| *OSBPL9* | *SLC16A10* | *PKNOX2* | *CCDC117* | *SHOC2* |
| *ESR1* | *THRB* | *RPL15* | *ZDHHC22* | *TMSB4X* |
| *SYNE1* | *KPNA3* | *CRIM1* | *DSTYK* | *ATP5PB* |
| *STARD13* | *SLC38A2* | *ST8SIA2* | *BTBD1* | *SRC* |
| *STAG2* | *LDB2* | *SLC16A1* | *CADM2* | *SUCLG2* |
| *TP53INP1* | *NFE2L2* | *FNDC4* | *TENT4B* | *GPR55* |
| *CHD9* | *ZFC3H1* | *PEAK1* | *CTNND2* | *MOG* |
| *PPP4R3A* | *CDK17* | *GANAB* | *MED21* | *C6orf132* |
| *KLF13* | *ESRRG* | *CBX5* | *MAN2A2* | *DDX19A* |
| *SEMA4D* | *FIGN* | *RNF185* | *KREMEN1* | *NPHP3* |
| *SLC39A9* | *NAV3* | *FOXRED2* | *PEX5* | *GNS* |
| *C5orf24* | *UBASH3B* | *EPC2* | *AP2M1* | *GALNT6* |
| *SH3TC2* | *SNX27* | *ARFGEF2* | *RAB28* | *DHCR24* |
| *RAB5B* | *AGPAT3* | *KSR2* | *NDRG3* | *MPP3* |
| *AKT3* | *BCL7A* | *TPM3* | *TMEM169* | *GATD1* |
| *GRM5* | *HOXC11* | *SON* | *CCDC113* | *L3MBTL1* |
| *VPS4B* | *ZBTB43* | *ERLIN2* | *XKR9* | *FOXE3* |
| *ARPC5* | *OSBPL3* | *ZIC5* | *ACTR2* | *ATF3* |
| *TMEM168* | *CD2AP* | *NKTR* | *SOX7* | *ZNF117* |
| *MAPK14* | *GRAMD2B* | *JADE3* | *MYLK4* | *NCDN* |
| *CYRIB* | *SLC39A10* | *STEAP2* | *SORT1* | *DDX3X* |
| *ELOVL6* | *NFIX* | *ZC3H4* | *CTSE* | *DOCK4* |
| *MECP2* | *PRDM5* | *PHF12* | *RGS6* | *ZIK1* |
| *IKZF4* | *GRIA2* | *FBXO21* | *WAPL* | *MKRN1* |
| *PHF8* | *UBR1* | *CHSY1* | *PCDHGA4* | *ELFN2* |
| *MEIS2* | *MEX3C* | *TOP2B* | *CYFIP2* | *FAM98A* |
| *SATB2* | *NDRG1* | *KCTD20* | *LYNX1* | *SLC16A2* |
| *LFNG* | *PPP1CB* | *RNF114* | *HMGA1* | *KRT1* |
| *TNFAIP3* | *SOCS6* | *YAP1* | *ANKRD13B* | *SDR39U1* |
| *CPEB1* | *CPEB4* | *SCN3A* | *LAMP2* | *NDUFA5* |
| *PLCXD3* | *CALM2* | *PHF20L1* | *CBFA2T2* | *BLOC1S1* |
| *DENND6A* | *RUNX1T1* | *DDX54* | *PCDHGB1* | *PIK3R1* |
| *LIN28A* | *SGMS1* | *FARP1* | *PCDHGB7* | *DMTN* |
| *CCDC47* | *SMARCAD1* | *PLCB1* | *SUN2* | *FAR1* |
| *IPO7* | *C18orf25* | *DISC1* | *SLC1A2* | *HLA-DOA* |
| *TRPM7* | *TMEM108* | *ZDHHC15* | *CD209* | *FBXO41* |
| *SEMA4C* | *ZRANB2* | *SLC22A23* | *SENP7* | *TBC1D2B* |
| *MTF1* | *CLDN18* | *ADAM10* | *RNF11* | *KDM4B* |
| *PPP1R15B* | *GATAD2B* | *PCDHGB4* | *NSD3* | *TRHDE* |
| *EPB41L2* | *TRAM1* | *ATP13A3* | *ASCL1* | *ADGRG1* |
| *MAX* | *CENPK* | *KDM4A* | *ARL4C* | *MLLT6* |
| *TNFSF4* | *PTBP2* | *TRERF1* | *ARL8B* | *DCTN5* |
| *MTHFR* | *AHR* | *RAG1* | *DLX6* | *CRAMP1* |
| *ERBB3* | *RFC1* | *C6orf62* | *STC2* | *NRGN* |
| *FAM118A* | *DCUN1D5* | *ZNF641* | *OTUD7B* | *DCAF8L1* |
| *RYBP* | *RPS6KA3* | *DAB2* | *MTSS1* | *RMND5A* |
| *IST1* | *BAZ2B* | *STAT1* | *PCDHA6* | *LRRTM4* |
| *SERTAD3* | *STXBP4* | *TCF12* | *WDR36* | *ANKRD40* |
| *ADAMTS3* | *RFX7* | *LMO3* | *DDX58* | *OOSP2* |
| *MAT2A* | *TAPT1* | *SLC16A12* | *POU2F3* | *CLN8* |
| *CLIC4* | *ADSS2* | *CTDSP2* | *FOLH1* | *ZNF626* |
| *NECAB3* | *RAI14* | *TAB3* | *INA* | *EEF2K* |
| *MON2* | *DPY19L3* | *LRCH1* | *RAB3A* | *ALG1* |
| *WWC2* | *SMAD5* | *RFLNB* | *SMYD5* | *KHDC4* |
| *DOCK3* | *TMEM263* | *POLH* | *GRIN2A* | *TPD52* |
| *ZMYM4* | *CAP1* | *SLC38A1* | *RBM39* | *APH1B* |
| *KCTD9* | *CIT* | *OSBP2* | *MAP3K9* | *ISOC2* |
| *PRPF38A* | *NLK* | *SCN3B* | *MTCL1* | *RPN2* |
| *TYRO3* | *BTAF1* | *DBT* | *SLC9A1* | *COL5A3* |
| *WDR82P1* | *SELENOP* | *PDE4D* | *LRRC4* | *SYNC* |
| *RHOQ* | *NKAIN2* | *RNF182* | *AGO4* | *GIGYF1* |
| *RIC8B* | *DCUN1D1* | *RAB8B* | *SMARCD2* | *WDR20* |
| *BTG1* | *AKIRIN1* | *BHLHE41* | *KLHL13* | *RALY* |
| *SBNO1* | *CAV1* | *PGPEP1* | *LEMD3* | *EFNA3* |
| *NCOA1* | *G3BP2* | *PKIA* | *KLHL3* | *MACC1* |
| *MFHAS1* | *PELI1* | *EHBP1* | *RBPJ* | *SEMA5A* |
| *PDSS1* | *JADE1* | *ENTPD7* | *PKN2* | *HOXD11* |
| *KCNS3* | *GGNBP2* | *HOXB5* | *ORMDL3* | *TSC22D4* |
| *TOR2A* | *LARP4* | *BCAT2* | *PDP1* | *DPYSL3* |
| *ATP10D* | *GNAI3* | *KLHL42* | *PCDHAC1* | *TMEM132C* |
| *FOXN3* | *MYH10* | *MOB3B* | *BBOX1* | *WAC* |
| *GTPBP2* | *ARMC8* | *UBN2* | *DCLRE1C* | *CYP20A1* |
| *BAK1* | *KMT5B* | *CIP2A* | *MIER3* | *ARGLU1* |
| *STK39* | *TNFSF11* | *PGGT1B* | *WDR72* | *BTN3A2* |
| *CCNJ* | *CD34* | *GLRA2* | *SCFD1* | *SEC14L1* |
| *LBH* | *POU4F2* | *CHODL* | *ZNF800* | *DKK1* |
| *WRNIP1* | *GPM6B* | *GJA1* | *SEC22C* | *ANXA4* |
| *ARHGEF12* | *GAN* | *CTNNA1* | *SETDB1* | *PSD4* |
| *RGS2* | *SREK1* | *TIA1* | *PPIL1* | *INO80* |
| *ENPEP* | *PAN2* | *TCFL5* | *KLHL9* | *GRIK3* |
| *CYTH3* | *C1orf174* | *DNAJC6* | *FAM104A* | *KAZN* |
| *NEU1* | *HOXA1* | *SIX4* | *WASHC4* | *ATP6V1B2* |
| *ETS1* | *HOOK3* | *SOX5* | *NFYB* | *ZBP1* |
| *TRIM71* | *ZIC4* | *SEC23IP* | *RBM24* | *SIPA1L2* |
| *KCTD15* | *ZZEF1* | *APOOL* | *FBXO42* | *LMBR1L* |
| *KHNYN* | *DCX* | *RP2* | *ZNF532* | *HOMER2* |
| *CDK6* | *PAK2* | *YPEL1* | *SPINDOC* | *CALM3* |
| *WASL* | *CNOT2* | *DDX6* | *TRAPPC14* | *CX3CR1* |
| *PPM1K* | *MIER1* | *THAP2* | *PPP1R9B* | *ABI1* |
| *ABCC5* | *NAP1L1* | *ELF2* | *CACNA1D* | *CPNE5* |
| *MAP3K10* | *PRKAR2B* | *PPP2CB* | *TFE3* | *ATRN* |
| *SCYL3* | *MLLT3* | *LYRM2* | *APOBEC3F* | *DLGAP1* |
| *MAGI2* | *KCNA1* | *MAPK6* | *HNRNPDL* | *POLD3* |
| *DUS1L* | *XPO4* | *ANKRD46* | *SDC1* | *ATP1A3* |
| *ORC2* | *NDFIP1* | *SSX2IP* | *SAR1A* | *SPTB* |
| *YWHAZ* | *PAX9* | *IGF2BP1* | *NFIC* | *ZNF385A* |
| *MYT1* | *SLC5A3* | *SYNJ1* | *LGI1* | *ST14* |
| *PODXL* | *NOVA1* | *SUMO1* | *MTDH* | *RHOBTB1* |
| *BAP1* | *FXR1* | *OCA2* | *ZNF557* | *PPM1H* |
| *KAT6A* | *PAFAH1B2* | *LHX6* | *DGKI* | *ZNF257* |
| *FNBP4* | *FNBP1L* | *GOLT1B* | *NBPF15* | *PIM1* |
| *NIPAL4* | *TEAD1* | *ILF3* | *KDM2A* | *C1orf216* |
| *FUT9* | *CADM1* | *AHCTF1* | *NPAT* | *RTP1* |
| *ZBTB39* | *PEX5L* | *CTNND1* | *CD93* | *CAPZB* |
| *SEC63* | *INSM1* | *AMACR* | *ZNF444* | *POLR3B* |
| *EVA1A* | *PPP1CC* | *DCC* | *STK17A* | *FBN3* |
| *KCNK10* | *DLC1* | *EPPIN* | *UBE2V1* | *SEMA3G* |
| *SNAP91* | *GLUL* | *PITPNC1* | *TMEM41B* | *RNF157* |
| *LPGAT1* | *CALM1* | *DOK6* | *CCL22* | *RASA4* |
| *CBL* | *SPOPL* | *ARFGAP3* | *NEK11* | *NUP62* |
| *UBN1* | *SEMA6D* | *SCARB2* | *RNF4* | *ITSN1* |
| *IL13RA1* | *DOCK10* | *LYSMD3* | *ADO* | *ZBTB32* |
| *NPL* | *GRIA3* | *LRP2* | *THAP6* | *WDTC1* |
| *ABHD3* | *LANCL1* | *PDPK1* | *NAA16* | *NISCH* |
| *BMF* | *TRIO* | *ACAP2* | *HIF3A* | *CDYL2* |
| *ATP8A1* | *SS18* | *MPZ* | *TGFBR2* | *PLPPR2* |
| *LIFR* | *ZFX* | *LHX2* | *UBE2D1* | *NSMF* |
| *ETV1* | *DAPK1* | *ARID5B* | *ARG2* | *MAPKAPK2* |
| *KAT6B* | *TFDP2* | *ANKRD52* | *TSPAN11* | *CRTC3* |
| *MAP3K11* | *HNRNPK* | *IL21R* | *ABCC4* | *VAMP1* |
| *CSDE1* | *KANK1* | *CEP55* | *CASKIN2* | *ETV3* |
| *CDH5* | *CCNC* | *MFAP3L* | *HDAC2* | *DAB2IP* |
| *MTUS1* | *UBXN7* | *ITM2B* | *UBE2W* | *PRX* |
| *TRIAP1* | *AP1AR* | *NR1D2* | *MFN2* | *SKI* |
| *WASF1* | *SLC30A7* | *MCMDC2* | *DAAM2* | *VSTM2L* |
| *RBMS1* | *JPH1* | *FAM222B* | *KCNA7* | *PRKG1* |
| *CHD7* | *ANKRD50* | *FGL2* | *CRTAP* | *EVI5L* |
| *SLC6A5* | *GABPA* | *SLK* | *ZYG11B* | *TBX15* |
| *TET2* | *ACTN1* | *ARPC2* | *ZFP36L2* | *BAHD1* |
| *OLFML2A* | *AZIN1* | *GPR3* | *NAV1* | *SREBF1* |
| *PRDM1* | *LYPLA1* | *PPP2R1B* | *TSPAN14* | *PABIR2* |
| *SMARCA5* | *ERBIN* | *HSPA13* | *MYRF* | *RAB1B* |
| *NTRK2* | *PDIA3* | *FBXL17* | *MKLN1* | *HCN2* |
| *BRWD3* | *GMFB* | *DHX57* | *POLE3* | *ARL8A* |
| *PDIK1L* | *GPM6A* | *VPS37B* | *DEF8* | *KLF16* |
| *VEZF1* | *PDZRN3* | *RPS6KB1* | *ATXN7* | *FURIN* |
| *FBXW4* | *CUX1* | *RELCH* | *PCDHA10* | *GABRA3* |
| *PDS5A* | *PABPC3* | *LUC7L3* | *IBA57* | *HCRTR1* |
| *ZCCHC14* | *ARMCX3* | *PCDHGA2* | *IL22* | *MAFB* |
| *UBE2G1* | *ZFAND6* | *NR5A2* | *PRELP* | *CDK2* |
| *NUP210* | *NR4A2* | *LYST* | *CARNMT1* | *BARX1* |
| *CSNK2A1* | *RANBP9* | *SERBP1* | *OSBP* | *SYDE1* |
| *CNOT6L* | *UBE2B* | *KAT2B* | *ZBTB18* | *SOS2* |
| *TBC1D1* | *EIF4G3* | *MAP1A* | *ITPKB* | *MGAT5B* |
| *HIF1AN* | *NRCAM* | *ISL1* | *NRN1* | *RORB* |
| *FRAT2* | *LPIN2* | *INAVA* | *SMCO4* | *MAP4* |
| *LCOR* | *PRPF19* | *CIRBP* | *CYP26B1* | *FOXK1* |
| *ABHD6* | *MAPT* | *DRD1* | *PCDHA2* | *SASH3* |
| *LRRC1* | *ATP2B1* | *PTPRT* | *SEC23B* | *L3MBTL4* |
| *KPNA6* | *KLHL12* | *MTA2* | *KCNN3* | *LDLR* |
| *PHOX2B* | *GARRE1* | *TLE2* | *B3GALT1* | *RBM8A* |
| *KDM3A* | *LRRC8B* | *TRAK2* | *UTY* | *PFN2* |
| *HIPK1* | *PTPN20* | *PLAG1* | *ERMP1* | *FAM135B* |
| *SLITRK6* | *ABCA1* | *DCBLD2* | *PRLR* | *PCSK9* |
| *DPY30* | *MBLAC2* | *IKZF2* | *RHPN2* | *CES3* |
| *LIPA* | *ACVR2A* | *ZNF546* | *HELZ* | *KLHDC3* |
| *DEUP1* | *NRBF2* | *ABL2* | *ELOVL2* | *EMC2* |
| *CGN* | *SRSF3* | *ICOS* | *STAG1* | *NACC1* |
| *FBXW7* | *SLC16A4* | *ZFHX3* | *CCNG2* | *CACNG4* |
| *FUT4* | *SYPL1* | *TLCD3A* | *CGGBP1* | *FETUB* |
| *SLC4A10* | *EFR3A* | *SRSF12* | *ZBTB44* | *SNX21* |
| *ZNF704* | *CHD5* | *ZNF507* | *RND2* | *OTUB1* |
| *TRUB1* | *ZZZ3* | *DMWD* | *SCD* | *RNF121* |
| *MYCN* | *GPR85* | *ASAP1* | *AKAP7* | *SALL1* |
| *GJD3* | *CSRNP3* | *EPB41L1* | *TBC1D5* | *CPLX2* |
| *BRWD1* | *SPOCK1* | *SLC22A5* | *PTPRO* | *SPNS1* |
| *SLC7A1* | *RAP2A* | *FCMR* | *MEIS1* | *MAP7D2* |
| *SNRK* | *PAM* | *CDON* | *RAB3GAP2* | *CYBRD1* |
| *PAFAH1B1* | *IGFBP3* | *RTF1* | *FBXO48* | *FOXD4L6* |
| *NPAS3* | *PDE3B* | *FEM1C* | *ANO3* | *TMUB1* |
| *OGN* | *FNDC3A* | *DNAJA2* | *CERS3* | *DTX3L* |
| *TIAM1* | *PCGF3* | *TFCP2* | *SRRM2* | *UBQLN4* |
| *HOMER1* | *FGF9* | *BTBD10* | *NUBPL* | *TMEM63C* |
| *NR3C1* | *CUL4B* | *INPP5B* | *SORBS3* | *LMNA* |
| *ARPP21* | *SCHIP1* | *PPP4R3B* | *FAM234A* | *LGI3* |
| *ZDHHC16* | *MGAT4A* | *CHD6* | *ENDOV* | *UBE2Q2* |
| *AGGF1* | *DPYSL2* | *GNPDA1* | *BEND3* | *LANCL2* |
| *BAZ2A* | *BACE1* | *C11orf58* | *PHF1* | *NBL1* |
| *PTGS1* | *AFF3* | *C15orf40* | *SAP130* | *GGA3* |
| *CAV3* | *MMP16* | *PPM1A* | *PLEC* | *ZBTB47* |
| *SUV39H1* | *TENM1* | *EPHA4* | *DIRAS2* | *RIPK4* |
| *POGK* | *NEBL* | *COG3* | *CLEC5A* | *PRRT2* |
| *MECOM* | *WNT5A* | *RAB33B* | *POLR2M* | *PDE4C* |
| *BCL9L* | *CHD1* | *GLP1R* | *AKAP11* | *SKIL* |
| *PURB* | *ZFYVE26* | *USP1* | *ATXN2L* | *SCARB1* |
| *BCL9* | *FBXW2* | *PSEN1* | *SLAMF8* | *FOXD4L1* |
| *MYNN* | *CNGA3* | *CHRNA5* | *GPR137B* | *RARG* |
| *SLC25A15* | *CELF2* | *MED1* | *GRIA4* | *DYNLL2* |
| *TRPS1* | *SERP1* | *SLC1A4* | *CMTM4* | *BCL3* |
| *CPSF6* | *FAM168B* | *CYCS* | *WDFY1* | *MBNL3* |
| *ANKRD13A* | *AFF2* | *GAB2* | *CILK1* | *SEMA4B* |
| *DRAM2* | *KTN1* | *CHD2* | *ARHGDIA* | *ZHX3* |
| *TRIB2* | *TRIT1* | *SECISBP2L* | *PLEKHO2* | *BBX* |
| *WARS1* | *ZBTB10* | *PTPRF* | *FZD5* | *BARHL1* |
| *SLC2A1* | *TPP2* | *TMED5* | *RPRD2* | *TIMP3* |
| *MSI1* | *BZW1* | *ADD3* | *TUBB2A* | *TMEM132B* |
| *LRRC73* | *NLGN4X* | *CAPZA1* | *UBAP2* | *PAQR6* |
| *CCNJL* | *FBXO45* | *ZNF264* | *UNC13C* | *BRSK1* |
| *DAZAP2* | *FRMD4A* | *TBL1XR1* | *SVOP* | *E2F2* |
| *TGOLN2* | *SDAD1* | *ATP1B1* | *ZWINT* | *B4GALT2* |
| *RAPGEF5* | *TMEM65* | *FOXJ3* | *CD84* | *TSC22D2* |
| *LGALS1* | *SRSF7* | *UBE2J1* | *SIK3* | *VANGL1* |
| *RCOR1* | *PRKCE* | *UCHL5* | *SLC25A25* | *DNAJC5* |
| *NUS1* | *TENT5A* | *SLC46A3* | *IQSEC1* | *USP22* |
| *NKAPD1* | *CBLL1* | *ZFAND5* | *PMP22* | *UBP1* |
| *INO80D* | *N4BP2L1* | *PRKAG1* | *WWC1* | *AP3B1* |
| *ETF1* | *PPP2R2C* | *SHANK2* | *SASH1* | *SATB1* |
| *SRSF10* | *SCN2A* | *CDC42* | *RAN* | *PIP5K1C* |
| *TMEM123* | *RUNX2* | *SPATA2* | *PUS7L* | *MKNK2* |
| *CALCR* | *PTPRZ1* | *FAM120A* | *RBBP6* | *PHOSPHO1* |
| *SSTR3* | *TJP1* | *AGMO* | *ZNF697* | *GP5* |
| *ATXN1* | *EPDR1* | *PKM* | *PEX19* | *LRRC20* |
| *HERPUD2* | *FKBP5* | *LATS2* | *KIF21A* | *TBX3* |
| *ARF6* | *HERC1* | *ZNF711* | *SH3BGRL* | *ANO10* |
| *SOX11* | *CACNA1G* | *UHRF1BP1L* | *DCTN4* | *OTUD5* |
| *CDC42EP3* | *SLITRK2* | *ZMYND11* | *RGMA* | *PRSS22* |
| *TAF9B* | *PALLD* | *BMPR1A* | *EMP1* | *SORD* |
| *PRRC1* | *PLPBP* | *PXMP4* | *RAB15* | *WIPF1* |
| *ZSCAN29* | *VGLL3* | *KCND2* | *PCDHB13* | *ADAM11* |
| *UBE2H* | *ZIC3* | *CD47* | *FOLH1B* | *JUNB* |
| *USP37* | *RASA1* | *STAM* | *PLP1* | *OTUB2* |
| *SLC38A9* | *MORF4L1* | *ZMYM2* | *PKP4* | *COL23A1* |
| *NFIB* | *PASD1* | *ADCYAP1* | *SIM1* | *STAT5B* |
| *ELAVL2* | *SOX2* | *PCDHGA8* | *COQ7* | *ZNF853* |
| *DIS3L2* | *ARMC1* | *PPT2* | *ZNF2* | *DAGLA* |
| *ASB6* | *OGT* | *HEY1* | *ZNF451* | *TRAF7* |
| *NECAP1* | *RASAL2* | *IL6ST* | *SIK2* | *FOSB* |
| *GGA2* | *CELF1* | *PTAR1* | *VGLL4* | *FCHSD1* |
| *ENPP1* | *BRCA1* | *CSNK1A1* | *SNX29* | *FBXL18* |
| *ESRRA* | *LRP6* | *ARF1* | *NSD1* | *ZBTB1* |
| *RAB3D* | *RECK* | *PCDHGA3* | *FAHD1* | *RDH10* |
| *ITGA9* | *SLC2A3* | *TIGAR* | *RBFOX1* | *PWWP2A* |
| *APBB2* | *CNTN3* | *PGAP1* | *LEFTY2* | *BPIFB4* |
| *MAMDC2* | *SRPK2* | *NFASC* | *SPTLC1* | *ZCCHC24* |
| *MEGF9* | *RALBP1* | *EXOC5* | *EYA1* | *C19orf12* |
| *CLCN3* | *FYN* | *PPP1R12B* | *RRN3* | *ITGB3* |
| *TMEM135* | *PDCD6IP* | *KLHL28* | *ELK1* | *FN3K* |
| *TNRC6B* | *TNFRSF21* | *CHIC1* | *HNRNPUL2* | *FAM219A* |
| *FNDC3B* | *PPM1D* | *ANTXR2* | *PHYH* | *KLHL26* |
| *RPRD1B* | *FOXO3* | *TATDN3* | *RRAGD* | *CHST3* |
| *SANBR* | *KDM6A* | *OSBPL8* | *E2F1* | *TIMM13* |
| *BACH2* | *RRAS2* | *USP53* | *LPCAT3* | *ARHGAP6* |
| *GALNT14* | *UBE2E2* | *DLAT* | *OPA3* | *NPRL3* |
| *MFN1* | *SENP5* | *EGR2* | *NUFIP1* | *BRI3BP* |
| *OAZ2* | *CHMP2B* | *IGF1R* | *CNGB3* | *CHRNA2* |
| *ANPEP* | *ASF1A* | *PRRX1* | *ARNTL* | *IFIT2* |
| *NXT2* | *GFRA2* | *POU3F1* | *KLHL8* | *CTU1* |
| *LIN28B* | *GTF2H1* | *AKAP5* | *KLRF1* | *CD7* |
| *MAPRE2* | *ENC1* | *CNOT6* | *WNT16* | *NOVA2* |
| *EP300* | *CAST* | *FAM110B* | *ACVR1B* | *ZBTB4* |
| *TTPA* | *SEC23A* | *TULP4* | *TRIM9* | *MMD* |
| *KPNA4* | *NKRF* | *WDR37* | *SLC12A6* | *BAIAP2* |
| *GABBR2* | *GSPT1* | *MAP4K4* | *SERAC1* | *MBD6* |
| *ZFYVE1* | *KDM5B* | *RNGTT* | *DCAF8* | *TINF2* |
| *SMG1* | *DKK2* | *IGF2* | *SMARCC2* | *GABPAP* |
| *ZNF609* | *ZNF592* | *MAPK1* | *ZFP30* | *SMAD7* |
| *MCL1* | *MCC* | *LRRC28* | *BCL2L2* | *FZR1* |
| *FUBP3* | *PMEPA1* | *NEDD4* | *PLXNB1* | *TAF2* |
| *NCMAP* | *PLEKHF2* | *SREK1IP1* | *ARSJ* | *SYT3* |
| *SEMA3C* | *ANKRD28* | *BSDC1* | *GABRE* | *FNBP1* |
| *FTL* | *RAB23* | *ATAD2B* | *TASP1* | *LYRM7* |
| *UBE2R2* | *SOX6* | *PEA15* | *SPON1* | *NFAM1* |
| *TAOK1* | *TENM2* | *TSPYL1* | *CDK18* | *TVP23C* |
| *PPP2CA* | *THSD7A* | *GALNT1* | *ZNF345* | *FBXL16* |
| *DNM3* | *PDE10A* | *ANAPC16* | *BAG5* | *IGFBP5* |
| *RBM7* | *FRS2* | *TOM1L1* | *TCEANC2* | *TFAP4* |
| *FAM53C* | *COL4A3* | *PDGFC* | *KCNA4* | *CLDN6* |
| *EBF1* | *PPP3CB* | *HDAC9* | *ZFP14* | *TMEM94* |
| *CARMIL1* | *MAP3K13* | *CD109* | *SLC7A11* | *GLIS2* |
| *ETV6* | *STK26* | *HES7* | *SHISAL1* | *ZNF554* |
| *MEF2C* | *BTG3* | *ICE1* | *FOXA1* | *PRDM15* |
| *SULT4A1* | *LSM14A* | *VASH2* | *CACNA1I* | *ARVCF* |
| *TGFBR1* | *CNKSR3* | *RGS17* | *LHFPL2* | *CACNA1A* |
| *TRIM33* | *GSG1L* | *HEXIM1* | *XPO7* | *HSP90B1* |
| *IL16* | *FAF2* | *PPP2R1A* | *LAMP1* | *SCAMP4* |
| *MTMR2* | *PLCB4* | *CCT8* | *ZNF148* | *RSL24D1* |
| *ODF1* | *RPP14* | *ASXL1* | *FOSL2* | *TOR1AIP2* |
| *SMG7* | *DIP2C* | *ASL* | *NKAPL* | *FGF4* |
| *WDFY3* | *PAPOLG* | *PSMD7* | *NSD2* | *MTA1* |
| *AUTS2* | *RAD21* | *UPF2* | *OTOR* | *EPHB2* |
| *SAMD12* | *UBE2D3* | *CALB1* | *RAB11FIP5* | *PATZ1* |
| *KDM6B* | *PPM1F* | *PITPNB* | *GCG* | *ARMH3* |
| *PLAGL2* | *MEGF11* | *PPARGC1A* | *PPP6C* | *SMG5* |
| *HNRNPA2B1* | *HAO1* | *H2AZ2* | *YPEL2* | *USH1G* |
| *CSF1R* | *SMC6* | *SLC18A1* | *MTCH2* | *REXO1* |
| *MYCBP* | *SS18L1* | *PLOD2* | *TNNI1* | *TENT4A* |
| *ADAM9* | *PBRM1* | *PNPLA5* | *PLXNA4* | *SHFL* |
| *RNF144A* | *NONO* | *CNTNAP1* | *EFHD1* | *PTPRS* |
| *DNAJB5* | *ZNF367* | *UBTF* | *NME4* | *LDB1* |
| *PDE7A* | *KBTBD6* | *ATG2B* | *CEACAM7* | *KIAA0930* |
| *BPTF* | *PTCH1* | *MTPAP* | *PIANP* | *ZNRF2* |
| *PELI2* | *KCTD1* | *UBFD1* | *CHCHD7* | *EN1* |
| *MAP2* | *MYO6* | *SLC39A8* | *CACNB4* | *CACNB1* |
| *PPAT* | *NCOA4* | *UHRF1BP1* | *BTBD9* | *ANGPT4* |
| *FBXL19* | *MCFD2* | *RER1* | *IMPDH1* | *PNMA8B* |
| *RETREG2* | *RHOA* | *MSTN* | *POU2F2* | *SGTA* |
| *SGPL1* | *COL10A1* | *TMEM30A* | *RBM10* | *PNPLA7* |
| *RFXANK* | *ARID4A* | *ZNF519* | *ELF5* | *SMARCB1* |
| *ALPK3* | *ARHGAP5* | *PAG1* | *TCP11L2* | *UBE2M* |
| *ARID3A* | *ACTR3* | *PDXDC1* | *MLANA* | *RAB5C* |
| *C6orf47* | *SKAP2* | *TLE4* | *NDST1* | *MPRIP* |
| *DPP10* | *RBM47* | *LSM12* | *DNER* | *ERBB2* |
| *RAPGEFL1* | *CELSR2* | *TAB2* | *BCL2* | *EPHA8* |
| *PCNP* | *GOLPH3L* | *TMEM87A* | *SLC6A15* | *STARD3* |
| *CENPV* | *MTURN* | *IFT57* | *SCG3* | *BRD2* |
| *EPC1* | *KCNA3* | *SUPT7L* | *NSUN4* | *HDAC5* |
| *IL6R* | *DTNA* | *ENTPD3* | *NDUFV3* | *LZTS1* |
| *PTPN18* | *SH3PXD2A* | *ZNF281* | *PPP1R13B* | *ISYNA1* |
| *CYP24A1* | *FBXO28* | *PTGFR* | *RAD1* | *SH3BP5L* |
| *MARCHF6* | *SET* | *KHDRBS2* | *DACT1* | *EFHD2* |
| *ATP11C* | *FAM76A* | *LPL* | *HTR7* | *GALNT17* |
| *ARRB1* | *IGSF11* | *UBE2K* | *ZNF512B* | *RCE1* |
| *PI4K2B* | *SENP2* | *DESI2* | *MTX3* | *SSBP3* |
| *ZFP62* | *RNF168* | *DYNC1LI2* | *HIP1* | *ZNF845* |
| *ZSWIM6* | *AGFG2* | *ROBO1* | *PAQR3* | *CTIF* |
| *RIMS4* | *TET3* | *BTF3* | *TRIM6-TRIM34* | *TIMP2* |
| *TSHZ3* | *HMGA2* | *G6PC2* | *RYR2* | *WBP2* |
| *SLC6A17* | *STX16* | *RIMS3* | *EPS8* | *SHC1* |
| *LRFN2* | *SYNGR1* | *LOX* | *RIMS1* | *ZNF423* |
| *MFSD13A* | *TSC22D3* | *SEPTIN7* | *YTHDF3* | *TMCC2* |
| *LACTB* | *TFRC* | *SLC35E3* | *ZBTB5* | *PHB* |
| *BTG2* | *PPP2R2A* | *MGAT3* | *RBM46* | *SLC25A10* |
| *SLC35A4* | *STAU1* | *RAB14* | *NOTCH2NLA* | *FBXL20* |
| *TMEM161B* | *SETX* | *ARHGAP36* | *PANK3* | *FASN* |
| *NPNT* | *MYO5A* | *BACH1* | *IL1RAP* | *RAVER1* |
| *TM9SF3* | *THUMPD1* | *GNA13* | *PI15* | *NLGN2* |
| *GPC4* | *MARCKS* | *FOXO1* | *EDA2R* | *GAL3ST4* |
| *PCGF6* | *STRAP* | *YWHAH* | *GFRA1* | *HOXB8* |
| *DPM2* | *ADCY6* | *M6PR* | *MBTD1* | *FIS1* |
| *CRB2* | *LMO7* | *ASCC2* | *TMEM64* | *NOTCH2* |
| *ZFHX4* | *STK38L* | *DDX52* | *ZNF805* | *MYLK3* |
| *MMRN2* | *SMIM13* | *AKAP10* | *TMBIM1* | *PPTC7* |
| *GEM* | *CDC14B* | *AADAT* | *HS2ST1* | *GNG12* |
| *CFL2* | *JKAMP* | *MTAP* | *SF1* | *NOX4* |
| *ZC3H11A* | *RBMS2* | *IGSF1* | *OR12D3* | *VAPB* |
| *PARP8* | *RBM26* | *TAL1* | *RUFY2* | *ZNF747* |
| *PCGF5* | *COL4A4* | *PCDHGA7* | *XK* | *NCOA7* |
| *KIAA1522* | *VCL* | *GALNT13* | *RNF144B* | *ZFYVE19* |
| *PSTPIP2* | *EIF2S1* | *RNF217* | *FAM193A* | *LENG8* |
| *C17orf58* | *VDAC1* | *FBXO30* | *TUBB4A* | *SNX6* |
| *YWHAE* | *NAA15* | *MTMR4* | *TMC8* | *TNFAIP2* |
| *RAVER2* | *TRPM3* | *PTP4A2* | *ZFP36L1* | *HKDC1* |
| *ZFP91* | *ASIC1* | *STXBP5* | *SLC2A12* | *BCL2L14* |
| *SLC26A3* | *RNF41* | *PCDHGA11* | *SLC36A1* | *FRK* |
| *SCN2B* | *ACVR2B* | *SSH2* | *RANBP2* | *SYNJ2BP* |
| *ZNRF3* | *CSRNP2* | *CERS6* | *TPD52L2* | *VPS35* |
| *IER2* | *NDFIP2* | *SLC6A1* | *ICMT* | *FAM114A2* |
| *VIM* | *CLDN11* | *YWHAQ* | *REEP1* | *BDNF* |
| *FAM131B* | *SEPTIN11* | *ITGAV* | *DNTT* | *GPSM2* |
| *MTOR* | *ERP29* | *FAM199X* | *DHX29* | *VANGL2* |
| *IER3IP1* | *EIF1AX* | *CAPN7* | *MYLK2* | *SRSF11* |
| *FBN2* | *MAP3K8* | *CREBRF* | *THEMIS* | *SAMD4A* |
| *PSMD11* | *NCK1* | *ILRUN* | *VPS13B* | *TMPRSS13* |
| *MTMR3* | *NRP1* | *FBN1* | *LPAR1* | *FGFR2* |
| *C19orf54* | *ZBTB34* | *PCDHGB6* | *TP53RK* | *ZADH2* |
| *ENTPD4* | *SF3B1* | *GAS7* | *IQCE* | *DHRS13* |
| *CEP85* | *PRKACA* | *HSPA9* | *RERG* | *EHD1* |
| *GALNT7* | *ADAT2* | *CPEB3* | *KNOP1* | *ANO6* |
| *CUL3* | *ASAP2* | *RAPH1* | *ADGRE2* | *SERINC1* |
| *HOXA4* | *SPTY2D1* | *WIZ* | *AKT2* | *AJAP1* |
| *PUM2* | *TMEM255A* | *PMPCA* | *TP63* | *ANKRD33B* |
| *RBM20* | *RRP15* | *GRSF1* | *GIT2* | *LAMA5* |
| *ATP2B4* | *PCDHGA1* | *DCAF7* | *THG1L* | *FAM53B* |
| *EIF4EBP1* | *ANKLE2* | *ZDHHC6* | *GABRA4* | *HIPK3* |
| *RCC2* | *SH3BGRL2* | *KERA* | *FZD10* | *SUDS3* |
| *MTHFD2* | *ROCK1* | *EDN1* | *FKBP14* | *KCNH8* |
| *SEC24D* | *KCNMA1* | *COG6* | *ZHX2* | *MPP7* |
| *NHP2* | *DIP2B* | *MINK1* | *MYEF2* | *PRR14L* |
| *FGFR3* | *RNF6* | *TGFB2* | *UBXN4* | *IP6K1* |
| *SYVN1* | *NLGN1* | *FAM102B* | *ADPRH* | *RAB31* |
| *PRRC2C* | *HSPA12A* | *EPN2* | *ARFGEF1* | *UVRAG* |
| *CNOT7* | *KLF9* | *LRPAP1* | *SMARCD1* | *HGH1* |
| *WARS2* | *KCTD12* | *DIXDC1* | *MACIR* | *NCSTN* |
| *RETREG3* | *PAXIP1* | *ANK2* | *ADCY1* | *DTWD1* |
| *KBTBD8* | *SPOP* | *PRPF4B* | *TRIM34* | *VLDLR* |
| *CCND2* | *HNRNPD* | *STRN* | *UTRN* | *CLK1* |
| *ATRX* | *RNF165* | *SPRY3* | *CCND1* | *METAP2* |
| *DPF2* | *RFX3* | *AGBL2* | *DMD* | *FAM114A1* |
| *CDR2L* | *SWAP70* | *PPP3R1* | *POLR1B* | *MAK* |
| *CDC42SE1* | *SPCS2* | *CXXC5* | *ANOS1* | *MYORG* |
| *EMX2* | *SPRED1* | *MGA* | *GBP4* | *AHCYL1* |
| *HINFP* | *NHLH2* | *VIPAS39* | *MRPL19* | *SZT2* |
| *NT5DC1* | *EHF* | *TMEM47* | *TMED7* | *U2AF2* |
| *MBNL1* | *SETD7* | *SLC32A1* | *SMAD2* | *HOXD13* |
| *RBMS3* | *HIF1A* | *VDAC2* | *ZNF503* | *MED22* |
| *SEMA4F* | *HIPK2* | *API5* | *RILPL1* | *TRMT5* |
| *ERI2* | *TMEM170B* | *BAG2* | *PCDH11X* | *MYSM1* |
| *TTPAL* | *RNF20* | *ADAM12* | *MED14* | *CSMD1* |
| *BDH1* | *KIAA0408* | *FHIP1A* | *PTGES3* | *ANGEL2* |
| *SLC4A4* | *PCDHGC3* | *SH3GLB1* | *BORCS7* | *NKX2-5* |
| *EDC3* | *FOXC1* | *GLI2* | *HHIP* | *SLITRK3* |
| *PTBP3* | *ARHGEF7* | *OLFM4* | *SRSF6* | *SPEF2* |
| *MAP2K7* | *NETO2* | *RAD23B* | *ATXN7L1* | *ZNF483* |
| *JARID2* | *SLC1A1* | *TRIM39* | *ME2* | *GABRA2* |
| *COPS7B* | *ERAP1* | *SGCB* | *CYB5R1* | *GSAP* |
| *CYTH1* | *RBM12* | *ATP2A2* | *CDK12* | *MESP1* |
| *ANK3* | *PTPRG* | *ZFYVE9* | *CDKN1A* | *GRK5* |
| *LNPEP* | *PLK2* | *LUZP2* | *CDH17* | *RELN* |
| *KDM7A* | *LTBP1* | *DAZL* | *TK2* | *TGS1* |
| *RNF44* | *BTF3L4* | *TCF7L2* | *BVES* | *TUBB6* |
| *NRAS* | *NRP2* | *OCLN* | *AP3M1* | *TECPR2* |
| *KLHL2* | *DST* | *EIF4E* | *ADAMTS6* | *PLPPR4* |
| *ALG9* | *ITPRID2* | *PRKCA* | *PTCD3* | *NOM1* |
| *SLC7A2* | *FUT6* | *RIPOR2* | *NIT2* | *ACTG1* |
| *TUB* | *MLLT10* | *AMOTL1* | *GRID1* | *RCBTB1* |
| *JADE2* | *FCHO2* | *CBX1* | *IQGAP1* | *VKORC1L1* |
| *TRIM2* | *PAX5* | *CDH13* | *TSPYL4* | *ARHGAP19* |
| *ANKRD12* | *SENP6* | *EBI3* | *KATNIP* | *LSAMP* |
| *YY1* | *BNIP3L* | *SSR3* | *ZNF143* | *ZCCHC2* |
| *SV2A* | *EIF4H* | *PSME4* | *EFNB3* | *CPPED1* |
| *PROX1* | *TNKS2* | *DDN* | *TNFAIP8* | *RPTOR* |
| *TTC7A* | *SPEN* | *CDC25A* | *FAM117B* | *TIMM10B* |
| *KLF12* | *TXLNG* | *ENAH* | *SAMD8* | *ZDHHC3* |
| *SAMD10* | *RC3H1* | *SMIM14* | *KRBOX4* | *CCNG1* |
| *UBE2N* | *PFKFB2* | *DGKG* | *SLC16A9* | *MIDEAS* |
| *DCLK1* | *FBXO38* | *PAPOLB* | *C3orf62* | *NKX2-2* |
| *ATL2* | *KPNA1* | *GOLPH3* | *MTRFR* | *MEAK7* |
| *DUSP6* | *MDFIC* | *GRPEL2* | *CNNM3* | *CERT1* |
| *KMT5C* | *CNOT4* | *DNAJB14* | *KLHL31* | *CEP170B* |
| *PHF21A* | *TRPC3* | *PRR11* | *SFPQ* | *C5orf51* |
| *KLF6* | *CDKN2AIP* | *SLC7A8* | *EIF5B* | *DAZAP1* |
| *EFNB2* | *PIK3C2B* | *YTHDF1* | *PDE6B* | *TADA2A* |
| *DHX33* | *CSNK1G3* | *RND3* | *HLF* | *KDM1B* |
| *AGO3* | *POU2F1* | *HCAR1* | *MACF1* | *PDXK* |
| *HDAC4* | *DCN* | *PLEKHG4B* | *CEACAM1* | *FRA10AC1* |
| *CDX2* | *KIF3A* | *PDLIM5* | *ENSA* | *SIAH2* |
| *NR3C2* | *NUP153* | *ABHD5* | *SMOC1* | *RBM5* |
| *PAPOLA* | *ASH1L* | *GOLGB1* | *CCN4* | *IL15* |
| *WIPF2* | *NMT1* | *RIMBP2* | *CEP41* | *SIRT7* |
| *ZNF624* | *AAK1* | *HNRNPAB* | *FABP2* | *DFFB* |
| *SLC26A6* | *TMEM33* | *PIKFYVE* | *TDG* | *TTL* |
| *RPS6KA1* | *PCDH9* | *SEMA6A* | *TYRP1* | *CTDSP1* |
| *SLC17A6* | *TIMM8A* | *COPS4* | *SLC33A1* | *MAP3K21* |
| *NFYA* | *LRRC8D* | *CCDC88A* | *SYT4* | *RASSF5* |
| *DICER1* | *LRRN3* | *PAPPA* | *GDAP1* | *ABI2* |
| *AMOT* | *NALF2* | *CBLN1* | *SPRED2* | *MCPH1* |
| *ZC3H7B* | *NR2C2* | *DNM1* | *OLFM3* | *EFCAB14* |
| *KLC2* | *TET1* | *BMPR2* | *HIVEP1* | *ESYT2* |
| *CAMK2N1* | *PAIP1* | *NAB1* | *HOXB13* | *SLC25A16* |
| *BIN2* | *DSC2* | *PCLAF* | *PIN4* | *MAU2* |
| *CTDSPL* | *ERCC4* | *MAGT1* | *LMTK2* | *ARID2* |
| *YARS1* | *MIB1* | *MBL2* | *NIBAN1* | *CDKN2B* |
| *RBFOX2* | *B4GALT1* | *INSYN2A* | *FRMPD2* | *MAP2K3* |
| *USP46* | *SNTB2* | *IL10* | *TRDN* | *PPP2R3B* |
| *C16orf72* | *SEC14L2* | *AHCYL2* | *CNKSR2* | *TSGA10* |
| *CHTF8* | *ZNF714* | *CXADR* | *SUPT16H* | *NUDT16L1* |
| *PHF20* | *CCDC6* | *MFSD14B* | *NEK7* | *ST7L* |
| *ANP32E* | *IPO9* | *C1RL* | *KIAA0232* | *GSK3B* |
| *RUNX1* | *IRF2BP2* | *AFDN* | *ASPH* | *MYL2* |
| *BORCS6* | *ST8SIA4* | *IFI44L* | *EXOC1* | *SP3* |
| *CBX7* | *SLC7A6* | *HBS1L* | *LRATD1* | *SLC15A4* |
| *ACHE* | *MME* | *KIF5B* | *GNG2* | *ANKRD10* |
| *STAT3* | *TSC1* | *KIF2A* | *BPHL* | *NEK9* |
| *KCNH7* | *RBM28* | *NR6A1* | *ALPK1* | *CDC6* |
| *TCF20* | *SERTAD2* | *EYA4* | *VCAN* | *IL36G* |
| *ERBB4* | *RAB22A* | *JAM2* | *AGAP4* | *MOSPD2* |
| *UBR5* | *ZSWIM4* | *KITLG* | *FRAS1* | *HAS2* |
| *HIC2* | *HECTD2* | *APLP2* | *PDYN* | *WWC3* |
| *GLCCI1* | *NEGR1* | *POLR2E* | *CDYL* | *GAS1* |
| *NPTX1* | *EPS15* | *LIN9* | *RGL2* | *TFCP2L1* |
| *ZSWIM5* | *KDM3B* | *FHL1* | *LRRTM3* | *CDC73* |
| *RS1* | *RSPO3* | *SYNGAP1* | *MDGA2* | *ABCC9* |
| *RABEP2* | *PSMD10* | *SELENOI* | *SAMD4B* | *REST* |
| *NRG1* | *TNKS* | *TIPARP* | *AFF1* | *NRBP2* |
| *LRP4* | *SNX18* | *ASPHD2* | *STX1B* | *FAM172A* |
| *MED13* | *TSC22D1* | *CYLD* | *DNAJC3* | *ZNF275* |
| *C1orf21* | *TMEM196* | *LEPR* | *RGP1* | *CTPS1* |
| *ACER2* | *BCL11A* | *MED17* | *KLF3* | *ARHGEF18* |
| *MAP3K12* | *PRPF38B* | *GBE1* | *APOB* | *NAT8L* |
| *HECW1* | *TOX* | *WBP1L* | *CRISP1* | *PPFIA3* |
| *SLC17A7* | *RBM27* | *PCDHGA5* | *MTR* | *DNAAF9* |
| *CAMK2G* | *TBC1D15* | *FAM126B* | *CLCN5* | *TRAPPC10* |
| *FOXP1* | *VAMP3* | *HMGN3* | *SLC4A1* | *CACUL1* |
| *PCDH17* | *HNRNPU* | *RALA* | *KLRG1* | *MR1* |
| *ABHD2* | *GABRB3* | *FN1* | *TRAM1L1* | *NUMB* |
| *MLF2* | *OPCML* | *SCAMP1* | *NARS2* | *SLC6A19* |
| *XPO1* | *IGF2BP2* | *GPHN* | *ENPP4* | *NACC2* |
| *PURA* | *PKNOX1* | *PPARD* | *ME3* | *EPHA5* |
| *ABTB1* | *CREB5* | *PTPN12* | *TSTD2* | *RPL28* |
| *PPP2R5C* | *DAG1* | *TTC39A* | *LRRN2* | *D2HGDH* |
| *SP4* | *ITGA6* | *SLC39A6* | *PCBP2* | *SSH1* |
| *SP1* | *YAF2* | *GPATCH8* | *TCHHL1* | *PADI2* |
| *MAP1B* | *IPO5* | *FKTN* | *PLXNA2* | *PRKAA1* |
| *MAF* | *FOXP2* | *MAP2K1* | *USP9X* | *CBX2* |
| *EIF5A2* | *RAD18* | *TNFAIP1* | *CHL1* | *CAMK2D* |
| *STIM2* | *AMMECR1* | *MMP24* | *DPY19L4* | *ZNF19* |
| *VCPIP1* | *C12orf29* | *ZDHHC11* | *SDHAF3* | *RNMT* |
| *NIPBL* | *CCSER2* | *ZNF181* | *PEX12* | *ZNF331* |
| *OTUD4* | *SLC44A1* | *BCORL1* | *GORASP1* | *JCAD* |
| *E2F3* | *FADS1* | *PSIP1* | *ADM* | *FHL3* |
| *B4GALT5* | *CLNS1A* | *IREB2* | *HLTF* | *CERK* |
| *PCDH10* | *MTSS2* | *ITGB3BP* | *ELL2* | *DUSP28* |
| *CLIP2* | *LAPTM4B* | *YPEL5* | *CXCL14* | *LPCAT2* |
| *SHPRH* | *NEDD9* | *EEF1A1* | *THSD4* | *RTN4* |
| *TRAF3* | *RDX* | *SERTAD4* | *ZNF45* | *RNF214* |
| *GPR107* | *SLC35G1* | *SRD5A3* | *ADCY7* | *LZIC* |
| *HOXD10* | *DOCK9* | *SH2B1* | *CYP2U1* | *EXT2* |
| *DLGAP2* | *HOXC4* | *RAB30* | *PCSK2* | *PRCD* |
| *HMGCR* | *SYT7* | *GMPS* | *ZNF100* | *C9orf40* |
| *NBEA* | *KLHL7* | *LIMCH1* | *KIAA1328* | *HBP1* |
| *SNX13* | *BRMS1L* | *RNF26* | *GOLM2* | *TF* |
| *NAP1L5* | *MEMO1* | *EBAG9* | *ADARB1* | *ZNF678* |
| *SEL1L* | *STRBP* | *SNAP25* | *DIO2* | *FAM168A* |
| *CREB1* | *CRKL* | *FOS* | *PDE4A* | *DHFR2* |
| *MED26* | *APP* | *KIT* | *MSR1* | *DHCR7* |
| *NUDT4* | *CXCL12* | *COL11A1* | *NBPF14* | *OTUD3* |
| *CAMTA1* | *MRAS* | *NAA30* | *TAF5* | *PSTPIP1* |
| *TLK2* | *ARIH1* | *ELAPOR2* | *ST18* | *SLC43A2* |
| *ACER3* | *RAI2* | *SEMA4G* | *ARF3* | *ZBTB3* |
| *BNC2* | *LRP1B* | *BBS10* | *ATP8A2* | *KLHL24* |
| *LPP* | *MFSD14A* | *PCDHGB3* | *USP42* | *TEF* |
| *PIGH* | *YTHDF2* | *CORO1C* | *EPHA2* | *SIGLEC1* |
| *AMBN* | *UNC79* | *NXF1* | *EBF2* | *SLC16A7* |
| *ELAVL4* | *DTX4* | *IER5* | *PRDM2* | *EGR3* |
| *PTPN9* | *SLC2A13* | *ZNF529* | *ZNF107* | *VAPA* |
| *MYT1L* | *SINHCAF* | *TANK* | *ABLIM2* | *ZBTB49* |
| *RNF38* | *HSDL2* | *AFAP1L2* | *HOXB4* | *RFC3* |
| *FAM117A* | *CILP* | *NFIA* | *FLT4* | *CTBP1* |
| *BATF3* | *ZNF518A* | *C1QTNF1* | *CARD8* | *HSDL1* |
| *ZNF106* | *METTL8* | *SLC26A7* | *MAOA* | *SECTM1* |
| *COPS2* | *AKTIP* | *PPP6R3* | *OCRL* | *PDE5A* |
| *CREBBP* | *PCDHGA10* | *LAMC2* | *FAM124B* | *CSTF2T* |
| *TANC2* | *MOB1A* | *GHR* | *GNAI2* | *PEAR1* |
| *PRKAR1A* | *RAP1B* | *FOXP4* | *ACTN2* | *ZFTA* |
| *SMAP1* | *DGKE* | *GOPC* | *GFER* | *NT5C3A* |
| *HUNK* | *JDP2* | *PCDHGC5* | *CEBPA* | *TTC21B* |
| *TASOR* | *NHLRC3* | *NTNG2* | *CDCP1* | *CASK* |
| *AFF4* | *NF1* | *ABAT* | *SUB1* | *SUCO* |
| *H3-5* | *AGFG1* | *PLA2G4C* | *VPS26B* | *URGCP* |
| *MATR3* | *GTDC1* | *FAM234B* | *APOBEC3C* | *PLEKHA1* |
| *CBX6* | *VAMP2* | *HNRNPC* | *SYT2* | *MYB* |
| *AGO2* | *EFEMP1* | *ZNF32* | *CMIP* | *ADAM22* |
| *RCOR3* | *CDK19* | *FOXO4* | *SERPINB8* | *ABHD15* |
| *HMGCS1* | *MSANTD2* | *SEC24B* | *FAM20B* | *SUN1* |
| *NDEL1* | *DOP1B* | *NTSR1* | *AGPAT1* | *GOSR1* |
| *MEAF6* | *WDR43* | *RCAN2* | *GAPT* | *CENPH* |
| *ITGB8* | *GNL3L* | *PCDHGC4* | *BCL6* | *NCL* |
| *STYX* | *MAP3K5* | *HAND2* | *OASL* | *SRSF2* |
| *PPP3CA* | *LHX9* | *PPM1E* | *ITGA2* | *ZC3H7A* |
| *DYRK1A* | *PPP3CC* | *PCDHGB2* | *KIF1A* | *PPP1R9A* |
| *IGF1* | *FZD3* | *PTK7* | *KRT10-AS1* | *VXN* |
| *NYAP2* | *SLC8A1* | *HPGD* | *PTPN3* | *MPP2* |
| *TMEM167B* | *CYB561D1* | *TESC* | *TAOK2* | *MDM2* |
| *PDAP1* | *BCLAF1* | *GABRB2* | *MTMR14* | *UBE2J2* |
| *SNAI1* | *DLG5* | *NEFM* | *NPEPPS* | *SMAD9* |
| *PIP4K2B* | *PHLPP2* | *ELAVL1* | *NPTX2* | *DBR1* |
| *ARL1* | *RAP2C* | *ZNF493* | *NABP1* | *DUSP16* |
| *SLC16A14* | *USP38* | *RHOH* | *CNN1* | *GALNT10* |
| *CDC27* | *HERC4* | *PCDHGB5* | *LRBA* | *MRPL30* |
| *EGLN3* | *PRPS2* | *ARL4A* | *UTP25* | *DHFR* |
| *BIN1* | *NFAT5* | *TOPBP1* | *BTRC* | *OLIG2* |
| *PPP2R5E* | *GALC* | *ADGRL1* | *ADGRL2* | *ZNF793* |
| *CBFB* | *FAM120C* | *OVOL2* | *HES2* | *PPARA* |
| *RAP2B* | *HAS3* | *DCAF12* | *BMPER* | *CNR1* |
| *CSTF3* | *SYNCRIP* | *DSTN* | *STARD7* | *CUL1* |
| *MAN1A1* | *HTR2C* | *ARHGAP44* | *PRDM10* | *KBTBD11* |
| *ATP6V1A* | *FANCI* | *STX6* | *RNF220* | *KMT2C* |
| *NAMPT* | *MAP3K2* | *METTL9* | *CD59* | *PXDN* |
| *CMPK1* | *CHFR* | *DYRK2* | *BET1* | *RSU1* |
| *BTBD3* | *TM9SF2* | *NECTIN1* | *KCNC3* | *GLRA3* |
| *CCNE2* | *ARID1A* | *HEATR5B* | *PDP2* | *ZNF280C* |
| *CTDSPL2* | *MGST3* | *THRA* | *USP7* | *USP8* |
| *BCL11B* | *CDH6* | *CIR1* | *SH3KBP1* | *DPH3* |
| *CACHD1* | *NEUROG2* | *NAB2* | *HERC3* | *ANO1* |
| *CAPRIN1* | *ACSL4* | *ARL6IP1* | *RAD51AP1* | *RHOT1* |
| *GLIPR2* | *ELOVL5* | *CASQ1* | *LRRC14* | *RPAP2* |
| *MARCHF5* | *SPCS3* | *G3BP1* | *NCAPG* | *TP53* |
| *AGO1* | *RASGRP2* | *PAK5* | *LZTR1* | *EOGT* |
| *KRAS* | *VWC2* | *PBX3* | *HPCA* | *OPRM1* |
| *ZNHIT6* | *SRGN* | *NTRK3* | *ZNF618* | *HUS1* |
| *CEP170* | *ULK2* | *RBBP4* | *FCHSD2* | *KAT7* |
| *ZNF292* | *NIT1* | *TMEM198* | *FBRSL1* | *DDX3Y* |
| *U2SURP* | *XPOT* | *PDZD2* | *SLC14A1* | *NRIP3* |
| *NAA20* | *IL1R1* | *PRKD3* | *TMEM68* | *GAPVD1* |
| *QKI* | *FLRT3* | *ANKRD16* | *OLFML2B* | *REPS1* |
| *USP15* | *GATAD1* | *FAT1* | *REV1* | *DDIT4L* |
| *CNTN4* | *NEUROD1* | *CEP120* | *TAF12* | *CRX* |
| *MTFR1* | *RGS4* | *DGKB* | *PLEKHG5* | *C21orf91* |
| *CEP350* | *CADPS2* | *CTTN* | *CSTF2* | *WDR48* |
| *BICD2* | *SPAST* | *B3GNT5* | *AP1G1* | *RBM3* |
| *MAP2K4* | *HAUS3* | *RABL3* | *ADAM19* | *TAPBP* |
| *ZEB2* | *HOXA5* | *TM4SF1* | *MS4A1* | *PIGR* |
| *SMC1A* | *KHDRBS1* | *CELF3* | *AMBRA1* | *RBM43* |
| *R3HDM2* | *BHLHB9* | *URM1* | *SIRPA* | *GNG7* |
| *ECT2* | *NEU3* | *RBMX* | *SZRD1* | *CSNK1A1L* |
| *SPIN1* | *CDKL2* | *SPIB* | *SLC2A14* | *TREM1* |
| *ANKH* | *JAZF1* | *REPS2* | *TSPAN9* | *DTWD2* |
| *ADAMTS5* | *SYT1* | *MMS22L* | *FOXE1* | *TBC1D24* |
| *KLF5* | *ETNK1* | *TBC1D10B* | *KLHL18* | *ZNF490* |
| *ARID4B* | *PHTF2* | *ZBTB7A* | *SOBP* | *GLG1* |
| *ZC3H12C* | *NCBP3* | *P2RX7* | *RALGAPB* | *AMER2* |
| *APPBP2* | *YIPF6* | *EFNB1* | *TMEM104* | *SPATA5* |
| *HS3ST3B1* | *HMG20A* | *ZNF302* | *STAM2* | *CISD1* |
| *ZEB1* | *NR4A3* | *ZNF81* | *SNCB* | *DDHD1* |
| *CHORDC1* | *R3HDM1* | *XRN2* | *MKX* | *TPO* |
| *PCDH19* | *CAMSAP2* | *MED13L* | *MYH9* | *NUDT19* |
| *ITCH* | *OBI1* | *POLI* | *NKD1* | *QPCTL* |
| *TNPO1* | *SC5D* | *MAGED2* | *NUBP2* | *GPI* |
| *PEG10* | *SV2B* | *MPZL1* | *SHANK3* | *BPNT2* |
| *EZH2* | *PUM1* | *CPM* | *DLG1* | *KCNC1* |
| *CAPZA2* | *HECW2* | *LASP1* | *SETD1B* | *ZNF772* |
| *TRIP12* | *MAPK1IP1L* | *EFNA5* | *NUTM2G* | *NOL9* |
| *RERE* | *PTBP1* | *SLC12A2* | *ZBED2* | *PPFIBP1* |
| *IRS1* | *DCAF10* | *SLC25A13* | *NT5DC3* | *TMEM220* |
| *KCMF1* | *TC2N* | *EDNRB* | *AK4* | *BTN3A1* |
| *C15orf48* | *STK4* | *IGFBP1* | *FAM104B* | *CLCN4* |
| *TCERG1* | *PHLDA1* | *LIMA1* | *ADGRG2* | *TTF2* |
| *MYLK* | *CD28* | *PCDHA4* | *AADACL3* | *RLIM* |
| *LARP4B* | *CD53* | *ZHX1* | *NSL1* | *ZBTB24* |
| *FZD8* | *CNTNAP2* | *ZBTB33* | *KRTAP1-1* | *RAB3B* |
| *FBXL5* | *LDLRAD4* | *RAPGEF2* | *ATP7A* | *TINAG* |
| *SMAD4* | *ST8SIA3* | *SLC4A8* | *ZNF571* | *GDF6* |
| *VCP* | *CPNE8* | *TSKU* | *COL5A1* | *SMARCA4* |
| *MEF2A* | *TNFAIP8L3* | *ZNF652* | *MAP3K1* | *ITGA3* |
| *OLA1* | *NPTN* | *NLGN4Y* | *EXPH5* | *SUGT1* |
| *SULF1* | *C2orf69* | *DNAJB6* | *FANCA* | *WWTR1* |
| *CCNT2* | *GLDN* | *CACNA1C* | *DUSP8* | *RNF24* |
| *ADNP* | *SEPTIN6* | *GRHL1* | *RNF146* | *MBOAT1* |
| *HOXA3* | *ELOVL7* | *RTN1* | *HOXC6* | *LYZ* |
| *TNRC6A* | *RAB6A* | *BCAP29* | *UST* | *IRAK3* |
| *ZDHHC17* | *AKAP9* | *CHP1* | *C12orf4* | *OGFOD3* |
| *RNF216* | *CEBPG* | *PCDHB11* | *ITPRID1* | *LILRA1* |
| *ACVR1* | *USP3* | *HEG1* | *C5orf22* | *MAVS* |
| *DPP8* | *RSRP1* | *EGR1* | *TMBIM6* | *DNAAF5* |
| *VPS36* | *SLC30A4* | *USP4* | *RALGPS1* | *G6PC1* |
| *GIGYF2* | *PHACTR2* | *PRR14* | *MBP* | *QTRT2* |
| *AGPAT5* | *PCDHGA12* | *SPARC* | *FRMPD4* | *OAS2* |
| *ENOX2* | *KMT2D* | *XYLT1* | *ZNF420* | *NUP43* |
| *LMX1A* | *NRF1* | *GOT2* | *RARA* | *FDXACB1* |
| *NCKAP1* | *RUBCN* | *KHDRBS3* | *ST6GALNAC4* | *SALL3* |
| *ZNF608* | *SPTBN1* | *QSER1* | *JAK2* | *ZNF548* |
| *CREBZF* | *RAP1A* | *PCDH11Y* | *KANSL1* | *MOB3A* |
| *SMARCA2* | *NCOA3* | *KCNE1* | *NAV2* | *PDPN* |
| *LONRF1* | *WDR26* | *FASLG* | *RBM33* | *ZDBF2* |
| *UBQLN2* | *DENND2B* | *APLNR* | *SGSM2* | *SERPINB9* |
| *USP34* | *EML6* | *SRGAP3* | *JOSD1* | *ARSD* |
| *TAF4* | *ATP11A* | *TOMM7* | *ECHDC1* | *SPATS2L* |
| *SRSF1* | *DENND5B* | *TACC2* | *CAPN1* | *NANOG* |
| *SLC4A7* | *EPHA7* | *ANKIB1* | *NCAM1* | *WHAMM* |
| *UNKL* | *PUS7* | *PAX3* | *SCRN1* | *SPRING1* |
| *HS3ST2* | *POU3F2* | *NMNAT2* | *FOXJ2* | *PLEKHA5* |
| *CUL5* | *BAZ1A* | *SLC20A2* | *GOLGA3* | *GSTZ1* |
| *ADCY2* | *SGK1* | *ARHGEF9* | *PRAME* | *UTP15* |
| *ACTB* | *PPM1B* | *TSPAN12* | *MAPK7* | *C17orf75* |
| *MAP7* | *RASGRP3* | *XPR1* | *IQSEC2* | *ZNF549* |
| *RIMS2* | *INSR* | *SDHC* | *WNK3* | *PCBD2* |
| *DACH1* | *KCNJ2* | *XKR6* | *ARHGAP11A* | *DAND5* |
| *PRRG1* | *GPRC5A* | *LAMB4* | *SLC35F6* | *FLCN* |
| *USP44* | *MCM2* | *UBR3* | *ZNF587B* | *ACVR1C* |
| *DDAH1* | *GINS1* |  |  |  |

STable 4: List of Human Genes Input into STRING

| Genes in Common Between Human Putative Target Genes and Cell Cycle | | | |
| --- | --- | --- | --- |
| *AKT3* | *EP300* | *ORC2* | *RCC2* |
| *AHCTF1* | *FBXL18* | *PAFAH1B1* | *RFC1* |
| *AKAP9* | *FZR1* | *PDS5A* | *RFC3* |
| *AKT2* | *GINS1* | *PHF20* | *RNF168* |
| *ANAPC16* | *GORASP1* | *PHF8* | *SET* |
| *ANKLE2* | *GSK3B* | *PHLDA1* | *SMARCA5* |
| *ANKRD28* | *H2AZ2* | *POLD3* | *SPAST* |
| *ATRX* | *HAUS3* | *POLE3* | *SRC* |
| *BRCA1* | *HUS1* | *POLR2E* | *STAG1* |
| *BTRC* | *IST1* | *PPP1CB* | *STAG2* |
| *CCND1* | *ITGB3BP* | *PPP1CC* | *SUMO1* |
| *CCND2* | *JAK2* | *PPP1R12B* | *SUN1* |
| *CCNE2* | *KIF2A* | *PPP2CA* | *SUN2* |
| *CDC25A* | *LBR* | *PPP2CB* | *SYNE1* |
| *CDC27* | *LEMD3* | *PPP2R1A* | *TAOK1* |
| *CDC6* | *LIN9* | *PPP2R1B* | *TFDP2* |
| *CDK2* | *LMNA* | *PPP2R2A* | *TINF2* |
| *CDK6* | *LPIN2* | *PPP2R3B* | *TNPO1* |
| *CDKN1A* | *MAPK1* | *PPP2R5C* | *TOPBP1* |
| *CDKN2B* | *MAU2* | *PPP2R5E* | *TP53* |
| *CENPH* | *MAX* | *PPP6C* | *TUBB4A* |
| *CENPK* | *MCM2* | *PPP6R3* | *TUBB6* |
| *CEP41* | *MCPH1* | *PRKACA* | *UBE2D1* |
| *CHMP2B* | *MDM2* | *PRKAR2B* | *UBE2N* |
| *CHTF8* | *NCAPG* | *PRKCA* | *WAPL* |
| *CKS1B* | *NDEL1* | *PRKCB* | *XPO1* |
| *CSNK2A1* | *NEK7* | *PSMD10* | *YWHAE* |
| *CUL1* | *NEK9* | *PSMD11* | *YWHAG* |
| *DHFR* | *NHP2* | *PSMD7* | *YWHAH* |
| *DYNC1LI2* | *NIPBL* | *PSME4* | *YWHAQ* |
| *DYNLL2* | *NSD2* | *RAB1B* | *YWHAZ* |
| *DYRK1A* | *NSL1* | *RAD1* | *ZNF385A* |
| *E2F1* | *NUP153* | *RAD21* | *ZWINT* |
| *E2F2* | *NUP210* | *RAN* |  |
| *E2F3* | *NUP43* | *RANBP2* |  |
| *ENSA* | *NUP62* | *RBBP4* |  |

STable 5: List of Genes in Common Between Human Putative Target Genes, Upregulated Mouse Genes at 2 Days Post-PH, and Cell Cycle Genes

| 46 Genes in Common Between Human Putative Target Genes, Upregulated Mouse Genes 2-Days Post-PH and Cell Cycle Genes | | | | |
| --- | --- | --- | --- | --- |
| *ORC2* | *E2F3* | *CDC25A* | *RAN* | *CDK2* |
| *YWHAZ* | *CDC27* | *POLR2E* | *E2F1* | *E2F2* |
| *SMARCA5* | *CCNE2* | *LIN9* | *RANBP2* | *TINF2* |
| *YWHAE* | *LBR* | *ITGB3BP* | *CCND1* | *TUBB6* |
| *RCC2* | *CENPK* | *TOPBP1* | *CDKN1A* | *MCPH1* |
| *NHP2* | *BRCA1* | *SUN2* | *NCAPG* | *CDC6* |
| *CHTF8* | *RAD21* | *POLE3* | *NSL1* | *RFC3* |
| *XPO1* | *MCM2* | *TUBB2A* | *CKS1B* | *CENPH* |
| *YWHAH* | *GINS1* | *NUP43* | *POLD3* | *MDM2* |
| *DHFR* |  |  |  |  |
| 243 Genes in Common Between Human Putative Target Genes and Upregulated Mouse Genes 2-Days Post-PH | | | | |
| *SUV39H1* | *CALM2* | *KHDRBS1* | *PSIP1* | *ATF3* |
| *SLC2A1* | *RPS6KA3* | *NEU3* | *GMPS* | *ZIK1* |
| *ETF1* | *TMEM263* | *PTBP1* | *CORO1C* | *FAM98A* |
| *SRSF10* | *BTAF1* | *TC2N* | *NAB2* | *SDR39U1* |
| *ARF6* | *G3BP2* | *ELOVL7* | *ARL6IP1* | *TPD52* |
| *PRRC1* | *MYH10* | *RAB6A* | *G3BP1* | *CALM3* |
| *USP37* | *XPO4* | *SLC30A4* | *FAT1* | *KLHDC3* |
| *NECAP1* | *SLC5A3* | *SSR1* | *RBMX* | *LANCL2* |
| *MEGF9* | *PAFAH1B2* | *TBC1D19* | *MMS22L* | *SEMA4B* |
| *FNDC3B* | *FNBP1L* | *CRIM1* | *TSKU* | *VANGL1* |
| *ETV6* | *GRIA3* | *SLC16A1* | *GRHL1* | *USP22* |
| *MTMR2* | *LANCL1* | *CBX5* | *EGR1* | *ANO10* |
| *PPAT* | *CCNC* | *POLH* | *SPARC* | *TAF2* |
| *MTHFD2* | *LRRC8B* | *ENTPD7* | *TGIF1* | *UBE2M* |
| *ERI2* | *FAM168B* | *MOB3B* | *WDR36* | *PPTC7* |
| *TRIM2* | *BZW1* | *SEC23IP* | *SMYD5* | *GPSM2* |
| *KLF6* | *SDAD1* | *RP2* | *PPIL1* | *ZADH2* |
| *NFYA* | *TMEM65* | *GOLT1B* | *NPAT* | *NCSTN* |
| *ANP32E* | *SRSF7* | *ILF3* | *TGFBR2* | *ARHGAP19* |
| *NRG1* | *PPM1D* | *ARFGAP3* | *HDAC2* | *CCNG1* |
| *ABHD2* | *CAST* | *CEP55* | *CRTAP* | *RPL28* |
| *MLF2* | *NKRF* | *HSPA13* | *DEF8* | *LZIC* |
| *B4GALT5* | *BTG3* | *BTBD10* | *CARNMT1* | *SUCO* |
| *HUNK* | *KBTBD6* | *USP1* | *SEC23B* | *SRSF2* |
| *AGO2* | *IGSF11* | *SLC1A4* | *FBXO48* | *ZC3H7A* |
| *EGLN3* | *TFRC* | *CYCS* | *ARHGDIA* | *MRPL30* |
| *CBFB* | *RBM26* | *TMED5* | *UBAP2* | *EOGT* |
| *CSTF3* | *EIF2S1* | *CAPZA1* | *PMP22* | *RBM3* |
| *CMPK1* | *NAA15* | *UCHL5* | *SH3BGRL* | *QPCTL* |
| *CTDSPL2* | *NDFIP2* | *PKM* | *EMP1* | *TTF2* |
| *KRAS* | *NETO2* | *CNOT6* | *COQ7* | *SUGT1* |
| *CEP170* | *PFKFB2* | *MAP4K4* | *RRN3* | *WWTR1* |
| *U2SURP* | *KPNA1* | *LRRC28* | *PPP1R13B* | *UTP15* |
| *NAA20* | *AAK1* | *SUPT7L* | *GFRA1* | *ARHGAP11A* |
| *ECT2* | *IPO9* | *DESI2* | *FKBP14* | *SLC35F6* |
| *CHORDC1* | *HECTD2* | *M6PR* | *MYEF2* | *ADM* |
| *EZH2* | *VAMP3* | *DDX52* | *MRPL19* | *LAPTM4B* |
| *TCERG1* | *IGF2BP2* | *MTAP* | *MED14* | *PTCD3* |
| *MEMO1* | *RAD18* | *CERS6* |  |  |
| 204 Genes in Common Between Cell Cycle and Upregulated Mouse Genes 2-Days Post-PH | | | | |
| *ERCC6L* | *CENPN* | *MCM6* | *NCAPD3* | *PRIM2* |
| *RANGAP1* | *DYNC1I2* | *MCM7* | *SMC2* | *RRM2* |
| *PLK1* | *KIF2C* | *MCM3* | *SMC4* | *TYMS* |
| *MAPRE1* | *CKAP5* | *MCM5* | *NCAPH2* | *PCNA* |
| *CENPE* | *NDE1* | *CDC45* | *NCAPG2* | *RBL1* |
| *KIF18A* | *SPDL1* | *CLSPN* | *NCAPH* | *CDKN2D* |
| *CDC20* | *UBE2C* | *CCNE1* | *NCAPD2* | *CDKN2C* |
| *BUB1* | *ANAPC4* | *CCNA2* | *TUBG1* | *CCND3* |
| *SKA2* | *CDC23* | *TERF2* | *TUBGCP2* | *TOP2A* |
| *SKA1* | *CHEK2* | *LMNB1* | *CEP57* | *AURKA* |
| *CENPF* | *BARD1* | *BRCA2* | *ACTR1A* | *ESCO2* |
| *MAD2L1* | *NBN* | *RAD51* | *CNTRL* | *POLD1* |
| *BUB3* | *MDC1* | *RAD51C* | *HSP90AA1* | *POLD2* |
| *BUB1B* | *RFC2* | *FBXO5* | *CCP110* | *POLD4* |
| *NUP98* | *RFC5* | *ESPL1* | *NEK2* | *LIG1* |
| *NUP107* | *RFC4* | *TUBB4B* | *HAUS4* | *FEN1* |
| *NUP160* | *RHNO1* | *TUBA8* | *HAUS7* | *GINS4* |
| *NUP37* | *RAD9A* | *TUBA1B* | *HAUS1* | *GINS3* |
| *NUP133* | *BRIP1* | *TUBA1C* | *HAUS8* | *GINS2* |
| *SEC13* | *BLM* | *CDCA5* | *HAUS5* | *CDC25B* |
| *KNTC1* | *EXO1* | *VRK1* | *HAUS6* | *BORA* |
| *ZWILCH* | *RBBP8* | *BANF1* | *CEP72* | *AJUBA* |
| *NUF2* | *RPA1* | *KPNB1* | *OFD1* | *TPX2* |
| *SPC24* | *RPA2* | *TMPO* | *CEP192* | *HMMR* |
| *NDC80* | *ATR* | *EMD* | *CEP135* | *FOXM1* |
| *SPC25* | *CHEK1* | *POM121* | *CEP290* | *PPME1* |
| *MIS12* | *CDC25C* | *NUP93* | *PLK4* | *MIS18A* |
| *DSN1* | *WEE1* | *NUP35* | *CEP76* | *MIS18BP1* |
| *PMF1* | *CCNB1* | *NUP155* | *CDK5RAP2* | *OIP5* |
| *BIRC5* | *CDK1* | *NUP205* | *EML4* | *CENPW* |
| *AURKB* | *PSMD14* | *NDC1* | *SKP2* | *RBBP7* |
| *INCENP* | *PSMB2* | *KIF20A* | *TFDP1* | *HJURP* |
| *CDCA8* | *GTSE1* | *KIF23* | *LIN54* | *NPM1* |
| *CENPA* | *PKMYT1* | *MASTL* | *E2F4* | *DSCC1* |
| *CENPT* | *CCNB2* | *PPP2R2D* | *E2F5* | *CHTF18* |
| *CENPI* | *DBF4* | *NEK6* | *POLE2* | *CTC1* |
| *CENPP* | *CDC7* | *CNEP1R1* | *POLE* | *RTEL1* |
| *CENPU* | *ORC4* | *AAAS* | *GMNN* | *GAR1* |
| *CENPQ* | *MCM8* | *NUP50* | *CDT1* | *PIF1* |
| *CENPM* | *ORC6* | *NUP88* | *POLA1* | *POLR2D* |
| *TPR* | *MCM4* | *POLR2F* | *PRIM1* |  |

STable 6: List of Genes in Common Between Human Putative Target Genes, Downregulated Mouse Genes at 7 Days Post-PH, and Cell Cycle Genes

| 2 Genes in Common Between Human Putative Target Genes, Downregulated Mouse Genes 7-Days Post-PH and Cell Cycle Genes | | | | |
| --- | --- | --- | --- | --- |
| *SMC1A* | *TUBB2A* |  |  |  |
| 243 Genes in Common Between Human Putative Target Genes and Downregulated Mouse Genes 7-Days Post-PH | | | | |
| SYNE1 | PPP2R5C | PHLDA1 | SUN2 | RAB1B |
| STAG2 | NIPBL | AKAP9 | LEMD3 | CDK2 |
| AKT3 | E2F3 | MCM2 | UBE2D1 | LMNA |
| PHF8 | NDEL1 | PRKCB | POLE3 | DYNLL2 |
| MAX | DYRK1A | YWHAG | STAG1 | E2F2 |
| IST1 | CDC27 | PPP2CB | ZWINT | FBXL18 |
| CDK6 | PPP2R5E | SUMO1 | RAN | TINF2 |
| ORC2 | CCNE2 | AHCTF1 | E2F1 | FZR1 |
| YWHAZ | TNPO1 | PPP2R1B | NSD2 | TUBB6 |
| SMARCA5 | LBR | PPP1R12B | PPP6C | MCPH1 |
| PDS5A | PPP1CB | MAPK1 | RAD1 | MAU2 |
| NUP210 | CENPK | ANAPC16 | TUBB4A | CDKN2B |
| CSNK2A1 | RFC1 | PPP2R1A | RANBP2 | PPP2R3B |
| PAFAH1B1 | PRKAR2B | PSMD7 | AKT2 | GSK3B |
| EP300 | PPP1CC | H2AZ2 | CCND1 | NEK9 |
| TAOK1 | TFDP2 | DYNC1LI2 | CDKN1A | CDC6 |
| PPP2CA | LPIN2 | YWHAH | ENSA | RFC3 |
| YWHAE | BRCA1 | YWHAQ | CEP41 | SUN1 |
| PSMD11 | CHMP2B | PRKCA | NEK7 | CENPH |
| RCC2 | ANKRD28 | PSME4 | GORASP1 | MDM2 |
| NHP2 | RAD21 | CDC25A | BTRC | DHFR |
| CCND2 | SET | KIF2A | NCAPG | CUL1 |
| ATRX | RNF168 | POLR2E | NSL1 | TP53 |
| UBE2N | PPP2R2A | LIN9 | JAK2 | HUS1 |
| CHTF8 | PRKACA | ITGB3BP | CKS1B | NUP43 |
| PHF20 | ANKLE2 | PPP6R3 | SRC | GINS1 |
| XPO1 | NUP153 | TOPBP1 | POLD3 | NUP62 |
| HAUS3 | PSMD10 | RBBP4 | ZNF385A | SPAST |
| WAPL |  |  |  |  |
| 16 Genes in Common Between Human Putative Target Genes and Downregulated Mouse Genes 7-Days Post-PH | | | | |
| *STARD13* | *GABBR2* | *RAI14* | *EHBP1* | *ATXN7L1* |
| *RIC8B* | *CPEB2* | *DAPK1* | *TAB2* | *USP7* |
| *BACH2* | *CPEB4* | *ATP11A* | *RAPH1* | *MYSM1* |
| *DDHD1* |  |  |  |  |

STable 7: Putative Tissue Source from Human Plasma MicroRNA

| MicroRNA | Sequence (5′→3′) | | Liver Cirrhosis |
| --- | --- | --- | --- |
| *hsa-miR-1255a* | | AGGAUGAGCAAAGAAAGUAGAUU | x |
| *hsa-miR-1258* | | AGUUAGGAUUAGGUCGUGGAA | ✓ |
| *hsa-miR-125a-3p* | | ACAGGUGAGGUUCUUGGGAGCC | ✓ |
| *hsa-miR-125a-5p* | | UCCCUGAGACCCUUUAACCUGUGA | ✓ |
| *hsa-miR-1275* | | GUGGGGGAGAGGCUGUC | ✓ |
| *hsa-miR-1278* | | UAGUACUGUGCAUAUCAUCUAU | ✓ |
| *hsa-miR-1303* | | UUUAGAGACGGGGUCUUGCUCU | ✓ |
| *hsa-miR-197-3p* | | UUCACCACCUUCUCCACCCAGC | ✓ |
| *hsa-miR-219a-1-3p* | | AGAGUUGAGUCUGGACGUCCCG | ✓ |
| *hsa-miR-22-3p* | | AAGCUGCCAGUUGAAGAACUGU | ✓ |
| *hsa-miR-28-3p* | | CACUAGAUUGUGAGCUCCUGGA | ✓ |
| *hsa-miR-328-5p* | | GGGGGGGCAGGAGGGGCUCAGGG | x |
| *hsa-miR-4286* | | ACCCCACUCCUGGUACC | ✓ |
| *hsa-miR-433-5p* | | UACGGUGAGCCUGUCAUUAUUC | x |
| *hsa-miR-450b-5p* | | UUUUGCAAUAUGUUCCUGAAUA | ✓ |
| *hsa-miR-502-3p* | | AAUGCACCUGGGCAAGGAUUCA | ✓ |
| *hsa-miR-523-3p* | | GAACGCGCUUCCCUAUAGAGGGU | x |
| *hsa-miR-548b-3p* | | CAAGAACCUCAGUUGCUUUUGU | ✓ |
| *hsa-miR-615-3p* | | UCCGAGCCUGGGUCUCCCUCUU | x |
| *hsa-miR-620* | | AUGGAGAUAGAUAUAGAAAU | x |
| *hsa-miR-660-5p* | | UACCCAUUGCAUAUCGGAGUUG | ✓ |
| *hsa-miR-875-3p* | | CCUGGAAACACUGAGGUUGUG | x |
| *hsa-miR-892a* | | CACUGUGUCCUUUCUGCGUAG | x |
| *hsa-miR-942-3p* | | CACAUGGCCGAAACAGAGAAGU | x |
| *hsa-miR-99b-5p* | | CACCCGUAGAACCGACCUUGCG | ✓ |

STable 8: Putative Tissue Source of Murine MicroRNAs

| MicroRNA | Sequence (5′→3′) | | Liver Cirrhosis |
| --- | --- | --- | --- |
| *hsa-miR-101-2-5p* | | UCGGUUAUCAUGGUACCGAUGC | n/a |
| *hsa-miR-125a-3p* | | ACAGGUGAGGUUCUUGGGAGCC | ✓ |
| *hsa-miR-133b* | | UUUGGUCCCCUUCAACCAGCUA | ✓ |
| *hsa-miR-146b-5p* | | UGAGAACUGAAUUCCAUAGGCUG | ✓ |
| *hsa-miR-188-5p* | | CAUCCCUUGCAUGGUGGAGGG | ✓ |
| *hsa-miR-18b-5p* | | UAAGGUGCAUCUAGUGCAGUUAG | ✓ |
| *hsa-miR-190b-5p* | | UGAUAUGUUUGAUAUUGGGUUG | ✓ |
| *hsa-miR-192-3p* | | CUGCCAAUUCCAUAGGUCACAG | ✓ |
| *hsa-miR-192-5p* | | CUGACCUAUGAAUUGACAGCC | ✓ |
| *hsa-miR-196b-5p* | | UAGGUAGUUUCCUGUUGUUGGG | ✓ |
| *hsa-miR-204-3p* | | GCUGGGAAGGCAAAGGGACGU | x |
| *hsa-miR-204-5p* | | UUCCCUUUGUCAUCCUAUGCCU | ✓ |
| *hsa-miR-31-3p* | | UGCUAUGCCAACAUAUUGCCAU | x |
| *hsa-miR-31-5p* | | AGGCAAGAUGCUGGCAUAGCU | ✓ |
| *hsa-miR-340-5p* | | UUAUAAAGCAAUGAGACUGAUU | ✓ |
| *hsa-miR-34b-5p* | | UAGGCAGUGUCAUUAGCUGAUUG | x |
| *hsa-miR-377-3p* | | AUCACACAAAGGCAACUUUUGU | ✓ |
| *hsa-miR-377-5p* | | AGAGGUUGCCCUUGGUGAAUUC | x |
| *hsa-miR-431-3p* | | CAGGUCGUCUUGCAGGGCUUCU | x |
| *hsa-miR-431-5p* | | UGUCUUGCAGGCCGUCAUGCA | ✓ |
| *hsa-miR-450b-5p* | | UUUUGCAAUAUGUUCCUGAAUA | ✓ |
| *hsa-miR-485-5p* | | AGAGGCUGGCCGUGAUGAAUUC | ✓ |
| *hsa-miR-568* | | AUGUAUAAAUGUAUACACAC | x |
| *hsa-miR-767-5p* | | UGCACCAUGGUUGUCUGAGCAUG | x |

STable 9: Key MicroRNAs Identified in Murine NanoString Analysis

| MicroRNA | Fold Change | *p value* | Human MicroRNA Homologs |
| --- | --- | --- | --- |
| *mmu-miR-1941-3p* | -2.5 | 0.01888 | n/a |
| *mmu-miR-192* | -2.34 | 0.02552 | *hsa-miR-192-5p,*  *hsa-miR-192-3p* |
| *mmu-miR-453* | -1.97 | 0.03685 | n/a |
| *mmu-miR-188-5p* | -1.95 | 0.01755 | *hsa-miR-188-5p* |
| *mmu-miR-679* | -1.94 | 0.01704 | n/a |
| *mghv-miR-M1-7-3p* | -1.84 | 0.02372 | n/a |
| *mmu-miR-767* | -1.8 | 0.01743 | *hsa-miR-767-5p* |
| *mmu-miR-196b* | -1.74 | 0.02999 | [*hsa-miR-196b-5p*](https://mirbase.org/hairpin/MIMAT0026557) |
| *mmu-miR-377* | -1.56 | 0.00307 | *hsa-miR-377-5p,*  *hsa-miR-377-3p* |
| *mmu-miR-741* | -1.51 | 0.00532 | n/a |
| *mmu-miR-872* | -1.48 | 0.03295 | n/a |
| *mmu-miR-1946b* | -1.46 | 0.02546 | n/a |
| *mmu-miR-101b* | -1.46 | 0.03189 | *hsa-miR-101-2-5p* |
| *mmu-miR-190* | -1.45 | 0.0039 | *hsa-miR-190b-5p* |
| *mmu-miR-450b-5p* | -1.44 | 0.04801 | *hsa-miR-450b-5p* |
| *mmu-miR-485* | -1.41 | 0.01075 | *hsa-miR-485-5p* |
| *mmu-miR-568* | -1.38 | 0.00176 | *hsa-miR-568* |
| *mmu-miR-34b-5p* | -1.31 | 0.0257 | *hsa-miR-34b-5p* |
| *mmu-miR-31* | -1.31 | 0.04956 | *hsa-miR-31-5p,*  *hsa-miR-31-3p* |
| *mmu-miR-431* | 1.29 | 0.0457 | *hsa-miR-431-5p,*  *hsa-miR-431-3p* |
| *mmu-miR-125a-3p* | 1.43 | 0.00887 | *hsa-miR-125a-3p* |
| *mmu-miR-3475* | 1.54 | 0.03871 | n/a |
| *mmu-miR-133b* | 1.55 | 0.02448 | *hsa-mir-133b* precursor |
| *mmu-miR-297b-3p* | 1.56 | 0.01484 | n/a |
| *mmu-miR-204* | 1.64 | 0.04177 | *hsa-miR-204-5p,*  *hsa-miR-204-3p* |
| *mmu-miR-146b* | 1.66 | 0.0093 | *hsa-miR-146b-5p* |
| *mmu-miR-293* | 1.69 | 0.0486 | n/a |
| *mmu-miR-18b* | 2 | 0.02921 | *hsa-miR-18b-5p* |
| *mmu-miR-340-5p* | 2.03 | 0.02576 | *hsa-miR-340-5p* |
| *mmu-miR-450a-3p* | 2.06 | 0.02492 | n/a |

STable 10: List of All Murine MiRNA Found in Circulating Small EVs from NanoString

| 596 Significant MiRNAs Found in Mouse Small EVs | | | | |
| --- | --- | --- | --- | --- |
| *mcmv-miR-M23-1-3p* | *mmu-miR-182* | *mmu-miR-2139* | *mmu-miR-375* | *mmu-miR-615-5p* |
| *mcmv-miR-M23-1-5p* | *mmu-miR-183* | *mmu-miR-214* | *mmu-miR-376a* | *mmu-miR-652* |
| *mcmv-miR-M23-2* | *mmu-miR-1839-3p* | *mmu-miR-2140* | *mmu-miR-376b* | *mmu-miR-653* |
| *mcmv-miR-M44-1* | *mmu-miR-1839-5p* | *mmu-miR-2141* | *mmu-miR-376c* | *mmu-miR-654-3p* |
| *mcmv-miR-M55-1* | *mmu-miR-184* | *mmu-miR-2145* | *mmu-miR-377* | *mmu-miR-654-5p* |
| *mcmv-miR-M87-1* | *mmu-miR-185* | *mmu-miR-2146* | *mmu-miR-378* | *mmu-miR-664* |
| *mcmv-miR-M95-1-3p* | *mmu-miR-186* | *mmu-miR-216a* | *mmu-miR-379* | *mmu-miR-665* |
| *mcmv-miR-M95-1-5p* | *mmu-miR-187* | *mmu-miR-216b* | *mmu-miR-380-3p* | *mmu-miR-666-3p* |
| *mcmv-miR-m01-1* | *mmu-miR-188-3p* | *mmu-miR-217* | *mmu-miR-380-5p* | *mmu-miR-666-5p* |
| *mcmv-miR-m01-2* | *mmu-miR-188-5p* | *mmu-miR-218* | *mmu-miR-381* | *mmu-miR-667* |
| *mcmv-miR-m01-3* | *mmu-miR-1892* | *mmu-miR-2182* | *mmu-miR-382* | *mmu-miR-668* |
| *mcmv-miR-m01-4* | *mmu-miR-1893* | *mmu-miR-2183* | *mmu-miR-383* | *mmu-miR-669a* |
| *mcmv-miR-m107-1-3p* | *mmu-miR-1894-3p* | *mmu-miR-219* | *mmu-miR-384-3p* | *mmu-miR-669e* |
| *mcmv-miR-m107-1-5p* | *mmu-miR-1894-5p* | *mmu-miR-22* | *mmu-miR-384-5p* | *mmu-miR-669f* |
| *mcmv-miR-m108-1* | *mmu-miR-1895* | *mmu-miR-220* | *mmu-miR-409-3p* | *mmu-miR-669g* |
| *mcmv-miR-m108-2-3p* | *mmu-miR-1896* | *mmu-miR-221* | *mmu-miR-409-5p* | *mmu-miR-669h-5p* |
| *mcmv-miR-m108-2-5p.1* | *mmu-miR-1897-3p* | *mmu-miR-222* | *mmu-miR-410* | *mmu-miR-669i* |
| *mcmv-miR-m108-2-5p.2* | *mmu-miR-1898* | *mmu-miR-223* | *mmu-miR-411* | *mmu-miR-669j* |
| *mcmv-miR-m21-1* | *mmu-miR-1899* | *mmu-miR-224* | *mmu-miR-412* | *mmu-miR-669m* |
| *mcmv-miR-m22-1* | *mmu-miR-18a* | *mmu-miR-23a* | *mmu-miR-421* | *mmu-miR-669o* |
| *mcmv-miR-m59-1* | *mmu-miR-18b* | *mmu-miR-23b* | *mmu-miR-423-3p* | *mmu-miR-670* |
| *mcmv-miR-m59-2* | *mmu-miR-190* | *mmu-miR-24* | *mmu-miR-423-5p* | *mmu-miR-671-3p* |
| *mcmv-miR-m88-1* | *mmu-miR-1900* | *mmu-miR-25* | *mmu-miR-425* | *mmu-miR-671-5p* |
| *mghv-miR-M1-1* | *mmu-miR-1901* | *mmu-miR-26a* | *mmu-miR-429* | *mmu-miR-672* |
| *mghv-miR-M1-2* | *mmu-miR-1902* | *mmu-miR-26b* | *mmu-miR-431* | *mmu-miR-673-3p* |
| *mghv-miR-M1-3* | *mmu-miR-1903* | *mmu-miR-27a* | *mmu-miR-432* | *mmu-miR-673-5p* |
| *mghv-miR-M1-4* | *mmu-miR-1904* | *mmu-miR-27b* | *mmu-miR-433* | *mmu-miR-674* |
| *mghv-miR-M1-5* | *mmu-miR-1905* | *mmu-miR-28* | *mmu-miR-434-3p* | *mmu-miR-675-3p* |
| *mghv-miR-M1-6* | *mmu-miR-1906* | *mmu-miR-2861* | *mmu-miR-434-5p* | *mmu-miR-675-5p* |
| *mghv-miR-M1-7-3p* | *mmu-miR-1907* | *mmu-miR-290-3p* | *mmu-miR-448* | *mmu-miR-676* |
| *mghv-miR-M1-7-5p* | *mmu-miR-190b* | *mmu-miR-290-5p* | *mmu-miR-449a* | *mmu-miR-677* |
| *mghv-miR-M1-8* | *mmu-miR-191* | *mmu-miR-291a-3p* | *mmu-miR-449b* | *mmu-miR-678* |
| *mghv-miR-M1-9* | *mmu-miR-192* | *mmu-miR-291a-5p* | *mmu-miR-449c* | *mmu-miR-679* |
| *mmu-let-7a* | *mmu-miR-1927* | *mmu-miR-291b-3p* | *mmu-miR-450a-3p* | *mmu-miR-680* |
| *mmu-let-7b* | *mmu-miR-1928* | *mmu-miR-291b-5p* | *mmu-miR-450a-5p* | *mmu-miR-681* |
| *mmu-let-7c* | *mmu-miR-1929* | *mmu-miR-292-3p* | *mmu-miR-450b-3p* | *mmu-miR-682* |
| *mmu-let-7d* | *mmu-miR-193* | *mmu-miR-292-5p* | *mmu-miR-450b-5p* | *mmu-miR-683* |
| *mmu-let-7e* | *mmu-miR-1930* | *mmu-miR-293* | *mmu-miR-451* | *mmu-miR-684* |
| *mmu-let-7f* | *mmu-miR-1931* | *mmu-miR-294* | *mmu-miR-452* | *mmu-miR-686* |
| *mmu-let-7g* | *mmu-miR-1932* | *mmu-miR-295* | *mmu-miR-453* | *mmu-miR-687* |
| *mmu-let-7i* | *mmu-miR-1933-3p* | *mmu-miR-296-3p* | *mmu-miR-455* | *mmu-miR-688* |
| *mmu-miR-1* | *mmu-miR-1933-5p* | *mmu-miR-296-5p* | *mmu-miR-463* | *mmu-miR-689* |
| *mmu-miR-100* | *mmu-miR-1934* | *mmu-miR-297a+mmu-miR-466f+mmu-miR-669b* | *mmu-miR-464* | *mmu-miR-690* |
| *mmu-miR-101a* | *mmu-miR-1935* | *mmu-miR-297b-3p* | *mmu-miR-465a-3p* | *mmu-miR-691* |
| *mmu-miR-101b* | *mmu-miR-1936* | *mmu-miR-297b-5p* | *mmu-miR-465a-5p* | *mmu-miR-692* |
| *mmu-miR-103* | *mmu-miR-1937a+mmu-miR-1937b* | *mmu-miR-297c* | *mmu-miR-465b-5p* | *mmu-miR-693-3p* |
| *mmu-miR-105* | *mmu-miR-1937c* | *mmu-miR-298* | *mmu-miR-465c-5p* | *mmu-miR-693-5p* |
| *mmu-miR-106a+mmu-miR-17* | *mmu-miR-1938* | *mmu-miR-299* | *mmu-miR-466a-3p+mmu-miR-466b-3-3p* | *mmu-miR-694* |
| *mmu-miR-106b* | *mmu-miR-1939* | *mmu-miR-29a* | *mmu-miR-466a-5p+mmu-miR-466e-5p* | *mmu-miR-695* |
| *mmu-miR-107* | *mmu-miR-193b* | *mmu-miR-29b* | *mmu-miR-466c-5p* | *mmu-miR-696* |
| *mmu-miR-10a* | *mmu-miR-194* | *mmu-miR-29c* | *mmu-miR-466d-3p* | *mmu-miR-697* |
| *mmu-miR-10b* | *mmu-miR-1940* | *mmu-miR-300* | *mmu-miR-466d-5p* | *mmu-miR-698* |
| *mmu-miR-1186* | *mmu-miR-1941-3p* | *mmu-miR-301a* | *mmu-miR-466f-5p* | *mmu-miR-700* |
| *mmu-miR-1186b* | *mmu-miR-1941-5p* | *mmu-miR-301b* | *mmu-miR-466g* | *mmu-miR-701* |
| *mmu-miR-1187* | *mmu-miR-1942* | *mmu-miR-302a* | *mmu-miR-466h* | *mmu-miR-702* |
| *mmu-miR-1188* | *mmu-miR-1943* | *mmu-miR-302b* | *mmu-miR-466i* | *mmu-miR-703* |
| *mmu-miR-1190* | *mmu-miR-1944* | *mmu-miR-302c* | *mmu-miR-466j* | *mmu-miR-704* |
| *mmu-miR-1191* | *mmu-miR-1945* | *mmu-miR-302d* | *mmu-miR-466k* | *mmu-miR-706* |
| *mmu-miR-1192* | *mmu-miR-1946a* | *mmu-miR-3072* | *mmu-miR-466l* | *mmu-miR-707* |
| *mmu-miR-1193* | *mmu-miR-1946b* | *mmu-miR-3099* | *mmu-miR-467a* | *mmu-miR-708* |
| *mmu-miR-1194* | *mmu-miR-1947* | *mmu-miR-30a* | *mmu-miR-467b* | *mmu-miR-709* |
| *mmu-miR-1195* | *mmu-miR-1948* | *mmu-miR-30b* | *mmu-miR-467c* | *mmu-miR-710* |
| *mmu-miR-1196* | *mmu-miR-1949* | *mmu-miR-30c* | *mmu-miR-467d* | *mmu-miR-711* |
| *mmu-miR-1197* | *mmu-miR-195* | *mmu-miR-30d* | *mmu-miR-467e* | *mmu-miR-712* |
| *mmu-miR-1198* | *mmu-miR-1950* | *mmu-miR-30e* | *mmu-miR-467f* | *mmu-miR-713* |
| *mmu-miR-1199* | *mmu-miR-1951* | *mmu-miR-31* | *mmu-miR-467g* | *mmu-miR-714* |
| *mmu-miR-122* | *mmu-miR-1952* | *mmu-miR-32* | *mmu-miR-467h+mmu-miR-669d+mmu-miR-669l* | *mmu-miR-715* |
| *mmu-miR-1224* | *mmu-miR-1953* | *mmu-miR-320* | *mmu-miR-468* | *mmu-miR-717* |
| *mmu-miR-124* | *mmu-miR-1954* | *mmu-miR-322* | *mmu-miR-469* | *mmu-miR-718* |
| *mmu-miR-125a-3p* | *mmu-miR-1955* | *mmu-miR-323-3p* | *mmu-miR-470* | *mmu-miR-719* |
| *mmu-miR-125a-5p* | *mmu-miR-1956* | *mmu-miR-323-5p* | *mmu-miR-471* | *mmu-miR-720* |
| *mmu-miR-125b-3p* | *mmu-miR-1957* | *mmu-miR-324-3p* | *mmu-miR-483* | *mmu-miR-741* |
| *mmu-miR-125b-5p* | *mmu-miR-1958* | *mmu-miR-324-5p* | *mmu-miR-484* | *mmu-miR-742* |
| *mmu-miR-126-3p* | *mmu-miR-1959* | *mmu-miR-325* | *mmu-miR-485* | *mmu-miR-743a* |
| *mmu-miR-126-5p* | *mmu-miR-1960* | *mmu-miR-326* | *mmu-miR-486* | *mmu-miR-743b-3p* |
| *mmu-miR-127* | *mmu-miR-1961* | *mmu-miR-327* | *mmu-miR-487b* | *mmu-miR-743b-5p* |
| *mmu-miR-1274a* | *mmu-miR-1962* | *mmu-miR-328* | *mmu-miR-488* | *mmu-miR-744* |
| *mmu-miR-128* | *mmu-miR-1963* | *mmu-miR-329* | *mmu-miR-489* | *mmu-miR-758* |
| *mmu-miR-129-3p* | *mmu-miR-1964* | *mmu-miR-33* | *mmu-miR-490* | *mmu-miR-759* |
| *mmu-miR-129-5p* | *mmu-miR-1965* | *mmu-miR-330* | *mmu-miR-491* | *mmu-miR-760* |
| *mmu-miR-1306* | *mmu-miR-1966* | *mmu-miR-331-3p* | *mmu-miR-493* | *mmu-miR-761* |
| *mmu-miR-130a* | *mmu-miR-1967* | *mmu-miR-331-5p* | *mmu-miR-494* | *mmu-miR-762* |
| *mmu-miR-130b* | *mmu-miR-1968* | *mmu-miR-335-3p* | *mmu-miR-495* | *mmu-miR-763* |
| *mmu-miR-132* | *mmu-miR-1969* | *mmu-miR-335-5p* | *mmu-miR-496* | *mmu-miR-764-3p* |
| *mmu-miR-133a* | *mmu-miR-196a* | *mmu-miR-337-3p* | *mmu-miR-497* | *mmu-miR-764-5p* |
| *mmu-miR-133b* | *mmu-miR-196b* | *mmu-miR-337-5p* | *mmu-miR-499* | *mmu-miR-767* |
| *mmu-miR-134* | *mmu-miR-1970* | *mmu-miR-338-3p* | *mmu-miR-500* | *mmu-miR-770-3p* |
| *mmu-miR-135a* | *mmu-miR-1971* | *mmu-miR-338-5p* | *mmu-miR-501-3p* | *mmu-miR-770-5p* |
| *mmu-miR-135b* | *mmu-miR-1981* | *mmu-miR-339-3p* | *mmu-miR-501-5p* | *mmu-miR-7a* |
| *mmu-miR-136* | *mmu-miR-1982* | *mmu-miR-339-5p* | *mmu-miR-503* | *mmu-miR-7b* |
| *mmu-miR-137* | *mmu-miR-1983* | *mmu-miR-340-3p* | *mmu-miR-504* | *mmu-miR-802* |
| *mmu-miR-138* | *mmu-miR-199a-3p* | *mmu-miR-340-5p* | *mmu-miR-505* | *mmu-miR-804* |
| *mmu-miR-139-3p* | *mmu-miR-199a-5p* | *mmu-miR-341* | *mmu-miR-509-3p* | *mmu-miR-871* |
| *mmu-miR-139-5p* | *mmu-miR-19a* | *mmu-miR-342-3p* | *mmu-miR-509-5p* | *mmu-miR-872* |
| *mmu-miR-140* | *mmu-miR-19b* | *mmu-miR-342-5p* | *mmu-miR-511* | *mmu-miR-873* |
| *mmu-miR-141* | *mmu-miR-200a* | *mmu-miR-343* | *mmu-miR-532-3p* | *mmu-miR-874* |
| *mmu-miR-142-3p* | *mmu-miR-200b* | *mmu-miR-344* | *mmu-miR-532-5p* | *mmu-miR-875-3p* |
| *mmu-miR-142-5p* | *mmu-miR-200c* | *mmu-miR-345-3p* | *mmu-miR-539* | *mmu-miR-875-5p* |
| *mmu-miR-143* | *mmu-miR-201* | *mmu-miR-345-5p* | *mmu-miR-540-3p* | *mmu-miR-876-3p* |
| *mmu-miR-144* | *mmu-miR-202-3p* | *mmu-miR-346* | *mmu-miR-540-5p* | *mmu-miR-876-5p* |
| *mmu-miR-145* | *mmu-miR-202-5p* | *mmu-miR-3470a+mmu-miR-3470b* | *mmu-miR-541* | *mmu-miR-877* |
| *mmu-miR-146a* | *mmu-miR-203* | *mmu-miR-3471* | *mmu-miR-542-3p* | *mmu-miR-878-3p* |
| *mmu-miR-146b* | *mmu-miR-204* | *mmu-miR-3472* | *mmu-miR-542-5p* | *mmu-miR-878-5p* |
| *mmu-miR-147* | *mmu-miR-205* | *mmu-miR-3473* | *mmu-miR-543* | *mmu-miR-879* |
| *mmu-miR-148a* | *mmu-miR-206* | *mmu-miR-3474* | *mmu-miR-544* | *mmu-miR-880* |
| *mmu-miR-148b* | *mmu-miR-207* | *mmu-miR-3475* | *mmu-miR-546* | *mmu-miR-881* |
| *mmu-miR-149* | *mmu-miR-208a* | *mmu-miR-34a* | *mmu-miR-547* | *mmu-miR-882* |
| *mmu-miR-150* | *mmu-miR-208b* | *mmu-miR-34b-3p* | *mmu-miR-551b* | *mmu-miR-883a-3p* |
| *mmu-miR-151-3p* | *mmu-miR-20a+mmu-miR-20b* | *mmu-miR-34b-5p* | *mmu-miR-568* | *mmu-miR-883a-5p* |
| *mmu-miR-151-5p* | *mmu-miR-21* | *mmu-miR-34c* | *mmu-miR-574-3p* | *mmu-miR-883b-3p* |
| *mmu-miR-152* | *mmu-miR-210* | *mmu-miR-350* | *mmu-miR-574-5p* | *mmu-miR-883b-5p* |
| *mmu-miR-153* | *mmu-miR-211* | *mmu-miR-351* | *mmu-miR-582-3p* | *mmu-miR-9* |
| *mmu-miR-154* | *mmu-miR-212* | *mmu-miR-361* | *mmu-miR-582-5p* | *mmu-miR-92a* |
| *mmu-miR-155* | *mmu-miR-2132* | *mmu-miR-362-3p* | *mmu-miR-590-3p* | *mmu-miR-92b* |
| *mmu-miR-15a* | *mmu-miR-2133* | *mmu-miR-362-5p* | *mmu-miR-590-5p* | *mmu-miR-93* |
| *mmu-miR-15b* | *mmu-miR-2134* | *mmu-miR-363* | *mmu-miR-592* | *mmu-miR-96* |
| *mmu-miR-16* | *mmu-miR-2135* | *mmu-miR-365* | *mmu-miR-599* | *mmu-miR-374* |
| *mmu-miR-181a* | *mmu-miR-2136* | *mmu-miR-367* | *mmu-miR-615-3p* | *mmu-miR-369-5p* |
| *mmu-miR-181b+mmu-miR-181d* | *mmu-miR-2137* | *mmu-miR-369-3p* | *mmu-miR-370* | *mmu-miR-2138* |
| *mmu-miR-181c* |  |  |  |  |

STable 11: List of Putative Murine Target Genes Obtained from MirDIP

| 2893 Putative Murine Target Genes | | | | |
| --- | --- | --- | --- | --- |
| *RSBN1* | *RB1* | *HERPUD1* | *ASCC2* | *PTBP3* |
| *TRAF6* | *ZBTB44* | *ZNF362* | *SYNGR3* | *GPRIN3* |
| *IRAK1* | *KLF3* | *ZNF384* | *BDNF* | *LRRC58* |
| *NEDD9* | *USP47* | *DYRK3* | *SETD2* | *PPP4R1* |
| *RAB22A* | *RAB11FIP2* | *DNAJC12* | *SLITRK1* | *RPS6KA4* |
| *NR5A2* | *CIAPIN1* | *NEXMIF* | *RYBP* | *ANK2* |
| *PDE4D* | *ARHGAP26* | *ACVR2B* | *PBX2P1* | *ARF1* |
| *HIF1A* | *PPM1E* | *RUNX1T1* | *PPP1R12A* | *SMURF2* |
| *ZMYND11* | *IL34* | *NLK* | *PTPRK* | *SLC25A36* |
| *CREBL2* | *FAM98A* | *DLGAP2* | *DIAPH1* | *GABRA4* |
| *RNF38* | *NAA50* | *PSIP1* | *EMC10* | *GFPT1* |
| *SEPHS1* | *SLC12A6* | *CRELD1* | *N4BP2L1* | *WASF1* |
| *UBE2K* | *SMAD6* | *NR2C1* | *EMC7* | *UBE3A* |
| *SEC23IP* | *CRYBG3* | *SYT11* | *CDC42BPA* | *NDC1* |
| *SATB2* | *SIAH2* | *KLF10* | *PRTG* | *GSK3B* |
| *CDK19* | *VTA1* | *PLEC* | *HBP1* | *ST8SIA3* |
| *TACC1* | *PRKD1* | *EIF5A2* | *AHCYL2* | *FADS6* |
| *PRKCE* | *DIPK2A* | *CACNA2D3* | *MGA* | *NCOA4* |
| *EIF4G2* | *PMP22* | *CAV1* | *NIPAL1* | *CGGBP1* |
| *WWC2* | *RSPO4* | *PELI1* | *POFUT2* | *CAPRIN2* |
| *SGMS1* | *SPRYD7* | *TARDBP* | *ITSN1* | *NEUROD6* |
| *NOVA1* | *AGO3* | *CUL4B* | *TRMT1L* | *CHMP4B* |
| *FOXC1* | *GNA13* | *GGNBP2* | *OSBPL10* | *MAN2A2* |
| *ESR1* | *PI15* | *SLC19A2* | *USP48* | *AMIGO1* |
| *NR3C1* | *SAR1A* | *NHLH2* | *PIEZO2* | *NSD3* |
| *COPS2* | *PDZD2* | *GNAI3* | *FLRT1* | *TIMP3* |
| *TCF12* | *CLTC* | *ADAT2* | *PAFAH1B1* | *BIN2* |
| *MBOAT2* | *CHD9* | *C5orf47* | *DYNLL2* | *VCAN* |
| *ZBTB2* | *GCH1* | *TRIM36* | *ARHGAP36* | *SLC37A2* |
| *SLC9A6* | *RAB14* | *CLLU1* | *ODC1* | *ACSL1* |
| *MAPRE2* | *PBX3* | *SNX1* | *PTGS2* | *TASOR* |
| *RASA1* | *KRR1* | *TNFSF11* | *IBA57* | *ZMYM2* |
| *FOXJ3* | *COL4A4* | *E2F7* | *MDFIC* | *TOX4* |
| *PSD3* | *CDC42SE1* | *BNIP3L* | *ZNF268* | *ADAM29* |
| *PIK3C2A* | *UBP1* | *ZNF292* | *NF1* | *C16orf87* |
| *PEX5* | *SLC17A6* | *TIAM1* | *USF3* | *WASHC5* |
| *TMED10* | *RNF170* | *WNK3* | *MSANTD2* | *DGKI* |
| *ARMC8* | *AP2B1* | *COL11A1* | *TAF9B* | *ZNF185* |
| *SOX11* | *EEA1* | *PAN2* | *KLHDC10* | *ABCD2* |
| *ZCCHC24* | *LRRC8D* | *GAN* | *GNL3L* | *PCBD1* |
| *KANK1* | *GLIS3* | *GXYLT2* | *RANBP10* | *FBXO40* |
| *SERTAD2* | *ITGA6* | *C1orf174* | *AVPR2* | *ATXN7L3B* |
| *MAP4K5* | *RNF144B* | *RAB8A* | *PTGIS* | *FAXC* |
| *HMGA2* | *RAP1A* | *STX5* | *N4BP1* | *RNFT2* |
| *RAP2C* | *EIF4E* | *RBAK* | *NSD1* | *GLCE* |
| *CLASP2* | *TEAD1* | *HNRNPH3* | *MARCHF1* | *PCGF3* |
| *KCNA1* | *CHRM2* | *COL4A5* | *SLC8A1* | *CACNA1C* |
| *CAMK2D* | *KHDRBS1* | *ZFYVE16* | *AAK1* | *PAQR3* |
| *SOX4* | *AP1S1* | *PPP1R11* | *FMNL3* | *ITIH5* |
| *LATS2* | *ZC3H12C* | *NAP1L5* | *PCMT1* | *ST18* |
| *PPP6C* | *RAPGEF5* | *TSHZ1* | *ARHGEF5* | *GLIS2* |
| *RHOBTB1* | *RTKN2* | *AP1G1* | *COL4A1* | *ATMIN* |
| *NUP153* | *SP3* | *EPC2* | *ARF4* | *MITF* |
| *SLC6A6* | *ADCY6* | *BDP1* | *CLOCK* | *ELP1* |
| *ADD3* | *RSPO2* | *NCS1* | *ATP11B* | *NDST1* |
| *USP3* | *TMEM245* | *JADE1* | *TTYH3* | *FOXA1* |
| *PPP1R9A* | *CPD* | *CARD10* | *TAB2* | *SLC2A1* |
| *SMAD2* | *SCN3B* | *GABRB3* | *EVI5* | *SMIM7* |
| *SRSF1* | *EDEM1* | *ALG8* | *FANCI* | *GRIN2A* |
| *CREB5* | *RGL2* | *MAP7* | *DLX5* | *SPEN* |
| *DYRK1A* | *RPS6KA3* | *CPEB3* | *CHFR* | *SSX1* |
| *FRS2* | *NOTCH2* | *KIT* | *ROCK2* | *RBM24* |
| *NEUROD1* | *NEUROG2* | *COL14A1* | *ARHGAP19* | *MAT2A* |
| *BACH2* | *PHF20* | *ZFP62* | *RAP1B* | *TLK1* |
| *HIF1AN* | *RIMS2* | *PAK2* | *TSPYL4* | *ZC3H6* |
| *CEMIP2* | *BAZ2B* | *GMFB* | *GATM* | *PAPSS2* |
| *PPP2R2A* | *ZSWIM6* | *CDC73* | *OSMR* | *USP25* |
| *PTPRD* | *UPF2* | *TRIM44* | *ZNF609* | *TMEM260* |
| *EREG* | *NR2C2* | *FKBP4* | *CDC34* | *DYNC1LI1* |
| *CCND2* | *CHIC1* | *ERLIN1* | *PAPLN* | *EVI5L* |
| *EPHA7* | *PHF20L1* | *MSL2* | *SPTB* | *OLFM3* |
| *HMBOX1* | *TSPYL1* | *SH3KBP1* | *ELOVL5* | *THAP12* |
| *SLC10A3* | *PAPPA* | *MIER1* | *CEP85* | *WASL* |
| *CCNT2* | *BCL2L1* | *DYNC1I2* | *ULK2* | *MYO5C* |
| *TRAPPC8* | *EHBP1* | *ZNF280C* | *KIAA1328* | *PGF* |
| *OXSR1* | *HNRNPA2B1* | *CTDNEP1* | *ID2* | *BICD1* |
| *DCBLD2* | *ARHGAP29* | *XPO4* | *NIT1* | *BAAT* |
| *NUMB* | *PTEN* | *ABTB2* | *MYC* | *PRSS23* |
| *BTG3* | *BRWD3* | *CLINT1* | *MS4A1* | *FN1* |
| *NRBF2* | *RDX* | *NDFIP1* | *SH3TC2* | *DAPK1* |
| *IRF2* | *TDG* | *SERTM1* | *MRPL35* | *RNF103* |
| *FRMD4B* | *TMEM64* | *PNO1* | *SMCR8* | *KIAA1549* |
| *PC* | *LIN28B* | *MEF2D* | *IL1R1* | *KDM6A* |
| *GPR22* | *NCK2* | *CYRIB* | *CAND1* | *SEL1L* |
| *XIAP* | *DDX19A* | *TP63* | *HAUS3* | *SQSTM1* |
| *PHF19* | *CCAR2* | *PCMTD2* | *PALD1* | *RAP1GDS1* |
| *AK4* | *MTMR6* | *UNC45A* | *ZNF587* | *CLIP3* |
| *MAP7D1* | *GLCCI1* | *FBXO3* | *PTPN2* | *CCNE2* |
| *PTGFRN* | *YWHAG* | *APOOL* | *PARP16* | *RAPGEF2* |
| *WNK1* | *ONECUT2* | *PRKAA2* | *PTPRA* | *AMFR* |
| *PHC3* | *CELF1* | *EGR1* | *AMMECR1* | *RNF11* |
| *ATXN1* | *STAU2* | *NFATC3* | *ADAMTSL5* | *BTBD10* |
| *STK38L* | *TTPAL* | *KLHL15* | *GRAMD2B* | *SSX3* |
| *HOXA9* | *CAPZB* | *DCAF15* | *ARMH3* | *TMEM167A* |
| *TNRC6B* | *PLCB1* | *PRX* | *ARHGEF15* | *KCNJ1* |
| *TFRC* | *MARCKS* | *MED4* | *TMEM104* | *PTPN13* |
| *SH2D1A* | *NAV3* | *PEX5L* | *LRRC27* | *ZDHHC9* |
| *HOXA7* | *SYT1* | *PCDH9* | *SYT9* | *STAT3* |
| *PATJ* | *NRG1* | *NOTCH1* | *MAP3K9* | *CASD1* |
| *SPRED1* | *SSR1* | *LAT* | *CELF4* | *EIF4EBP2* |
| *RAB2A* | *SUMF2* | *DRAM1* | *CCSER2* | *CCSAP* |
| *ERG* | *MAP3K2* | *RB1CC1* | *SLC31A1* | *PDE7A* |
| *ACSL4* | *ACTC1* | *LDB2* | *CELF3* | *DSCR4* |
| *TGFBR2* | *LITAF* | *MAP3K12* | *F2R* | *PIP4K2A* |
| *ZEB2* | *HCN1* | *ARL4C* | *MYEF2* | *SORL1* |
| *PUM2* | *DLL1* | *NEO1* | *CMTM6* | *GPC6* |
| *PRKACB* | *C1orf21* | *DENND5B* | *CSN2* | *TFPI* |
| *HOMER1* | *QSER1* | *SPOPL* | *CNOT6* | *DAB2IP* |
| *FOXN3* | *KCNH7* | *MIP* | *MRS2* | *EFHC1* |
| *LBH* | *DCUN1D3* | *PDIA4* | *RAP1GAP2* | *SLCO2B1* |
| *HNRNPD* | *PTPRS* | *KRIT1* | *PSMF1* | *ZNF765* |
| *SAMD4A* | *AZIN1* | *TAOK3* | *NPTN* | *KALRN* |
| *EIF5* | *CSRNP3* | *UBE2H* | *MTHFR* | *HIC2* |
| *ARID1A* | *CELSR3* | *CSF1* | *SLC19A3* | *HIKESHI* |
| *KLHL20* | *DNAJB1* | *ETV1* | *ATP1B3* | *PIFO* |
| *ZDHHC17* | *HCFC2* | *ANKRD44* | *OXR1* | *SMG7* |
| *YWHAE* | *PCDH17* | *BCL11A* | *NCBP3* | *NAB1* |
| *APBB2* | *KAT6B* | *KCTD12* | *NAA25* | *SEMA3G* |
| *PAX9* | *PTPRJ* | *SNX6* | *MAPK1* | *ZNF385A* |
| *RARB* | *SLC24A3* | *DDHD2* | *DLX3* | *XPR1* |
| *RABGAP1* | *JAK2* | *SLITRK4* | *LAMC2* | *PIGO* |
| *PPP3CA* | *RCOR1* | *TRIO* | *ELMOD1* | *LRPAP1* |
| *SUCO* | *PURA* | *ARHGAP5* | *CYCS* | *RPS15A* |
| *CUL1* | *SP4* | *LPL* | *TNFSF4* | *CCNA2* |
| *HMGCS1* | *SASS6* | *ABL2* | *COL5A3* | *STT3B* |
| *IDE* | *GAPVD1* | *HNRNPK* | *SUZ12* | *CD53* |
| *TMEM170B* | *SH3BGRL2* | *XPOT* | *SYT14* | *POLR2K* |
| *RORA* | *MAF* | *CBFA2T3* | *ARF6* | *UNC45B* |
| *ORAI3* | *COL5A1* | *RHOA* | *CMPK1* | *SETD7* |
| *JARID2* | *CHODL* | *AGO1* | *KHNYN* | *PAK5* |
| *PHOX2B* | *CTDSPL* | *KMT2D* | *FOSL1* | *NEMF* |
| *RNF145* | *SRSF10* | *TENM1* | *SCML2* | *ONECUT1* |
| *STARD13* | *SYNJ1* | *SHANK2* | *FBXO45* | *OSTM1* |
| *NCOA1* | *KCTD21* | *DPY19L1* | *ARL5B* | *SERINC5* |
| *CHMP7* | *UBE2N* | *MARCHF7* | *CALCR* | *VDR* |
| *PITX2* | *HAS2* | *PHTF2* | *IER5* | *TOR2A* |
| *ZNF148* | *MYCN* | *AHR* | *COL24A1* | *WHRN* |
| *NDRG3* | *PCDH10* | *SLC30A7* | *LAMP5* | *GOLM2* |
| *TESK2* | *TMPRSS11F* | *BTLA* | *RRAS2* | *SPIRE1* |
| *LCOR* | *IGFBP5* | *ARID2* | *SGK1* | *ZMIZ2* |
| *FCHSD2* | *TNKS2* | *GABPA* | *MAP2K4* | *KCNE3* |
| *ERI1* | *DDHD1* | *ADAM12* | *ARHGEF39* | *CYTH2* |
| *HOXA5* | *POLR1G* | *LYPLA1* | *FOXJ2* | *ACBD5* |
| *HOXC8* | *TMED2* | *MCC* | *GPRC5A* | *USP18* |
| *CORO1C* | *DIS3* | *USP9X* | *GTDC1* | *RALGPS1* |
| *RBBP8* | *MAX* | *PHACTR2* | *KIRREL1* | *ARIH2* |
| *FHIP1B* | *NFE2L2* | *RBBP6* | *ACTR2* | *TDP1* |
| *GATA6* | *CSDE1* | *KPNA3* | *CNGB3* | *CACUL1* |
| *TACC2* | *UBR3* | *HOOK1* | *SORCS1* | *ARFIP2* |
| *PPP6R3* | *AKAP7* | *PDS5B* | *YLPM1* | *NCOR1* |
| *UBTD2* | *MTF1* | *GTF2A1* | *AMMECR1L* | *OTULINL* |
| *GIGYF1* | *SSR3* | *ZBTB43* | *DCC* | *G3BP1* |
| *PKP4* | *MADD* | *ZNF81* | *ATXN7L1* | *DCAF8* |
| *NEDD4* | *MED26* | *SNAP91* | *RGS5* | *PHF24* |
| *SLC43A1* | *PHAF1* | *TP53INP2* | *MCM2* | *ZNF862* |
| *KHDRBS3* | *TPPP* | *PDZRN3* | *PRDM1* | *CDC27* |
| *NRAS* | *NRP2* | *GUCY1B1* | *TMEM50B* | *CYP1B1* |
| *EPHB6* | *SMC6* | *KIF3C* | *NOS1* | *FSHR* |
| *KPNA6* | *ZNF281* | *PABPC3* | *RNF39* | *PCTP* |
| *TAOK1* | *CCDC6* | *SS18* | *CAST* | *RFC1* |
| *ZFP36L1* | *IL6ST* | *SDC2* | *CANX* | *TGOLN2* |
| *DNAJC13* | *BNC2* | *NTM* | *DNAJC18* | *ARHGEF33* |
| *EPHB2* | *SP1* | *MED17* | *RNF111* | *ACTB* |
| *MBNL1* | *SLC11A2* | *RANBP9* | *PGK1* | *INTS13* |
| *LPGAT1* | *NXPH4* | *PLEKHA1* | *UBLCP1* | *TASP1* |
| *DICER1* | *SNRK* | *U2SURP* | *ZMAT3* | *KANSL1* |
| *JAZF1* | *LCORL* | *MAP3K7* | *CAV2* | *SLC26A7* |
| *ZNF367* | *ATP1B1* | *MCTS1* | *MCL1* | *IRGQ* |
| *SLC1A2* | *EPS15* | *DOP1A* | *ZNF253* | *PITPNM2* |
| *SYDE2* | *DNAJC6* | *HEATR5A* | *COL21A1* | *TCEA1* |
| *FAM3C* | *ISM1* | *AKT3* | *SLC16A1* | *TP73* |
| *CDKN2AIP* | *RREB1* | *CPT1A* | *PLGLB1* | *LIN52* |
| *RHOT1* | *ELAVL4* | *FXR1* | *FKBP1B* | *MASP1* |
| *C16orf72* | *PSME1* | *DIAPH2* | *NCKAP1* | *TMEM117* |
| *ATL2* | *MYT1L* | *DPP10* | *FNDC4* | *RFT1* |
| *UBE2D1* | *RTL9* | *CAT* | *PEAK1* | *SPATA5* |
| *PRRX1* | *IRX3* | *PTAR1* | *STK39* | *ZFP90* |
| *TNFAIP3* | *FHIP2A* | *MYCBP2* | *GANAB* | *ZNF234* |
| *MAPK4* | *PLEKHA3* | *SLC38A1* | *TIGAR* | *LRG1* |
| *MFSD14A* | *ABHD2* | *OLIG3* | *RNF185* | *PGM2L1* |
| *RETREG1* | *HHEX* | *ABCA1* | *UHRF2* | *LRFN5* |
| *SIRT1* | *KPNA4* | *MBLAC2* | *FOXRED2* | *TMEM178B* |
| *CDIN1* | *SERINC3* | *PDGFRA* | *LSM12* | *C6orf120* |
| *OTUD4* | *ANKH* | *PSMD11* | *LRRC8B* | *WIPF3* |
| *ANKRD13A* | *NR3C2* | *FZD4* | *MAGT1* | *ZNF106* |
| *MAP3K1* | *ACTR1A* | *AP3S1* | *PRMT3* | *VPS13A* |
| *SEMA6D* | *IPO5* | *TMEM120B* | *ZMPSTE24* | *FUT4* |
| *VAPB* | *MLEC* | *ST13* | *JADE3* | *METTL21A* |
| *CAMK2N1* | *TMX4* | *SYPL1* | *CDK14* | *RPS6KA6* |
| *TM9SF3* | *FBXO30* | *LRRC17* | *ZC3H4* | *CTPS2* |
| *CRIM1* | *KLF15* | *SOCS6* | *DENND5A* | *MAP1A* |
| *KLF12* | *GPM6A* | *RICTOR* | *FBXO21* | *POFUT1* |
| *TRIB2* | *ACSL6* | *EFR3A* | *SPATA8* | *HAUS5* |
| *KDELR2* | *KDM5A* | *CREB1* | *CBLL1* | *GPR137C* |
| *AP2A2* | *JRKL* | *ASAP1* | *DDX54* | *RHEX* |
| *HOOK3* | *GRSF1* | *PHKB* | *RAI14* | *NUP98* |
| *AHCYL1* | *SSH3* | *SON* | *ADCY9* | *ZXDC* |
| *TWF1* | *TDRKH* | *RWDD4* | *HBS1L* | *RPL28* |
| *NCOA7* | *WDR44* | *AGFG1* | *SLC39A9* | *VSX2* |
| *ARID4B* | *PABPC4* | *CHD3* | *SLC22A23* | *CCNB1* |
| *ETS1* | *SLC22A3* | *CAPN10* | *TAFA5* | *STXBP5L* |
| *CACNB2* | *TRIM8* | *GPR85* | *NKAPD1* | *GRM5* |
| *SGK3* | *RIOK3* | *HOXB8* | *FGF2* | *GABRG2* |
| *AKAP1* | *FARP1* | *MZT1* | *ELN* | *WDR26* |
| *CCNJ* | *STARD7* | *CALHM5* | *ALKBH8* | *UBXN7* |
| *SLC37A3* | *MET* | *RAP2A* | *LMO3* | *PLCXD1* |
| *MAP1B* | *FIGN* | *ELF4* | *ABL1* | *STS* |
| *CCN2* | *KLHL23* | *ARL2BP* | *RXFP2* | *COL8A2* |
| *TSPAN12* | *FRMD6* | *SIX4* | *CYBRD1* | *BMPR1B* |
| *VPS26A* | *ELAVL2* | *DDX3X* | *OSBPL11* | *PALS1* |
| *RAB5C* | *KIF2A* | *CBL* | *PLGLB2* | *PDE7B* |
| *CDC42* | *BAHD1* | *MSI2* | *RUBCN* | *ZFP41* |
| *HIPK1* | *BCAT1* | *MACF1* | *RABGGTB* | *KIAA0232* |
| *MMP16* | *TMEM106B* | *CD47* | *NFATC2* | *HEPACAM* |
| *ATF2* | *CXADR* | *CLSTN1* | *TRIP13* | *DUSP4* |
| *QKI* | *GAB2* | *PAX3* | *PPP1R10* | *SEH1L* |
| *VEZT* | *DGKG* | *SLITRK3* | *HABP4* | *CYP7A1* |
| *ILRUN* | *MEAF6* | *FBXO11* | *POLD3* | *TBC1D15* |
| *NR4A2* | *ZNRF2* | *ANKRD40* | *SLC35F3* | *PLEKHB2* |
| *DCAF12* | *TNRC6A* | *TOX* | *MAP3K20* | *ERBIN* |
| *WBP11* | *KCNA4* | *GAS7* | *PKIA* | *DFFA* |
| *STX12* | *RSPO3* | *DIO2* | *SEPTIN7* | *ZNF143* |
| *GXYLT1* | *SINHCAF* | *DPYSL2* | *GRHL1* | *VEZF1* |
| *CDKN1B* | *CLCN5* | *KIF20B* | *ATP6V1G1* | *PALM* |
| *MACIR* | *PDP1* | *HP1BP3* | *BCAT2* | *SMU1* |
| *FRYL* | *LIN54* | *SORBS1* | *POGLUT2* | *ZNF263* |
| *PACRGL* | *CRKL* | *CNOT6L* | *PSME4* | *SIAH3* |
| *CARTPT* | *SLC36A3* | *MARS2* | *OTUD6B* | *ZNF236* |
| *MED1* | *HMGA1* | *CAPN7* | *JAKMIP1* | *SLF2* |
| *AP1S2* | *IGF2BP2* | *BRD1* | *CXCL2* | *AP5B1* |
| *EZR* | *NRF1* | *NEBL* | *ADGRG6* | *CUL3* |
| *STRBP* | *IGF1* | *TRANK1* | *CCDC152* | *ANGPTL4* |
| *EPB41L1* | *BTG2* | *WNT5A* | *PDE3B* | *DISC1* |
| *NLGN1* | *B4GALT6* | *GPATCH2* | *DIXDC1* | *ATRN* |
| *CLCN3* | *RBM12* | *KATNBL1* | *COL1A1* | *PPAT* |
| *PCF11* | *FBXL3* | *TCF20* | *CPM* | *C18orf25* |
| *DMTF1* | *HIVEP2* | *MAGI3* | *ZMYM3* | *TENT4B* |
| *ATP13A3* | *MGAT1* | *LIN7C* | *PCYT1B* | *CDC40* |
| *DAAM2* | *TPM3* | *SEMA4G* | *ANKS1B* | *DDA1* |
| *CPSF6* | *FZD1* | *SPTSSA* | *ICOS* | *PARVA* |
| *FBN2* | *GALC* | *NUP58* | *DMXL1* | *ANGPTL2* |
| *XKR9* | *ARL1* | *VAPA* | *SUMO1* | *KLHL18* |
| *KCMF1* | *PRDM6* | *OLFML2B* | *IRS2* | *GK5* |
| *EPHA5* | *SCHIP1* | *RTL8A* | *GMEB1* | *CLN8* |
| *EGLN3* | *CCDC120* | *ADSS2* | *BMPER* | *SF3B3* |
| *SORBS2* | *DIRAS2* | *RBM26* | *DHX40* | *SHISA9* |
| *ZBTB39* | *OGT* | *CCDC117* | *NECTIN3* | *ZNF273* |
| *RUNX2* | *TRAPPC2* | *CNGA3* | *MEGF9* | *SEC14L4* |
| *HSF2* | *LAMC1* | *VPS54* | *AMACR* | *FKBP5* |
| *CYLD* | *MAN1A1* | *SLC33A1* | *YIPF4* | *CHML* |
| *KCNJ2* | *ZFX* | *AFF2* | *PDPK1* | *GNG12* |
| *ARFGEF1* | *ZC3H12B* | *ZBTB10* | *ATP2A2* | *KLHL32* |
| *ETV6* | *CCDC47* | *ZNF503* | *RAB1A* | *CHRM1* |
| *VSNL1* | *STC1* | *TPP2* | *ANGPTL1* | *KCNK2* |
| *TOMM70* | *PPP3R1* | *CNTNAP1* | *IL21R* | *MDM4* |
| *ISL1* | *SKI* | *FBXO9* | *CCDC121* | *CD200* |
| *BCORL1* | *SH3GL2* | *FRMD4A* | *SPAST* | *EFNA5* |
| *AFF1* | *PPM1K* | *ATP6V0E1* | *FAM222B* | *SPART* |
| *JAG1* | *ARL5A* | *PIK3R1* | *PCNX1* | *PPARA* |
| *ATF1* | *SLC43A2* | *COL6A3* | *NRG3* | *CD96* |
| *FZD3* | *BCL11B* | *CDX1* | *HNRNPA1* | *CDK17* |
| *RCC2* | *REV3L* | *EHMT1* | *SLK* | *ST3GAL5* |
| *KDM2A* | *PAK1* | *ANGPT2* | *ZNF37A* | *SAR1B* |
| *CDON* | *KCNQ5* | *SHISAL1* | *EML6* | *CD46* |
| *ZNRF3* | *TBL1XR1* | *OPCML* | *NMNAT3* | *SEC31A* |
| *TRIM2* | *AGO4* | *MIER3* | *GABPAP* | *KATNIP* |
| *HSPH1* | *NRXN3* | *RAB9A* | *MOB3B* | *ANTXR2* |
| *APPL1* | *FAM13B* | *PTPRZ1* | *RHOH* | *RBM8A* |
| *ERBB4* | *MARCHF5* | *TRPC3* | *DHX57* | *WDR7* |
| *DMD* | *TTLL7* | *TMEM33* | *RUFY3* | *NABP1* |
| *GLRB* | *LYRM2* | *MEOX1* | *FAM13C* | *APH1B* |
| *UBN1* | *FAM53B* | *YES1* | *AKIRIN1* | *KLF8* |
| *SH3BP4* | *INSC* | *SNX27* | *MAPK8* | *CARNMT1* |
| *SLC6A19* | *TBXA2R* | *PALLD* | *ADGRL3* | *KCNH1* |
| *MGAT3* | *LIN28A* | *XKR4* | *MYB* | *FLT1* |
| *NIPBL* | *HAPLN1* | *SAMD8* | *IDH2* | *E2F2* |
| *COL1A2* | *SRGAP3* | *PPP2R5E* | *PHF8* | *CYP2B6* |
| *ABRAXAS2* | *LFNG* | *PTPRT* | *SIPA1L2* | *PACS1* |
| *PHF2* | *DPYSL3* | *OPA1* | *VCPKMT* | *TTC30B* |
| *FRAS1* | *EFNA1* | *PHLPP2* | *DOCK9* | *GPR26* |
| *LMX1A* | *DDX50* | *SOX2* | *MEX3A* | *ACBD3* |
| *ZNF521* | *SCN2A* | *E2F3* | *RANBP2* | *CCDC51* |
| *SSRP1* | *MTFR1* | *MIDEAS* | *TMEM30A* | *CNNM4* |
| *LPAR4* | *DDX5* | *AP4E1* | *KMT2A* | *FOXO1* |
| *NAA15* | *BICRA* | *GRM8* | *PLP1* | *SBK1* |
| *TMEM248* | *ARRDC3* | *BRCA1* | *MPP2* | *JCAD* |
| *PTPRG* | *KMT2C* | *LRP6* | *CTDSPL2* | *UBTF* |
| *PTMA* | *MAFG* | *RBBP9* | *SLC22A5* | *DSG3* |
| *REEP1* | *SRSF6* | *CPS1* | *ADGRE3* | *RBM15B* |
| *ATP8A1* | *PHF13* | *FGF14* | *SNIP1* | *PIK3CG* |
| *MAP4K3* | *BACH1* | *HIGD1A* | *PRLR* | *ARX* |
| *ALCAM* | *BICD2* | *KLHL28* | *SLC2A12* | *ZMIZ1* |
| *ATM* | *DIP2A* | *COPS8* | *ATP8B4* | *TMEM196* |
| *SUPT16H* | *SMC1A* | *YPEL5* | *C1orf94* | *DCTN4* |
| *CNKSR3* | *YWHAB* | *SEMA4B* | *PAPOLA* | *ARPP19* |
| *NUFIP2* | *TRPC4* | *SMARCC1* | *NPAS3* | *DKK3* |
| *FAM168B* | *ECT2* | *FYN* | *ELK4* | *ODAPH* |
| *PARD6B* | *DUSP7* | *TMEM143* | *FAM135A* | *PKNOX2* |
| *ADAMTS5* | *RC3H1* | *PDCD6IP* | *CDK6* | *CFLAR* |
| *TP53INP1* | *LASP1* | *RPAP2* | *WIPF2* | *MYO18A* |
| *NR6A1* | *ANKRD50* | *SPOCK3* | *FABP3* | *FAM214B* |
| *SF3B1* | *BCOR* | *NELFE* | *SLC1A4* | *CFAP44* |
| *DAG1* | *ATXN7* | *UBE2W* | *CA10* | *SPATS2* |
| *CCNC* | *KLF13* | *FGL2* | *RNF138* | *TMEM164* |
| *TRIP12* | *LYPD6* | *HMGB1* | *EFCAB14* | *CPEB2* |
| *ZBTB47* | *SMG1* | *CHMP2B* | *TET2* | *ZNF561* |
| *CDC42EP3* | *PPP1CC* | *FEM1C* | *TMED5* | *STRN3* |
| *KDM5B* | *ZDHHC13* | *GFRA2* | *AFTPH* | *ZNHIT6* |
| *MLLT3* | *PTHLH* | *ABR* | *PRKCSH* | *POGK* |
| *ELAVL1* | *FAM229B* | *B3GALNT1* | *ATAD2B* | *MANEAL* |
| *MINAR1* | *ZNF644* | *PDIK1L* | *ANP32E* | *CRTAP* |
| *MYO5A* | *BVES* | *COL13A1* | *RNF141* | *WASF3* |
| *RFX7* | *ZNF652* | *GSPT1* | *UBE2J1* | *PRKAB2* |
| *CHD5* | *RAB27A* | *H6PD* | *PRKAG1* | *LRRC59* |
| *C9orf72* | *GLYR1* | *FPGT* | *GPR37* | *REEP3* |
| *STK35* | *BTBD3* | *ALX4* | *NOTCH2NLA* | *KATNAL1* |
| *MYLK* | *NEGR1* | *SVIL* | *TMEM135* | *RAB18* |
| *FBXW2* | *DNAJC5G* | *VASN* | *NSL1* | *DDX31* |
| *EMP1* | *LMAN2* | *ILF3* | *KCNG3* | *RXRB* |
| *PPP6R2* | *FUBP3* | *DKK2* | *SLFN5* | *TRIM14* |
| *NCOA6* | *HDAC9* | *ATRNL1* | *TPBG* | *PLBD2* |
| *PEG10* | *MGAT4A* | *DNMT3A* | *SECISBP2L* | *GALNT12* |
| *SEC24D* | *HDLBP* | *TRAK2* | *KIF1B* | *STXBP4* |
| *ADIPOR2* | *AK2* | *DCUN1D4* | *GLP1R* | *APBA1* |
| *RAB8B* | *PCDH19* | *PMEPA1* | *MYO1D* | *ZFHX2* |
| *C5orf24* | *SORT1* | *MRPS27* | *AMER1* | *CDC23* |
| *ZBTB4* | *TNPO1* | *PPP1R14D* | *PABIR2* | *FZD5* |
| *CDH2* | *NAP1L1* | *PLEKHF2* | *CEP170* | *KLK7* |
| *DNAJB9* | *SNX4* | *LORICRIN* | *DNAJB4* | *RABGAP1L* |
| *CHD7* | *WDFY3* | *RAB23* | *PROM2* | *CDH15* |
| *NFAT5* | *EZH2* | *TAB3* | *BABAM2* | *ADAMTS18* |
| *HIPK3* | *SLC5A3* | *FHOD3* | *SLC4A4* | *TTC39C* |
| *ZNF423* | *CAPZA2* | *TSHZ3* | *CSNK1G3* | *ZNF814* |
| *CSNK2A1* | *PPP1R3D* | *PDE10A* | *SNX13* | *LEP* |
| *RPS6KA5* | *HBEGF* | *EFNB2* | *CDC7* | *KCNE4* |
| *VGLL3* | *LRRC15* | *KPNA1* | *DUSP1* | *PRRC1* |
| *TAPT1* | *PBX1* | *LDLRAD4* | *CERS6* | *ZNF618* |
| *MIPOL1* | *H3-3A* | *ELOA* | *PBOV1* | *WDR72* |
| *TRIOBP* | *TEX2* | *PPP3CB* | *GABRB2* | *TRAK1* |
| *SRSF7* | *MDGA1* | *MAP3K13* | *ZNF576* | *USP28* |
| *DOCK4* | *XKR6* | *ACER2* | *MEGF10* | *PAX7* |
| *ADARB1* | *ROBO1* | *GOLGA1* | *ASCL1* | *CASC3* |
| *DDX19B* | *HERPUD2* | *TSTD2* | *STAMBPL1* | *FAM8A1* |
| *MTMR4* | *ZC3HAV1* | *PPTC7* | *RNGTT* | *KIAA0930* |
| *ANKRD13C* | *DTNA* | *STK26* | *IGF2* | *ITPRIPL2* |
| *ZNF654* | *NMNAT2* | *FZD6* | *IGDCC3* | *NT5C2* |
| *COL3A1* | *KDM7A* | *MED28* | *SYT6* | *CCDC69* |
| *F3* | *DUSP6* | *PLCB4* | *SRSF3* | *CD83* |
| *MARK1* | *SNX16* | *RPP14* | *NRCAM* | *LRATD2* |
| *FNDC3B* | *FBXO32* | *SMARCA5* | *DCP2* | *RNF169* |
| *CD2AP* | *FUT8* | *LIMS1* | *GALNT1* | *STEAP4* |
| *DVL3* | *GOSR1* | *ROBO2* | *TMEM236* | *SSBP3* |
| *CASK* | *EXOC5* | *RNF19A* | *C4orf46* | *DNM3* |
| *DIP2C* | *ZFP30* | *PAPOLG* | *CRK* | *PLAG1* |
| *WWC3* | *TLN2* | *NEK6* | *VASH2* | *HEG1* |
| *SMIM13* | *FBXL5* | *UBE2D3* | *VANGL2* | *PNP* |
| *ELOVL6* | *VCP* | *DLK2* | *YAF2* | *URB2* |
| *NUAK1* | *PANK3* | *MEGF11* | *NAV2* | *FOS* |
| *UBE2Z* | *TRPS1* | *RSRC2* | *CCT8* | *C18orf63* |
| *KLF7* | *ACTL6A* | *SLC38A2* | *EIF1AX* | *TRIM66* |
| *ZFP91* | *YTHDF3* | *NXPE3* | *NADK2* | *MYORG* |
| *DCP1A* | *REST* | *EXOC8* | *SLC18A1* | *ZNF208* |
| *TCF7L2* | *MAFB* | *SREK1* | *PLAGL1* | *TIPRL* |
| *LMO4* | *ESYT2* | *KCNA7* | *SLC35B3* | *ACP3* |
| *IGSF1* | *IKZF2* | *SS18L1* | *MDGA2* | *PDK3* |
| *IPMK* | *ZFAND5* | *NCOA3* | *UHRF1BP1* | *TFAP2A* |
| *EBF1* | *CD80* | *PBRM1* | *TENT5A* | *SNX29* |
| *PPARGC1A* | *STK40* | *SEMA3C* | *FAM120C* | *EPB41L3* |
| *SEC61A2* | *MLXIP* | *ERLEC1* | *PCDH11Y* | *TFDP2* |
| *LSM5* | *COPS7B* | *PFN2* | *STX6* | *PPP2R1B* |
| *MEF2C* | *FBXO38* | *TBC1D20* | *NSF* | *CDH18* |
| *FOXN2* | *ZDHHC21* | *KCTD1* | *BEND4* | *CXorf38* |
| *SRPK2* | *KLF2* | *MCFD2* | *CHIC2* | *KCNAB1* |
| *RHOBTB3* | *VCPIP1* | *ARID4A* | *YIPF5* | *ADAMTSL1* |
| *CACNG4* | *SLC7A11* | *GLTP* | *ORAI2* | *TTYH2* |
| *RUNX1* | *PRKCD* | *SKAP2* | *FRMD5* | *TRPM8* |
| *TOX3* | *ACVR1* | *RBM47* | *ALG9* | *CYB5R1* |
| *MBNL2* | *CCNG2* | *TRIM37* | *ARHGEF12* | *PSG4* |
| *UBE2D2* | *PURB* | *MFSD14B* | *PRRC2C* | *GTPBP3* |
| *ANKFY1* | *SMIM15* | *NRXN1* | *AFF4* | *SRD5A3* |
| *FNBP1L* | *LPP* | *BLMH* | *ADK* | *CRYBG1* |
| *MMGT1* | *FAM91A1* | *MBTD1* | *WASHC4* | *CPSF2* |
| *AUTS2* | *RAP2B* | *PARP11* | *TUBB2A* | *CCDC83* |
| *DBT* | *MSN* | *ACVR2A* | *SMC5* | *MTPN* |
| *WDR5* | *TEX261* | *FAM104A* | *ATRX* | *HRH4* |
| *FBXO28* | *BRWD1* | *AGFG2* | *BAIAP2L1* | *HNRNPC* |
| *DCAF7* | *RAB21* | *MFAP3* | *RCAN2* | *MAGOHB* |
| *PITPNA* | *ARPC5* | *NF2* | *KCNIP2* | *JMJD1C* |
| *BHLHE22* | *PDPR* | *TSC22D3* | *ZC3H15* | *FYCO1* |
| *YTHDC1* | *VPS36* | *MTCL1* | *KSR2* | *TRPC1* |
| *GCNT2* | *RBPMS* | *RTF1* | *EPHA4* | *RNF123* |
| *SESTD1* | *RPS6KB1* | *SNX22* | *CNN3* | *ATXN7L3* |
| *POU3F2* | *NXT2* | *RAD23B* | *DDX6* | *ELOVL7* |
| *BBX* | *NUCKS1* | *TM9SF4* | *MB21D2* | *KRT36* |
| *VAV3* | *GIGYF2* | *IGDCC4* | *SRGAP1* | *TRIT1* |
| *SGIP1* | *PAXIP1* | *THUMPD1* | *TRPM7* | *TENM3* |
| *RIMS3* | *AGPAT5* | *ZBTB41* | *DYNLT5* | *ENAH* |
| *BPTF* | *PDGFC* | *USP34* | *OTUD7A* | *FGB* |
| *ASXL2* | *ENOX2* | *GGT7* | *CLEC2D* | *CABLES2* |
| *XYLT2* | *RALGPS2* | *CRISPLD1* | *MTAP* | *SPECC1* |
| *AP3M1* | *TIMELESS* | *WDFY1* | *JPT1* | *SLC9A7* |
| *MAP2* | *DCUN1D1* | *CBLN4* | *ALX1* | *GLB1L2* |
| *RNF144A* | *ALS2* | *ANKRD34A* | *TMEM87A* | *KRTAP8-1* |
| *XRCC5* | *SMARCA2* | *CDC14B* | *DAAM1* | *GATD1* |
| *ZFHX3* | *LONRF1* | *JKAMP* | *ASPH* | *KRT6A* |
| *METAP1* | *SNTB2* | *PRKCI* | *NETO1* | *GASK1B* |
| *PRDM2* | *ZNF532* | *RBMS2* | *TMTC3* | *CDCA2* |
| *FGF7* | *HGF* | *NR4A3* | *ZBTB38* | *DTYMK* |
| *ELL2* | *SLC4A7* | *TIMM13* | *GJD3* | *ZNF511* |
| *MAP1LC3B* | *VPS26B* | *TRIM33* | *TBC1D12* | *ZNF780A* |
| *TMEM145* | *CEP85L* | *MIEF1* | *ARID1B* | *STXBP1* |
| *ANKIB1* | *GPHN* | *HNRNPDL* | *LPAR1* | *HEY1* |
| *ARHGEF2* | *CUL5* | *TNRC6C* | *USP1* | *OPRM1* |
| *RASSF3* | *ACKR2* | *TRPM3* | *ITGAV* | *PPIF* |
| *FNDC3A* | *SLC39A10* | *ASIC1* | *CEP135* | *MTRNR2L3* |
| *ATP2B1* | *SEC24A* | *MED14* | *TPD52L3* | *INTS1* |
| *LRP1B* | *LIF* | *USP6NL* | *PPP4R3B* | *ANO3* |
| *TTYH1* | *VMA21* | *SEPTIN11* | *MEST* | *CFAP77* |
| *H3-3B* | *HELZ* | *SCAF11* | *MYH10* | *PBX4* |
| *EFNB3* | *DACH1* | *YWHAZ* | *ARIH1* | *AMIGO2* |
| *GCLC* | *DCDC2* | *ASAP2* | *ANO4* | *ITGA11* |
| *TMEM39A* | *IGF2BP1* | *SREK1IP1* | *ZDHHC6* | *SLC6A2* |
| *CAMK1* | *C17orf78* | *TMEM169* | *ZC3H14* | *SPG7* |
| *ATP11C* | *ADAMTS1* | *EPC1* | *CYB561D1* | *FIZ1* |
| *HS2ST1* | *ERLIN2* | *GRK2* | *PCNP* | *KRT86* |
| *FAM126A* | *SLC2A4* | *NT5DC3* | *HTR2C* | *OPA3* |
| *PCDH8* | *POGLUT3* | *APOA5* | *SHC1* | *MARK4* |
| *PPP1R15B* | *PRRG1* | *TGFBR1* | *DESI2* | *MXRA5* |
| *ACTR3* | *SERP1* | *ZIC3* | *NKD1* | *ELP5* |
| *DLG5* | *OCLN* | *HNRNPA0* | *MSX1* | *C10orf71* |
| *PRKAR1A* | *ZNF512B* | *RUFY2* | *PDCD10* | *SPTBN1* |
| *ZFPM2* | *CREBRF* | *GPR3* | *GNAI1* | *EPAS1* |
| *LRP12* | *PRKAR2A* | *CEMIP* | *FUT9* | *SNCG* |
| *STK4* | *MMP14* | *ADAMTS3* | *KLF9* | *GFRA1* |
| *SOCS5* | *EDNRB* | *MOSMO* | *PTCH1* | *CYP11B2* |
| *RAB10* | *PRELID3B* | *L2HGDH* | *VIPAS39* | *PRSS22* |
| *LRIG2* | *HNRNPA3* | *EPHA3* | *TMEM47* | *PPIL2* |
| *TMX1* | *RIPOR2* | *ZBTB26* | *WWTR1* | *FBXO22* |
| *HGSNAT* | *PTPRB* | *RBL2* | *FGD5* | *DRAXIN* |
| *LARP4* | *XPNPEP3* | *TET3* | *KIF5B* | *PODXL* |
| *PHLPP1* | *PRDM16* | *AFAP1L2* | *ETFBKMT* | *GPX4* |
| *TBPL1* | *PIP4P2* | *IREB2* | *AKAP2* | *BCL2L13* |
| *MON2* | *FAM53C* | *SMC3* | *ARID5B* | *FBXL16* |
| *CAMTA1* | *CBFB* | *SWAP70* | *PHIP* | *SDCCAG8* |
| *SMOC1* | *GPI* | *LNPK* | *KLHL2* | *ZDHHC8* |
| *HOXB7* | *LBR* | *SLC49A4* | *MTMR9* | *AKAP17A* |
| *MYO10* | *SYNCRIP* | *RTL8C* | *CD40LG* | *GRHL3* |
| *TMEM168* | *UBR5* | *CA1* | *CHL1* | *F2RL1* |
| *FBXW7* | *MRFAP1* | *AP3M2* | *PGAP1* | *PLXNC1* |
| *SIKE1* | *TSC22D2* | *TMEM214* | *AMOTL1* | *RAB3GAP1* |
| *IGF2BP3* | *NSD2* | *LRRTM2* | *CREB3L2* | *SLC12A9* |
| *CTNND2* | *DAZAP2* | *MOCS1* | *BAG5* | *ULBP2* |
| *GRB10* | *NACC1* | *RAVER2* | *SC5D* | *AKT1S1* |
| *LHX8* | *CPEB1* | *SPIN1* | *DENND1B* | *GRM7* |
| *MGST3* | *SPRY1* | *GRID1* | *GOLPH3* | *MRPS2* |
| *GEM* | *PNPLA2* | *GALNT10* | *SMIM14* | *ATP5MF* |
| *CFL2* | *SERBP1* | *FGFR2* | *VPS13B* | *SYS1* |
| *AEBP2* | *PTP4A3* | *TOR1AIP1* | *DNAJC21* | *KCNN4* |
| *G3BP2* | *SLC4A10* | *SLMAP* | *B2M* | *GRM1* |
| *ADGRB3* | *CENPV* | *ATOH8* | *AP3D1* | *EDA* |
| *IGFBP3* | *SESN3* | *SF1* | *BACE1* | *EFR3B* |
| *TNS1* | *TYMS* | *CDC14A* | *RAB5B* | *YDJC* |
| *NNT* | *SLC16A10* | *TMEM30B* | *IL16* | *PCDH7* |
| *BIRC6* | *ERI2* | *GDNF* | *RAB11B* | *ACOT13* |
| *HECA* | *THRB* | *WDR47* | *IL10* | *PPP1R7* |
| *CALM1* | *RMND5B* | *EEF2K* | *ST8SIA2* | *C15orf62* |
| *MVB12B* | *FBN1* | *STMP1* | *PAG1* | *PHACTR3* |
| *ANGPT1* | *SLC6A15* | *STK24* | *JAM2* | *ZNF467* |
| *SMAD4* | *UBE2G1* | *MAGI2* | *KCNA3* | *CTDP1* |
| *CHN2* | *DCX* | *IL13RA1* | *POLR2E* | *SGMS2* |
| *NFIB* | *CNIH1* | *BTF3L4* | *TFEC* | *MGRN1* |
| *TMEM161B* | *SELE* | *SCN3A* | *TMEM128* | *RELCH* |
| *SLTM* | *CPNE8* | *PRKD3* | *RIC8B* | *MUC17* |
| *CCNY* | *HOXB6* | *ST7L* | *SGCD* | *TNK2* |
| *ENC1* | *CDS1* | *FUT6* | *PTPRR* | *PCID2* |
| *ETNK1* | *ETNK2* | *MLLT10* | *GDF11* | *PIP5K1C* |
| *VWA8* | *USP38* | *MTA2* | *ZNF207* | *MTRNR2L1* |
| *LARP4B* | *PANK2* | *RALGAPB* | *MED13* | *ARHGEF18* |
| *LSM14B* | *MEF2A* | *EML4* | *EBF2* | *CKB* |
| *RGS4* | *CCNG1* | *TSHZ2* | *RIMBP2* | *KIAA1671* |
| *CAPRIN1* | *BOD1L1* | *MLF1* | *ABI2* | *HYI* |
| *PHF6* | *NEMP1* | *IST1* | *GUF1* | *GLI2* |
| *ARHGAP28* | *MARCHF6* | *TRIM39* | *NUP50* | *CLEC10A* |
| *ARCN1* | *LTN1* | *GSK3A* | *CHST2* | *IFT122* |
| *KDM2B* | *USP15* | *BCL9* | *DCK* | *CHI3L2* |
| *DHH* | *ZFC3H1* | *CNOT4* | *ARFGEF3* | *MCHR2* |
| *LEMD3* | *GRIA3* | *ATP2B4* | *HIPK2* | *SNTB1* |
| *UBA6* | *MAML1* | *KIF26B* | *CDCP1* | *KCNQ3* |
| *SSH1* | *NR2F2* | *CADM1* | *ING3* | *LGALS3BP* |
| *OSBPL6* | *JPH1* | *STK38* | *TCF7* | *ZNF681* |
| *ALPL* | *LANCL1* | *GATAD1* | *ZNF451* | *LRRC1* |
| *SLC16A2* | *PUM1* | *POU6F1* | *DHCR24* | *TRIM26* |
| *SAMD5* | *RBM7* | *SEMA3A* | *NRN1* | *EN1* |
| *CREG1* | *SOAT1* | *SDC3* | *KPNB1* | *IQCE* |
| *STAM2* | *SLC6A8* | *ZNF831* | *TAPBP* | *PDXDC1* |
| *POU2F1* | *FNIP1* | *FOXL1* | *NOD2* | *NLGN3* |
| *THBS1* | *CDV3* | *CEBPA* | *PAXBP1* | *METTL22* |
| *SOX5* | *CAMSAP2* | *AZI2* | *SEC23A* | *SLC44A5* |
| *XYLT1* | *CCK* | *UBN2* | *SLC35F5* | *PDLIM7* |
| *NBEA* | *CD34* | *SNPH* | *PARP8* | *PPM1F* |
| *BAZ2A* | *HECW2* | *LIMCH1* | *AP1S3* | *POLR1A* |
| *NBR1* | *BEND3* | *TAF5* | *NFKBIZ* | *TMEM51* |
| *ROCK1* | *UBASH3B* | *PRKAA1* | *RAB3C* | *MTRNR2L5* |
| *MPZ* | *ITCH* | *STAG2* | *NFASC* | *PDE2A* |
| *SDCBP* | *UHMK1* | *FAM169A* | *RNF125* | *MRTFA* |
| *ITPR1* | *AGPAT3* | *DLGAP4* | *SYBU* | *CNTN2* |
| *PAX6* | *ATXN7L2* | *PHF21A* | *ZEB1* | *FAM76B* |
| *SH3PXD2A* | *SOX6* | *PLPP6* | *ADNP* | *ATE1* |
| *CHP1* | *MYBL1* | *FBRS* | *CD164* | *HUS1* |
| *TMOD3* | *FOXP1* | *ZNF248* | *WDR55* | *LRP5* |
| *ULK1* | *CCDC88A* | *COL12A1* | *KCTD9* | *TTC7B* |
| *CELF2* | *ENTPD1* | *PLSCR4* | *SH3RF3* | *TNS2* |
| *CNOT7* | *EIF4ENIF1* | *TLL1* | *CBX5* | *NOX3* |
| *PKN2* | *PRDM5* | *EPB41L4B* | *BPGM* | *CCDC102A* |
| *MED12L* | *TMED7* | *SEC14L2* | *SELENOT* | *HLA-DQA1* |
| *ZCCHC3* | *INO80D* | *FOXP2* | *PPP2CA* | *RAB38* |
| *TIA1* | *LRRC7* | *CALM3* | *TMEM200B* | *SRSF8* |
| *ZNF536* | *RAB5A* | *FAM210A* | *UBL3* | *KERA* |
| *ESRRG* | *UBR1* | *API5* | *ZNF608* | *CTSA* |
| *BMPR1A* | *MACROH2A1* | *NEDD4L* | *EOGT* | *DYNC1LI2* |
| *EYA4* | *MEX3C* | *PTP4A1* | *NRIP3* | *PRKCB* |
| *TRERF1* | *GALNT13* | *MOB1B* | *NRG2* | *C15orf40* |
| *EBF3* | *SPOUT1* | *TCF7L1* | *LCP1* | *BRSK1* |
| *BLCAP* | *KLF6* | *PLEKHA5* | *SLC25A5* | *APC2* |
| *CFTR* | *SNX2* | *SLC7A6* | *KCTD3* | *CDH11* |
| *POLR3D* | *SIK3* | *UBA52* | *LUZP1* | *LRRN4CL* |
| *BCL2* | *PPP1CB* | *MEX3B* | *RGL1* | *TMEM181* |
| *PHF3* | *CLCN6* | *CDCA7* | *KLHL7* | *NMT1* |
| *CTNNBIP1* | *KCNK1* | *KAT6A* | *ATG7* | *HLA-DQA2* |
| *ARL8B* | *CPEB4* | *KLF4* | *BCL2L11* | *DDB1* |
| *BTBD7* | *CALM2* | *LRRFIP1* | *RAD54B* | *CSNK1G2* |
| *TET1* | *RAB11A* | *AQP4* | *CCDC171* | *MCTP2* |
| *SLC16A6* | *RIN2* | *INPP5A* | *RBFOX2* | *CMTM4* |
| *ZFHX4* | *EIF3J* | *OTX1* | *PLEKHG4B* | *MTRNR2L6* |
| *EFEMP2* | *SIM2* | *HMG20A* | *PTPRF* | *PLD6* |
| *RAB6B* | *SPCS3* | *SARAF* | *DYRK2* | *SART3* |
| *HAND1* | *GPCPD1* | *TXNDC9* | *ACSL3* | *HERC5* |
| *GALNT7* | *MED13L* | *TMBIM6* | *N4BP2L2* | *FCGR2B* |
| *OTULIN* | *MXI1* | *ZCCHC14* | *OSBPL9* | *PDPN* |
| *LRIG3* | *WDR37* | *DCLK1* | *POU4F1* | *SOX9* |
| *SMAP2* | *CELSR2* | *SPINDOC* | *QDPR* | *MAP1LC3C* |
| *HOXC6* | *SLC39A6* | *RBM39* | *MPZL1* | *COX7A2* |
| *HACD3* | *ST3GAL1* | *ARPP21* | *TANK* | *SUMO3* |
| *APP* | *CENPK* | *TNKS* | *ELOC* | *SEPSECS* |
| *CEP350* | *NME4* | *BTBD1* | *NIBAN1* | *AFG1L* |
| *MTMR7* | *HMCN1* | *TSC22D1* | *PERP* | *IL18R1* |
| *RBMS3* | *CADM2* | *IRF2BP2* | *VAMP3* | *SLC66A1* |
| *HIF3A* | *TANC2* | *COL5A2* | *UMODL1* | *DLG1* |
| *ZBTB34* | *ZZZ3* | *RNF24* | *ANKS1A* | *NFYA* |
| *NFIA* | *PPIP5K2* | *GRPEL2* | *PALS2* | *KDM4A* |
| *GABBR2* | *ATXN2L* | *TJP1* | *EIF2S2* | *MN1* |
| *ZNF516* | *NREP* | *CKS1B* | *SOS1* | *BCL2L15* |
| *CTCF* | *NAV1* | *PPM1D* | *EVA1A* | *PTH* |
| *NTRK2* | *SLC35A3* | *TRAM1* | *SLC35D1* | *VLDLR* |
| *CSRNP2* | *YOD1* | *SLC1A1* | *CELF6* | *LSM14A* |
| *GATAD2B* | *ARHGAP21* | *XPO1* | *NHLRC2* | *MARF1* |
| *NR1H2* | *ABCB9* | *OPRK1* | *CC2D1B* | *PGM2* |
| *CDYL* | *KCTD15* | *POLE3* | *APPBP2* | *SHANK1* |
| *BZW1* | *TBC1D14* | *GUCD1* | *SLC25A13* | *VPS53* |
| *PTMS* | *CDK2* | *MAPK1IP1L* | *TXLNG* | *RIN3* |
| *ZNF704* | *ITPRID2* | *EIF4A1* | *GABPB1* | *AP5M1* |
| *IGF2R* | *MORF4L1* | *FBXO33* | *KAT7* | *CARM1* |
| *IPO8* | *MAP3K3* | *MXD1* | *KLRG1* | *PSMA6* |
| *COX5A* | *ARPC2* | *PRUNE1* | *INSM1* | *ERCC6L2* |
| *ANK3* | *TFCP2* | *INTS6L* | *BRD4* | *MARCHF8* |
| *XRN1* | *ZIC5* | *PID1* | *TMOD2* | *PPP2R2D* |
| *NKAIN2* | *PRPF40A* | *SH3GLB1* | *HSPA13* | *MMP24OS* |
| *HLTF* | *PAIP1* | *CSMD1* | *DESI1* | *TSC1* |
| *TPD52L2* | *BRD3* | *SPTAN1* | *SGPP1* | *MTMR2* |
| *C21orf91* | *SLC2A3* | *RASGRP1* | *SOS2* | *POU4F2* |
| *PPM1A* | *UBE2I* | *TTBK1* | *MAVS* | *KCTD10* |
| *LRP8* | *DPY19L3* | *WSB1* | *SCN4B* | *TAF5L* |
| *NELFA* | *MYT1* | *CD209* | *TOB1* | *GALNT16* |
| *GLS* | *SMAD5* | *BASP1* | *CD69* | *GRIN2B* |
| *LRP2* | *DLC1* | *SNN* | *SYNC* | *ADGRG1* |
| *CUL4A* | *ING5* | *MORF4L2* | *PTP4A2* | *PCDH1* |
| *ANKRD12* | *AKAP6* | *SCN1A* | *PHACTR1* | *ZBTB40* |
| *YY1* | *SSBP2* | *RND3* | *PALM2AKAP2* |  |
| *KLHL24* | *NKRF* | *FAM171A1* | *SPOCK1* |  |

STable 12: Genes Input into STRING Database

| Genes in Common Between Human, Human Homologs and Cell Cycle | | | |
| --- | --- | --- | --- |
| *PPP6C* | *DYRK1A* | *PAFAH1B1* | *RCC2* |
| *AKAP9* | *E2F2* | *PHF20* | *RFC1* |
| *AKT3* | *E2F3* | *PHF8* | *SMARCA5* |
| *ANKRD28* | *GSK3B* | *POLD3* | *SMC1A* |
| *ATRX* | *HAUS3* | *POLE3* | *SPAST* |
| *BRCA1* | *HUS1* | *POLR2E* | *STAG2* |
| *CCND2* | *IST1* | *PPP1CB* | *SUMO1* |
| *CCNE2* | *JAK2* | *PPP1CC* | *TAOK1* |
| *CDC27* | *KIF2A* | *PPP2CA* | *TFDP2* |
| *CDK2* | *LBR* | *PPP2CB* | *TNPO1* |
| *CDK6* | *LEMD3* | *PPP2R1B* | *TUBB2A* |
| *CENPK* | *MAPK1* | *PPP2R2A* | *UBE2D1* |
| *CHMP2B* | *MAX* | *PPP2R5E* | *UBE2N* |
| *CKS1B* | *MCM2* | *PPP6R3* | *XPO1* |
| *CSNK2A1* | *NIPBL* | *PRKCB* | *YWHAG* |
| *CUL1* | *NSD2* | *PSMD11* | *YWHAZ* |
| *DYNC1LI2* | *NSL1* | *PSME4* | *ZNF385A* |
| *DYNLL2* | *NUP153* | *RANBP2* |  |
